# Biomimetic enantioselective synthesis of *β,β*-difluoro-*α*-amino acid derivatives

**DOI:** 10.1038/s42004-021-00586-z

**Published:** 2021-10-22

**Authors:** Qiupeng Peng, Bingjia Yan, Fangyi Li, Ming Lang, Bei Zhang, Donghui Guo, Donald Bierer, Jian Wang

**Affiliations:** 1 School of Pharmaceutical Sciences, Department of Chemistry, Collaborative Innovation Center for Diagnosis and Treatment of Infectious Diseases Key Laboratory of Bioorganic Phosphorous Chemistry and Chemical Biology (Ministry of Education), Tsinghua University, 100084 Beijing, China; 2 Department of Medicinal Chemistry, Bayer AG, Aprather Weg 18A, 42096 Wuppertal, Germany; 3 Present Address: Leibniz-Forchungsinstituts für Molekulare Pharmakologies (FMP), 13125 Berlin, Germany

**Keywords:** Organocatalysis, Asymmetric catalysis

## Abstract

Although utilization of fluorine compounds has a long history, synthesis of chiral fluorinated amino acid derivatives with structural diversity and high stereoselectivity is still very appealing and challenging. Here, we report a biomimetic study of enantioselective [1,3]-proton shift of *β,β*-difluoro-*α*-imine amides catalyzed by chiral quinine derivatives. A wide range of corresponding *β,β*-difluoro-*α*-amino amides were achieved in good yields with high enantioselectivities. The optically pure *β,β*-difluoro-*α*-amino acid derivatives were further obtained, which have high application values in the synthesis of fluoro peptides, fluoro amino alcohols and other valuable fluorine-containing molecules.

## Abstract (web summary)

Organocatalysed 1,3-proton shifts can offer efficient access to chiral nonracemic fluorinated products. Here chiral amino amides are obtained in high enantiomeric ratio via enantioselective 1,3- proton shift of β,β-difluoro-αimino amides.

## Introduction

Organic fluorides are a class of unique fluorine compounds, which feature with strong electronegativity, similar size to hydrogen, great influence on p*K*_a_ and lipophilicity, and behavior of hydrogen-bond receptors^1–7^. Although fluorine-containing compounds have special physicochemical and biochemical properties, naturally occurring organofluorine compounds are extremely rare^8^. Fluorine earns other extensive applications in modern chemistry. For example, at least 25% of drugs and 50% agrochemicals contain fluorine^9–11^. Chiral-fluorinated amino acids are a class of fluorine-containing building blocks, which are wildely utilized in biorthogonal chemistry, drug modification, and asymmetric catalysis^12–16^. However, the development of chiral fluorine-containing amino acids still has numerous limitations^17^ as below. (i) The preparation of fluorine-containing peptides from natural amino acids is extremely rare, and the application of natural chemical connection is still in its infancy;^18–21^ (ii) compared with aromatic fluorination, the structural diversity of alkyl-fluorinated amino acids needs further study;^22,23^ (iii) the self-disproportionation of enantiomers is also an important factor to prevent the formation of optically pure fluorinated amino acids^24,25^.

**Fig. 1:**
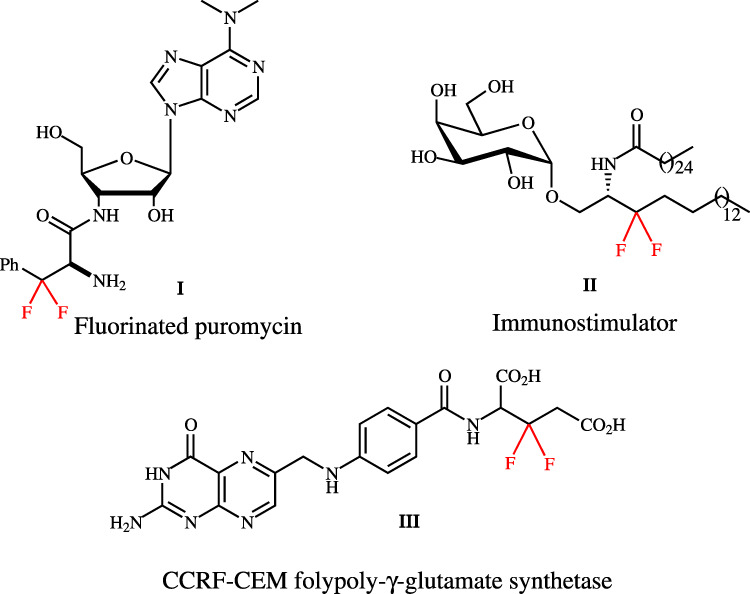
Representative *β,β*-difluoro-*α*-amino acid derivatives. **I**: *β,β*-difluorophenylalanyl puromycin; **II**: 3,3-difluoro-3,4-dideoxy-KRN7000 analog; **III**:CCRF-CEM folypoly-*γ*-glutamate synthetase.

**Fig. 2:**
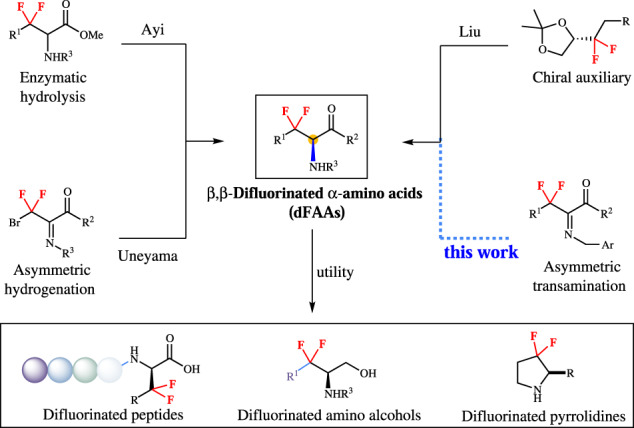
Strategies for asymmetric synthesis of *β,β*-difluoro-*α*-amino acid derivatives. Strategies, **a** enzymatic hydrolysis; **b** asymmetric hydrogenation; **c** chiral auxiliaries. Valuable molecules: difluoro peptides, difluoro amino alcohols, and difluoro alkaloid.

Difluoromethylene has the highest dipole moment in the fluoromethane series and also exerts as bioisostere of ketone and ether, which may change the protein structure^26–29^. Chiral *β,β*-difluoro-*α*-amino acids (dFAAs) are vital building blocks for assembling bioactive molecules^30,31^. As shown in Fig. 1, the *β,β*-difluoro compound **III** acts as a better substrate for human CCRF-CEM folypoly-*γ*-glutamate synthetase;^32^ 3,3-difluoro-3,4-dideoxy-KRN7000 analog **II** is identified as potent immunostimulator;^33^ the *β,β*-difluorophenylalanyl puromycin **I** reacts faster than puromycin at neutral or acidic condition^34^. However, the synthetic methods of chiral dFAAs are not mature, especially in catalytic asymmetric induction. There are only three main routes to prepare chiral dFAAs (Fig. 2). The group of Ayi reported the enzymatic hydrolysis of *β,β*-difluoro-*α*-amino esters *via* kinetic resolution^35^. Liu and his colleagues disclosed an effective method for controlling enantioselectivity by introducing chiral auxiliaries^36–40^. Uneyama uncovered an amazing asymmetric hydrogenation of α-fluoroiminoesters via catalytic amount of chiral BINAP ligand and palladium^41–43^. However, in the current situation, it is urgent to develop a general, efficient, and highly enantioselective dFAAS method under mild conditions.

In the 1990s, Soloshonok et al. first studied the biomimetic [1,3]-proton shift, which opened up a new door for the synthesis of chiral *β*-fluoroalkyl *β*-amino acids^44–46^. Then, Jørgensen^47^, Deng^48–50^, Shi^51,52^, and others^53–55^ further extended this strategy to construct chiral amino acids and chiral trifluoromethylamines. Building upon our continued interests in organic fluorides^56–61^ and the limitations for the rapid synthesis of chiral fluoro amino acids, we report here an approach to achieve dFAAS via biomimetic enantioselective [1,3]-proton-shift reactions. Notably, the yielded chiral dFAAs are useful synthons for producing various valuable molecules, such as difluoro peptides, difluoro amino alcohols, and difluoro alkaloid^62^.

## Results and discussion

### Reaction optimization

We first studied the reaction of *N*-benzyl-2-((2-chlorobenzyl)imino)-3,3-difluoro-5-phenylpentanamide **1a** as model substrate and toluene as solvent. The results are shown in Table 1. When cinchonan-6′, diol **A** was exploited, the expected **2a** was acquired in 34% yield and 81% *ee* (entry 1). Almost racemic **2a** was obtained by replacing the catalyst with quinine **B** (entry 2), while the yield and enantioselectivity were improved by using catalysts **C** and **D** (entries 3, 4). Further tests on catalysts **E** and **F** showed no significant change in yield or *ee* (entries 5, 6). To our delight, when **G** was tested**, 2a** was successfully formed with 87% yield and 95% *ee* and pseudoenantiomer **H** was also tested for delivering the enantiomer of **2a** with 80% yield and 95% *ee* (entries 7 and 8). Solvent evaluation experiments reveal that toluene is the best choice (entries 9–12). Furthermore, reducing the loading of catalyst **G** to 5 mol% (entry 13) without decreasing the yield or *ee* implies that this highly enantioselective biomimetic [1,3]-proton shift can be achieved under suitable conditions. Even if the time is extended to 24 h and the loading is further reduced to 1 mol% (entry 14), the requirement of useless yield can not be met.

**Table 1:** Optimization of asymmetric isomerization of **1a**^a^.


Entry	NHC	Solvent	Yield [%]^b^	*ee* [%]^c^
1	**A**	Toluene	34	81
2	**B**	Toluene	18	2
3	**C**	Toluene	79	93
4	**D**	Toluene	76	95
5	**E**	Toluene	83	90
6	**F**	Toluene	43	95
7	**G**	Toluene	87	95
8	**H**	Toluene	80	95^f^
9	**G**	DCM	81	93
10	**G**	CHCl_3_	85	94
11	**G**	CH_3_CN	51	84
12	**G**	THF	49	77
13^d^	**G**	Toluene	86	95
14^e^	**G**	Toluene	27	96

^a^Conditions: **1a** (0.05 mmol), catalyst (10 mol%), solvent (0.5 mL), room temperature, 2 h.

^b^Isolated yield after flash column chromatography.

^c^Enantiomeric excess (*ee*) determined via chiral-phase HPLC analysis.

^d^Cat. **G** (5 mol%) was used; 4 h.

^e^Cat. **G** (1 mol%) was used; 24 h.

^f^Enantiomer was obtained.

### Substrate scope

**Fig. 3:**
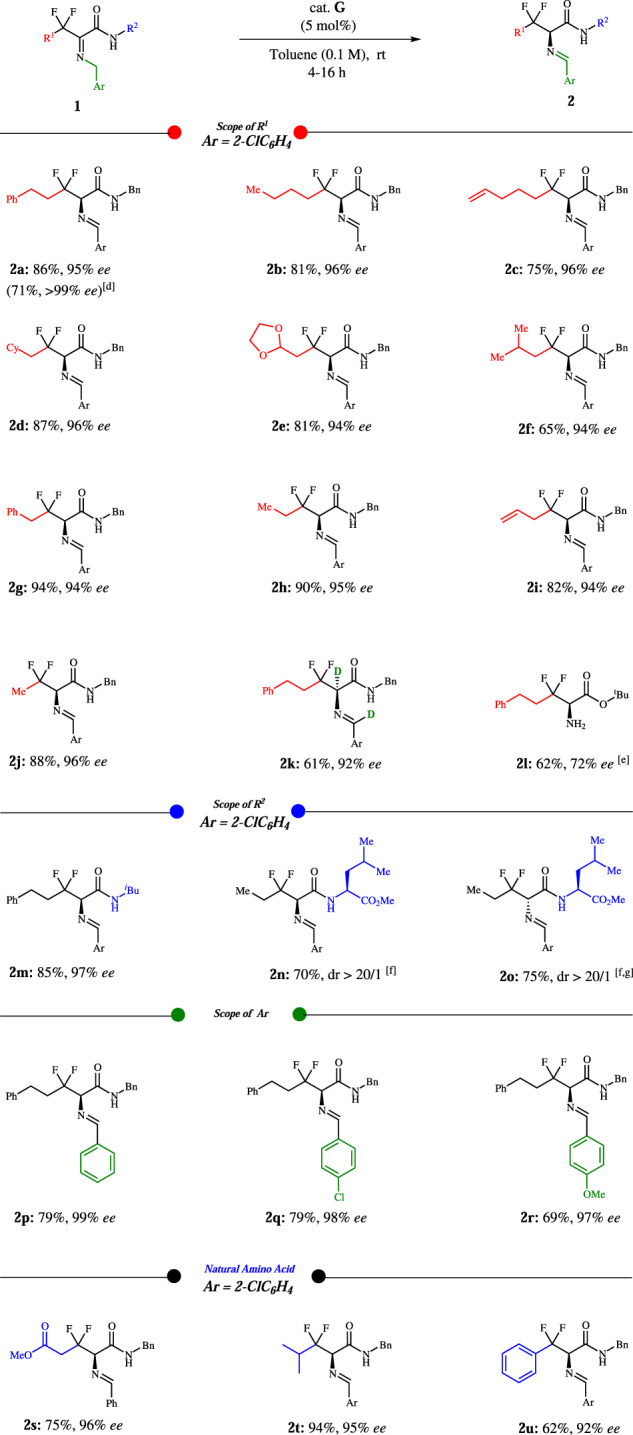
Scope of *β,β*-difluorinated imines^[a,b,c]^. **a** Conditions: **1a** (0.1 mmol), cat. **G** (5 mol%), toluene (1.0 mL), room temperature, 4–16 h. **b** Isolated yield after flash-column chromatography. **c** Enantiomeric excess (*ee*) determined via chiral-phase HPLC analysis. **d** Recrystallization from hexane/ethyl acetate (20/1). **e** After hydrolysis. **f** dr was determined via crude ^1^H NMR. **g** Cat. **H** (5 mol%) was used.

Under the optimized catalytic conditions, we turned our attention to explore the generality of biomimetic [1,3]-proton-shift reaction. As illustrated in Fig. 3, R^1^ group was examined first. In the process of generating the [1,3]-proton-shift products **2a** and **2b**, it was found that the yield and enantioselectivity are independent of the adjacent CH_2_CH_2_ groups of the substituents (**1a** and **1b**). Alkene (**1c**) is also compatible under this condition. Furthermore, the CH_2_ adjoint groups containing cyclohexyl, 1,3-dioxolan-2-yl, isopropyl, phenyl, methyl and alkene were well tolerated and provided good yields and high enantioselectivities (**2d–2i**). *β,β*-difluoro-2-aminobutyric amide (**2j**) was obtained with good yield and excellent *ee*. In the case of **2** **l** that is a hydrolyzed derivative and bearing ester group, 72% ee and 62% yield were observed. Then, we turned to amide group R^2^, where isobutyl can be installed smoothly (**2** **m**). It is gratifying that dipeptides (**2n** and **2o**) bearing leukine have been successfully provided for peptide synthesis and the diastereoisomers indicated that the source of chirality is induced by the catalyst rather than substrate itself. Pleasingly, the imines with or without substituted phenyl group are well tolerated under these conditions (**2p–2r**). *β,β*-difluoro natural amino acid derivatives (e.g., *β,β*-difluoro glutamine, *β,β*-difluoro leucine, and *β,β*-difluoro phenylalanine) were all obtained in good yields and high *ee*’s.

### Mechanistic studies and postulated mechanism

**Fig. 4:**
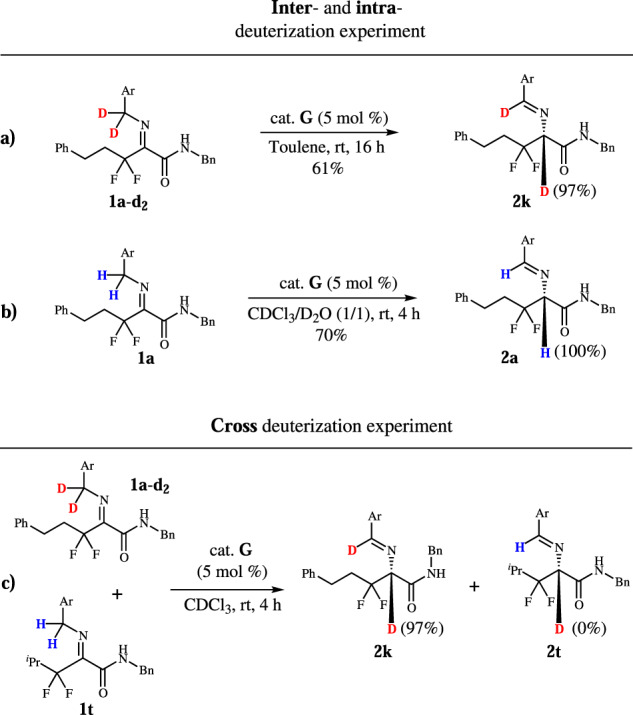
Mechanistic studies. Deuterization and cross deuterization experiment.

Next, we started to investigate the reaction mechanism. We want to understand whether the [1,3]-proton-shift process is intermolecular or intramolecular. Benzyl-deuterated **1a-d**_**2**_ was prepared and the reaction carried out under standard conditions. The results show that **2k** has no erosion on deuterated ratio (Fig. 4a). As a contrast, when deuterium oxide was added to the reaction system, no deuterium product was found (Fig. 4b).

**Fig. 5:**
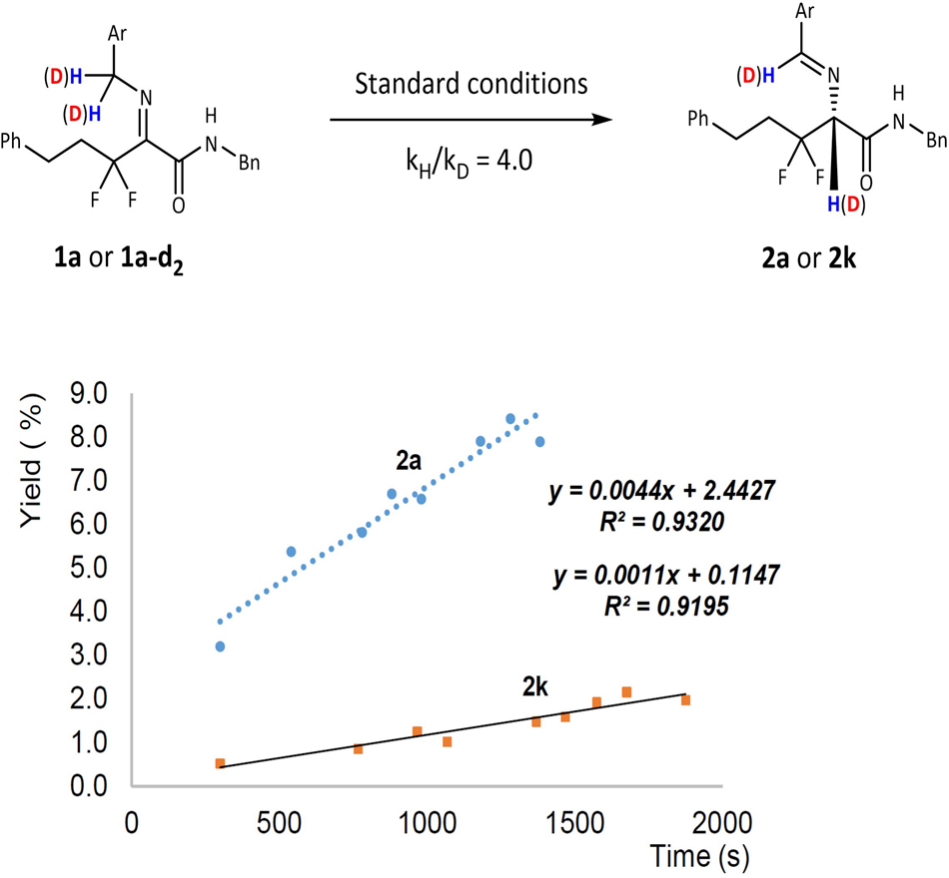
KIE from parallel experiments. The parallel experiments of **1a** and **1a-d**_**2**_.

When **1a-d**_**2**_ cross-reacted with **1t**, no deuterium was found in product **2t** (Fig. 4c). These experiments reveal that the [1,3]-proton-shift process is intramolecular. The parallel kinetic isotope effects (KIE) were measured with k_H_/k_D_ of 4.0, indicating that the rate-determining step (RDS) is the process of hydrogen leaving from benzyl group (Fig. 5).

**Fig. 6:**
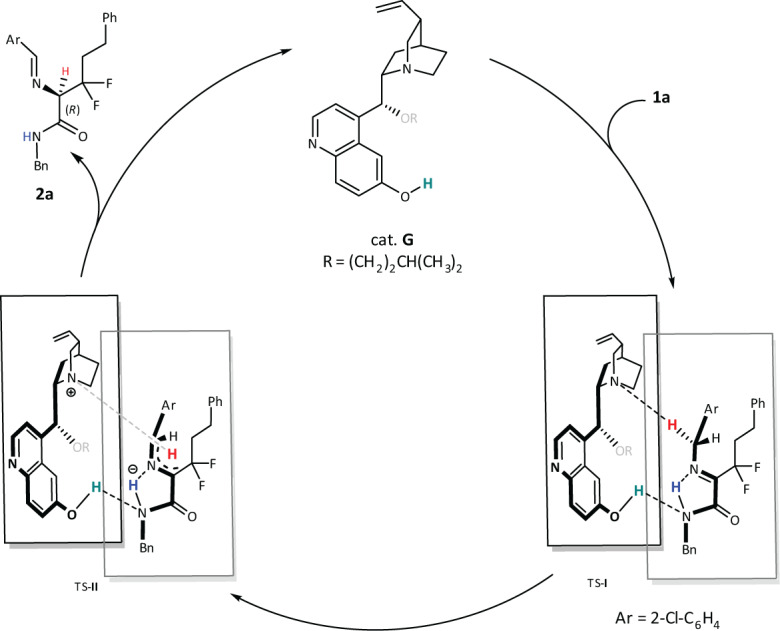
Proposed transition model. The possible mechanistic of the enantioselective [1,3]-proton-shift reaction.

Based on the condition optimization, substrate scope, and the initial mechanistic experiments, a plausible mechanism for the biomimetic enantioselective [1,3]-proton-shift reaction was proposed (Fig. 6). First, the possible intramolecular hydrogen-bond interaction (blue) of the amide NH to imine of the substrate is essential, and without it, the *ee* decreased dramatically (e.g., **2** **l** bearing an ester group resulted in 72% *ee*). Then, the catalyst with free OH of phenol is needful (e.g., cat. **B** with OMe only induced 2% *ee*) for assembling another hydrogen bond between it and the N of the amide (green). The bulk isopentyloxy of the catalyst enforced deprotonation of the inner H of benzyl (red) and this process was a RDS (TS-**I**). Finally, the asymmetric protonation of 2-azaallyl anion from *Si* face (TS-**II**) to deliver the target product with *R*-configuration.

### Synthetic transformations and applications

**Fig. 7:**
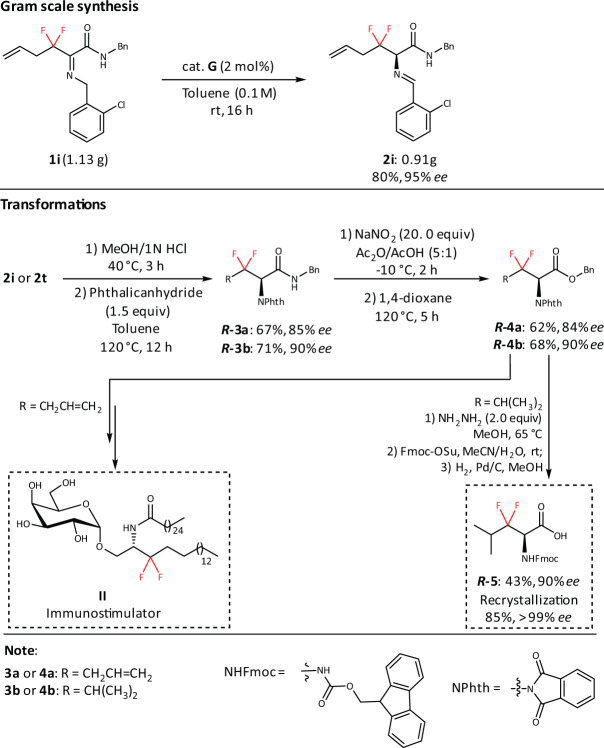
Gram-scale synthesis and transformations. The gram-scale synthesis of **1i**. The transformation of **2i** to **II** and **2t** to **5**.

**Fig. 8:**
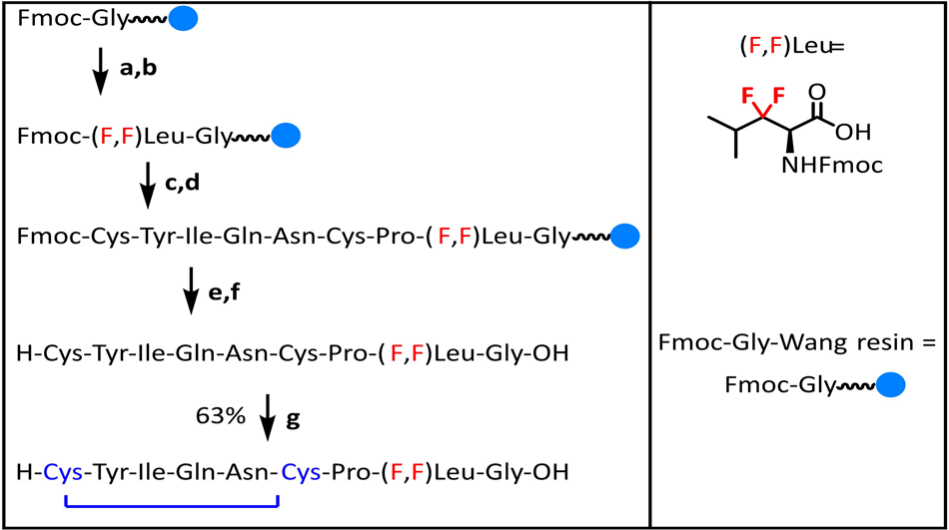
Solid-phase peptide synthesis of difluorinated oxytocin. **a** About 20% piperidine/DMF, 10 min, double; **b** 1.5 equiv. fluorinated leucine, 1.5 equiv. HOBt, 1.5 equiv. DIC, minimum DMF, rt, 16 h; **c** 10% piperidine/DMF, 3 mins, double; **d** 3 equiv. amino acid, 2.9 equiv. HBTU, 6 equiv. DIPEA, minimum DMF, rt, 1.5 h; **e** 10% piperidine/DMF, 3 mins, double; **f** TFA cocktail, global deprotection, rt, 3 h; **g** GSH/GSSG folding, rt, over-night. Fmoc = fluorenylmethoxycarbonyl, HOBt = hydroxybenzotriazole, DIC = *N,N’*-diisopropylcarbodiimide, HBTU = *N,N,N’,N’*-tetramethyl-O-(1H-benzotriazol-1-yl)-uronium hexafluorophosphate, DIPEA = *N,N*-diisopropylethylamine, GSSG = glutathione disulfide.

**Fig. 9:**
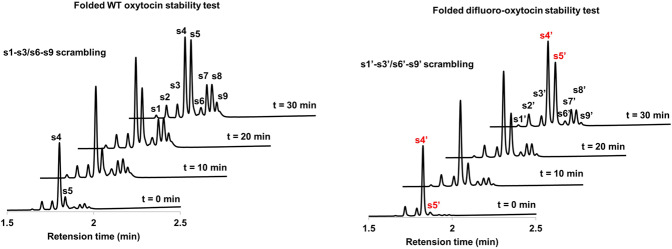
Folded difluoro-oxytocin stable than folded WT oxytocin. s4: fold WT-oxytocin, s5: linear WT oxytocin; s4′: fold difluoro-oxytocin, s5′: linear difluoro-oxytocin, s1–s3/s6–s9 scrambling, s1′–s3′/s6′–s9′ scrambling.

In addition, our protocol is potentially suitable for large-scale preparation. As illustrated in Fig. 7, the use of 2 mol% catalyst **G** is sufficient to produce **2i** (0.91 g) in 80% yield and 95% *ee*. Compound **3** can be achieved via hydrolysis and *N*-Phth protection. Next, the conversion of amide to ester group can generate the key intermediate **4**^63^. Finally, 3,3-difluoro-3,4-dideoxy-KRN7000 analog **II** can be made smoothly from **4a** based on formal synthesis^33^. Meanwhile, the corresponding Fmoc-protected amino acid **5** can be prepared from **4b** for further medicinal study. To further prove the practicability of dFAAs, difluoro leukine was assembled into linear difluoro-oxytocin (28% yield for overall steps), which could be folded smoothly by using glutathione oxidation (63% yield) (Fig. 8). Additionally, we demonstrated that the stability of folded difluoro-oxytocin (3 mM GSH, 100 mM PBS, pH 7.0, rt) is higher than WT oxytocin^64–67^(Fig. 9).

In summary, a biomimetic enantioselective [1,3]-proton shift of difluoroimines has been developed. This new protocol allows the rapid assembly of enantiomerically enriched dFAAs from readily available starting materials under mild conditions. Preliminary mechanism studies show that the proton transfer is intramolecular and the deprotonation step is RDS. More details on enantiocontrol are being conducted in our laboratory and will be reported in due course.

## Methods

### Synthesis of 2

To a flame-dried Schlenk reaction tube equipped with a magnetic stir bar, was added the **1** (0.1 mmol) and cat. **G** (1.9 mg, 0.005 mmol). The Schlenk tube was closed with a septum, and toluene (1.0 mL) was added. The mixture was then stirred at 25 °C and monitored by TLC until **1** was consumed. The mixture was concentrated under reduced pressure and purified by column chromatography on silica gel (hexane/EtOAc = 20:1–10:1) to afford the desired product **2**. Full experimental details can be found in the Supplementary Methods.

## Supplementary information

Supplementary Information

Supplementary Data 1

Description of Additional Supplementary Files

## Data Availability

For ^1^H NMR, ^13^C NMR, and ^19^F NMR spectra see Supplementary Figs. 1–75 and highperformance liquid-chromatography spectra see Supplementary Figs. 76–118.The X-ray crystallographic coordinates for **Fmoc-2l** (Supplementary Data 1) reported in this article have been deposited at the Cambridge Crystallographic Data Centre (CCDC), under deposition number CCDC 2102965. These data can be obtained free of charge from The Cambridge Crystallographic Data Centre via www.ccdc.cam.ac.uk/data_request/cif.
