# Supplementary material for: Biomimetic enantioselective synthesis of β,β-difluoro-α-amino acid derivatives
Source: Commun Chem. 2021 Oct 22;4:148. doi: 10.1038/s42004-021-00586-z (PMC9814941; doi:10.1038/s42004-021-00586-z)
Supplement: Supplementary file 1 — Supplementary Information [file 42004_2021_586_MOESM1_ESM.pdf]

Supporting Information

Contents:

NMR Spectra 2

HPLC Spectra 52

Supplementary Methods 74

General Information 74

Procedure for Synthesis of Catalyst **G** 75

General Procedure for Synthesis of Fluorinated imines 76

General Procedure for Catalytic Reaction of Fluorinated imines 76

Sensitivity Assessment 77

Characterization Data 77

Gram Scale Synthesis Procedure 86

Synthetic Transformation 87

Crystal Structure of **Fmoc-2l** 88

General methods for manual peptides synthesis 89

The stability tests of fold Difluoro-oxytocin and its corresponding WT Oxytocin in GSH 92

Supplementary References 94

NMR Spectra

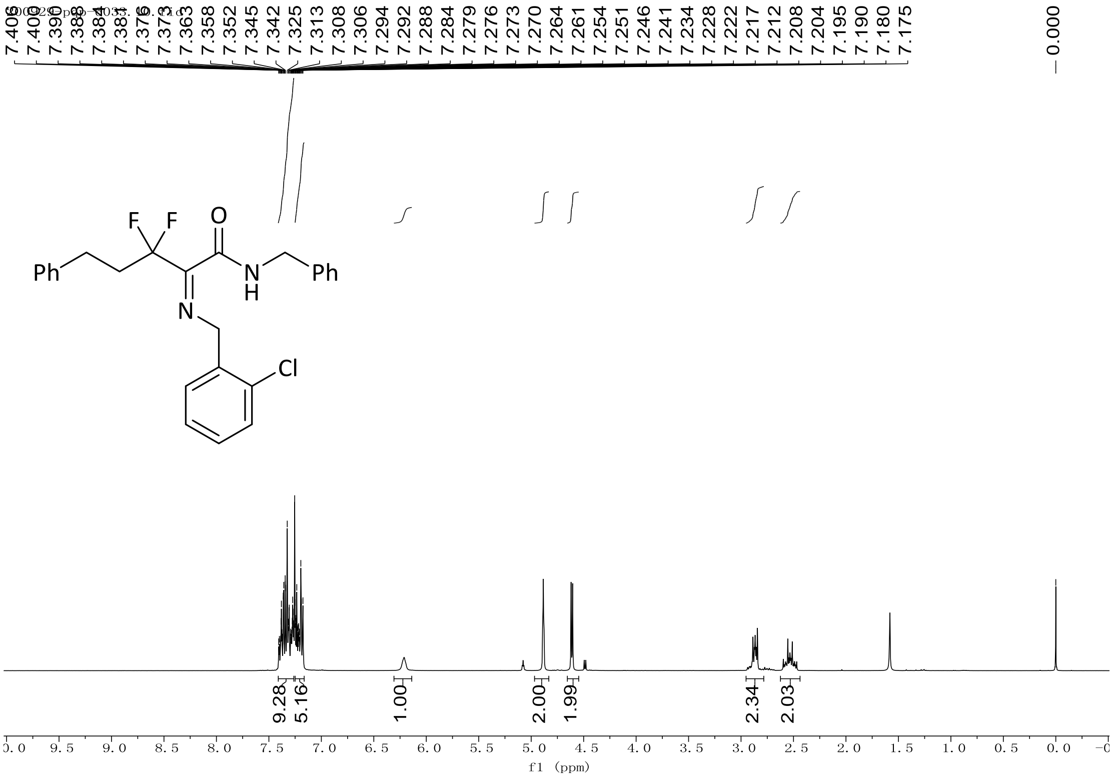

Supplementary Figure 1. ^{1}H NMR Spectra of **1a**

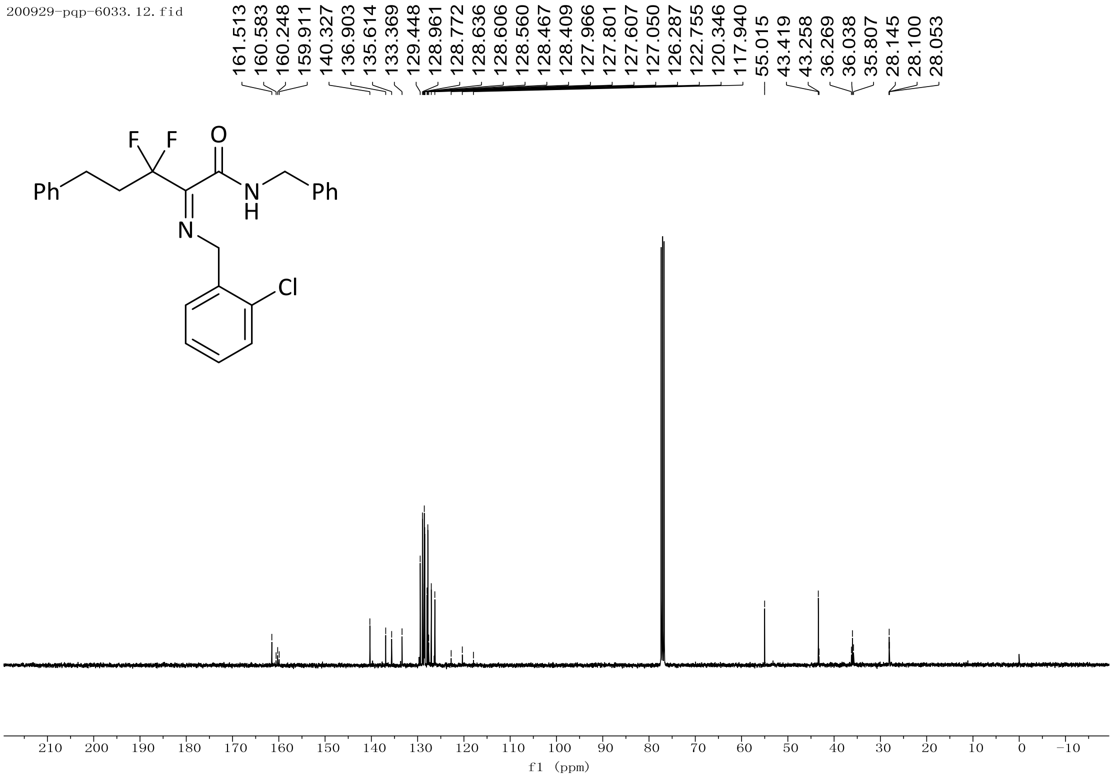

Supplementary Figure 2. ^{13}C NMR Spectra of **1a**

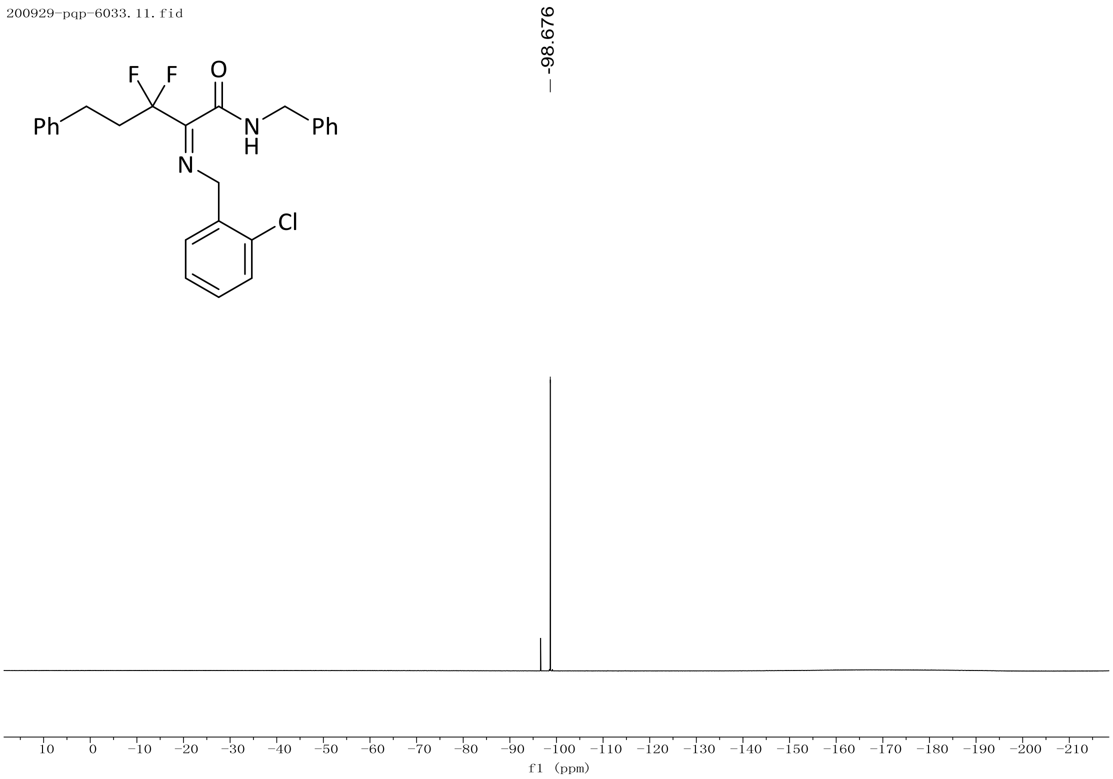

Supplementary Figure 3. ^{19}F NMR Spectra of **1a**

2a

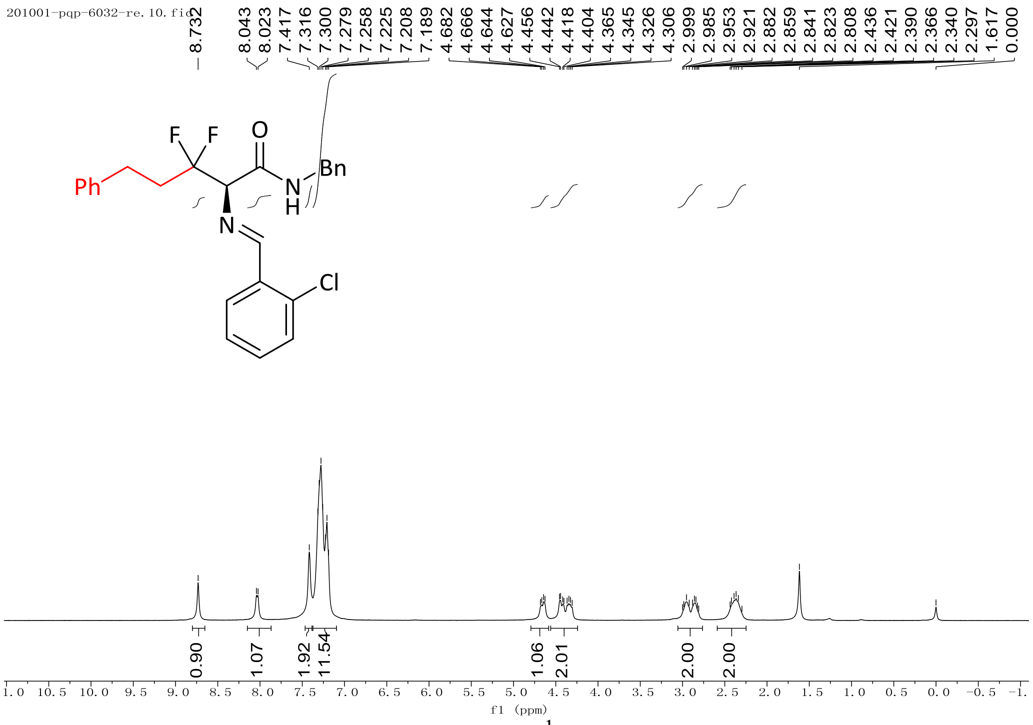

Supplementary Figure 4. ^{1}H NMR Spectra of **2a**

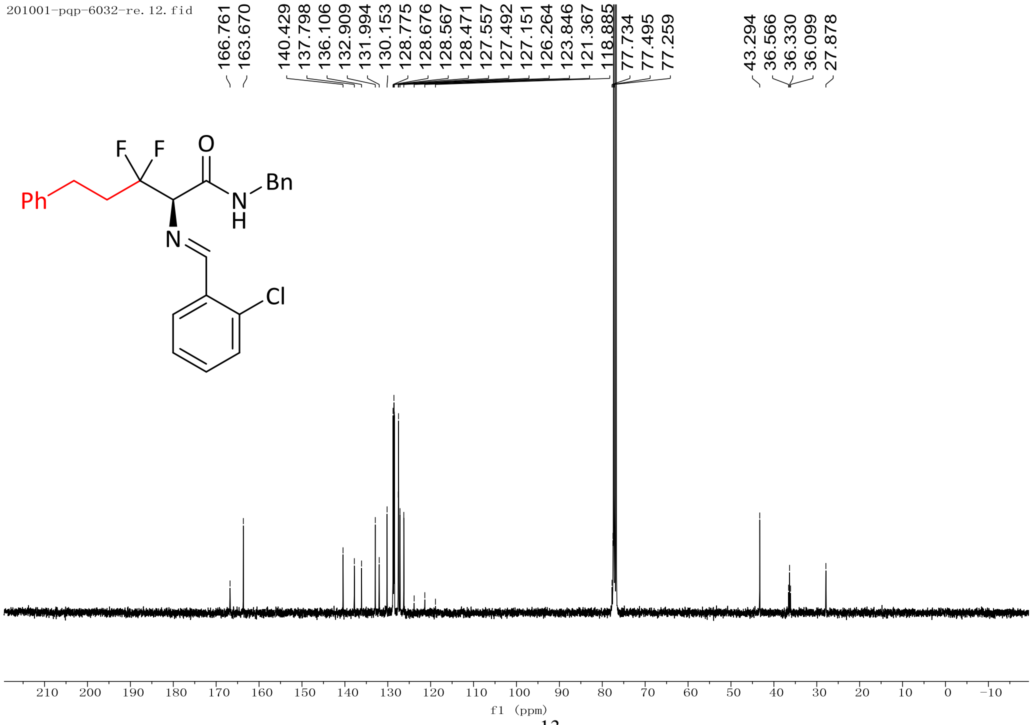

Supplementary Figure 5. ^{13}C NMR Spectra of **2a**

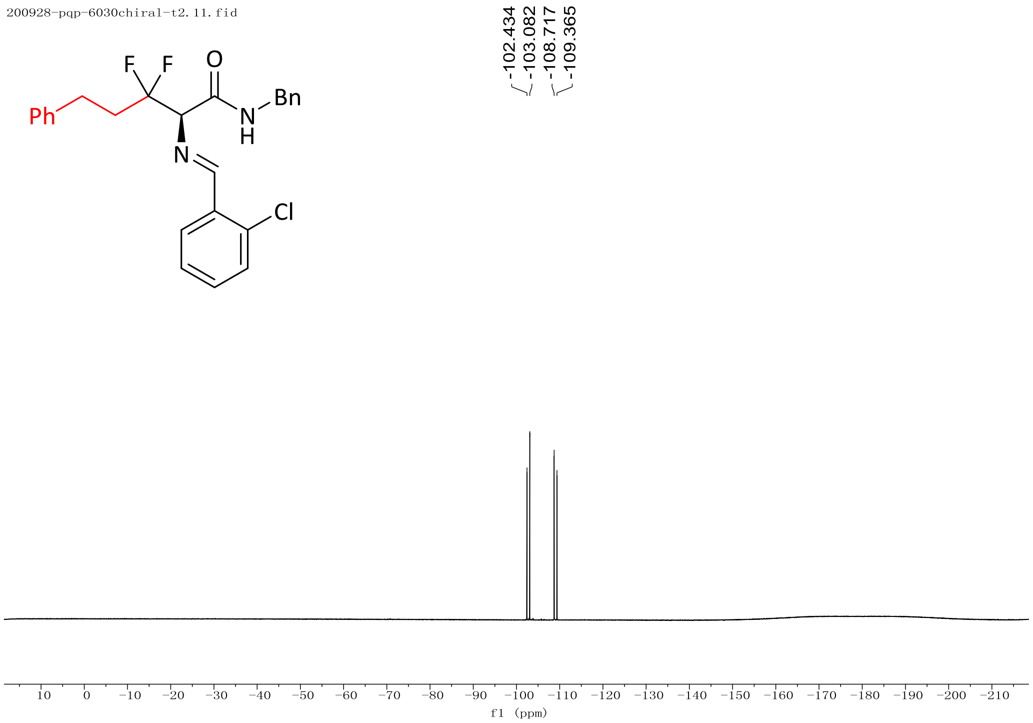

Supplementary Figure 6. ^{19}F NMR Spectra of **2a**

2b

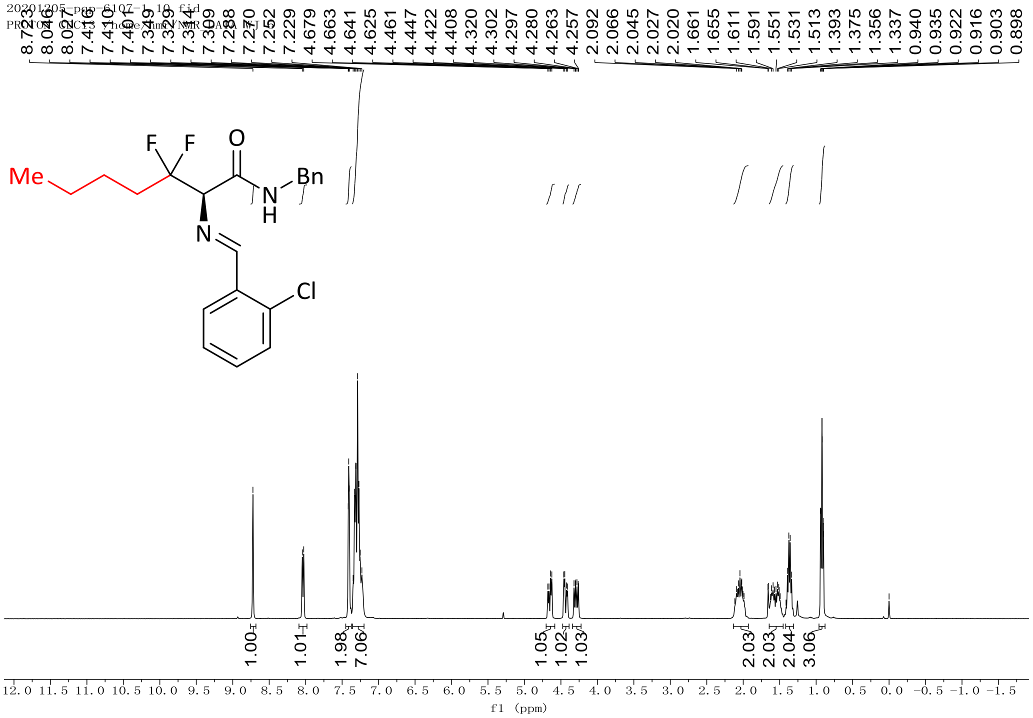

Supplementary Figure 7. ^{1}H NMR Spectra of **2b**

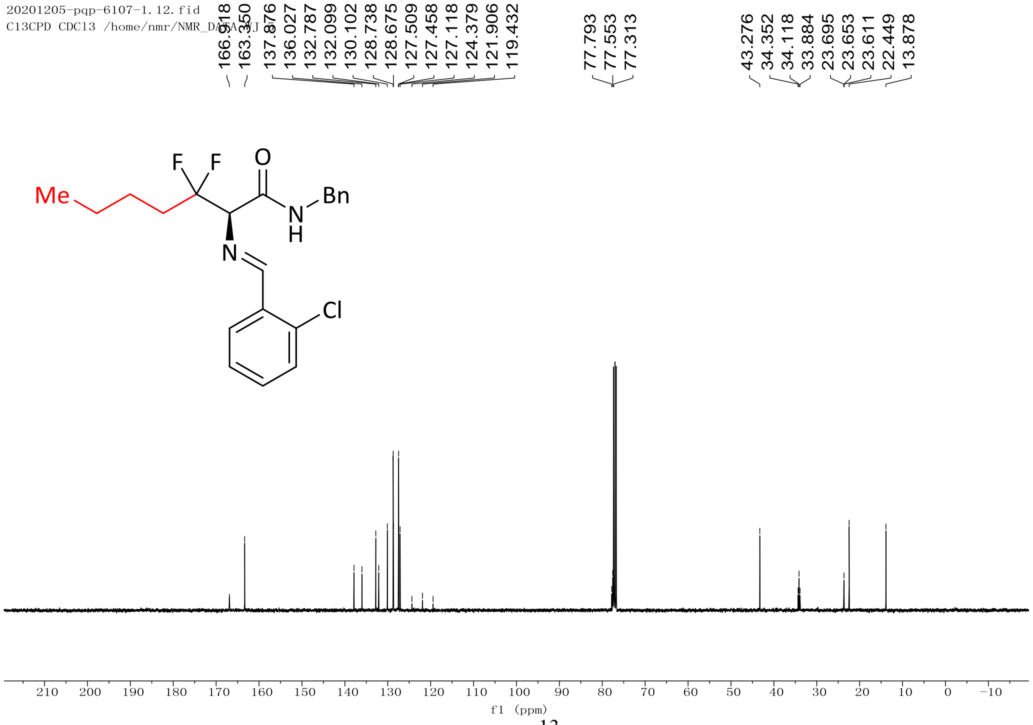

Supplementary Figure 8. ^{13}C NMR Spectra of **2b**

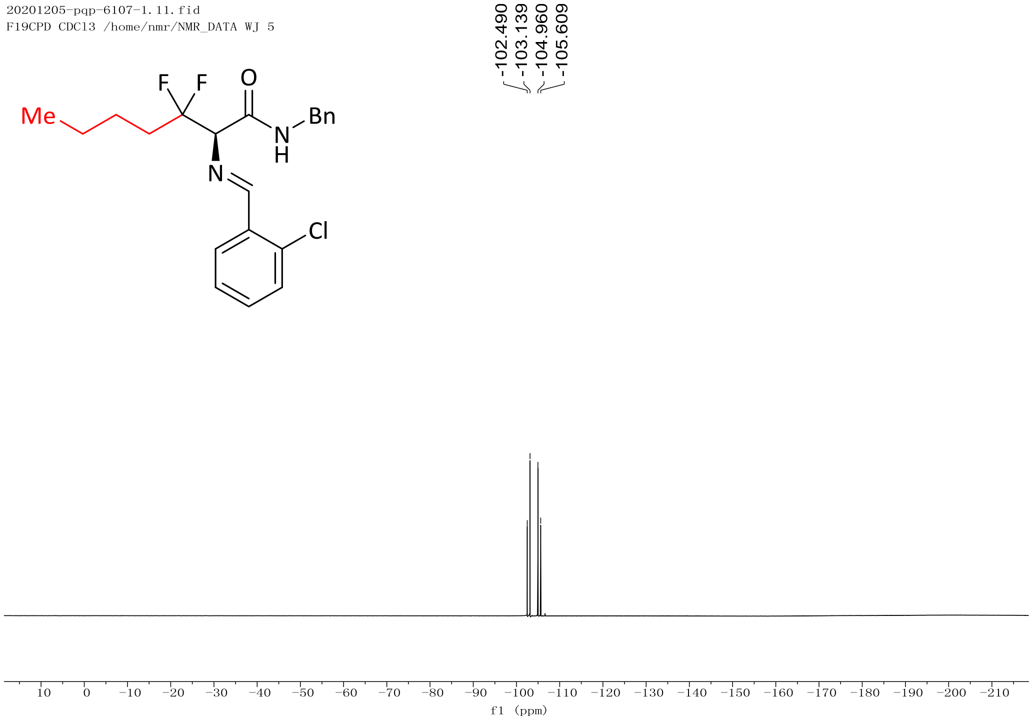

Supplementary Figure 9. ^{19}F NMR Spectra of **2b**

2c

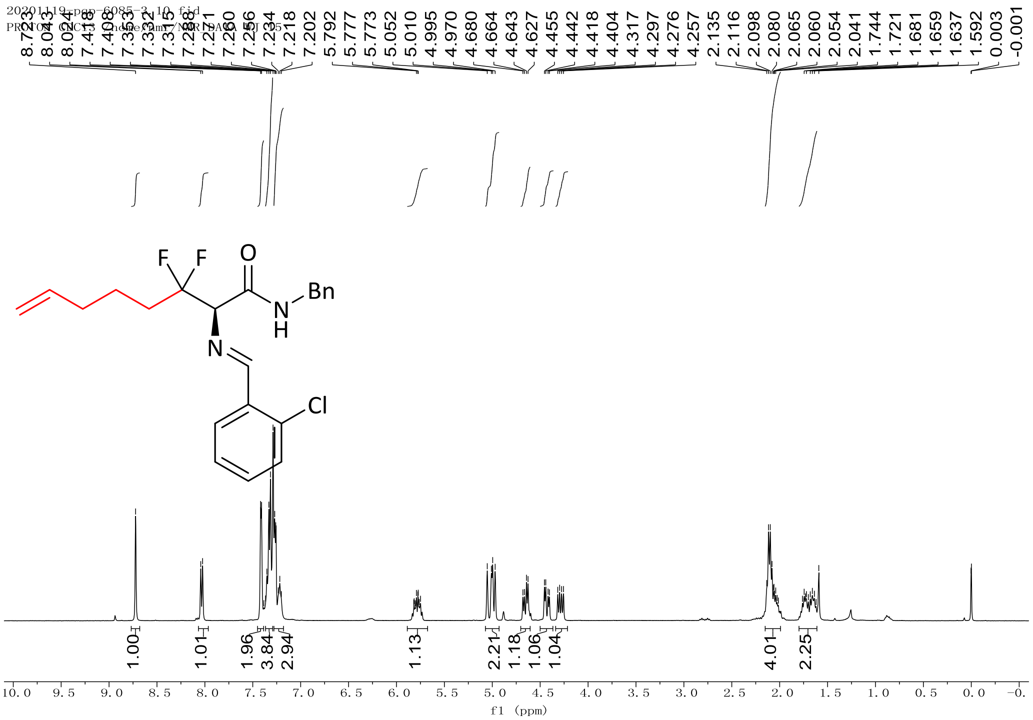

Supplementary Figure 10. ^{1}H NMR Spectra of **2c**

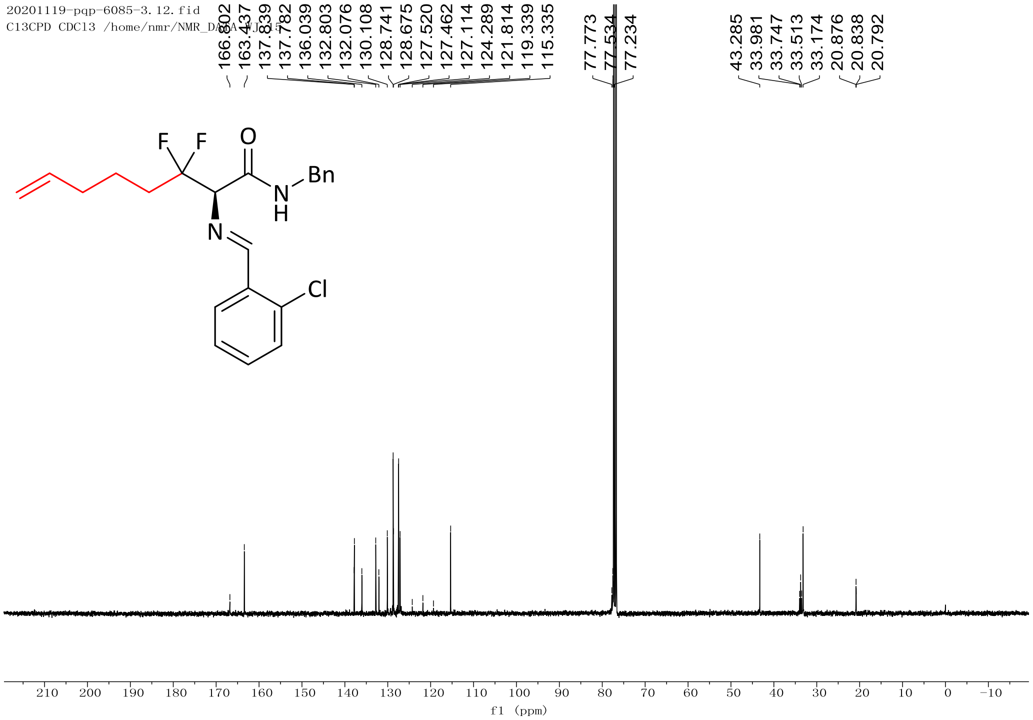

Supplementary Figure 11. ^{13}C NMR Spectra of **2c**

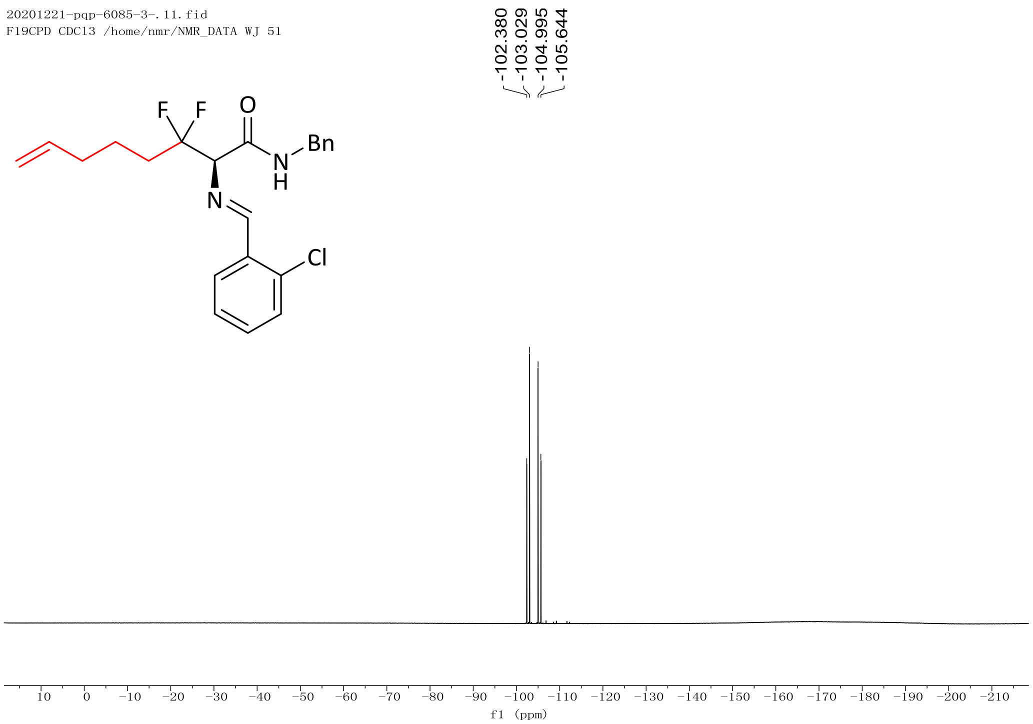

Supplementary Figure 12. ^{19}F NMR Spectra of **2c**

2d

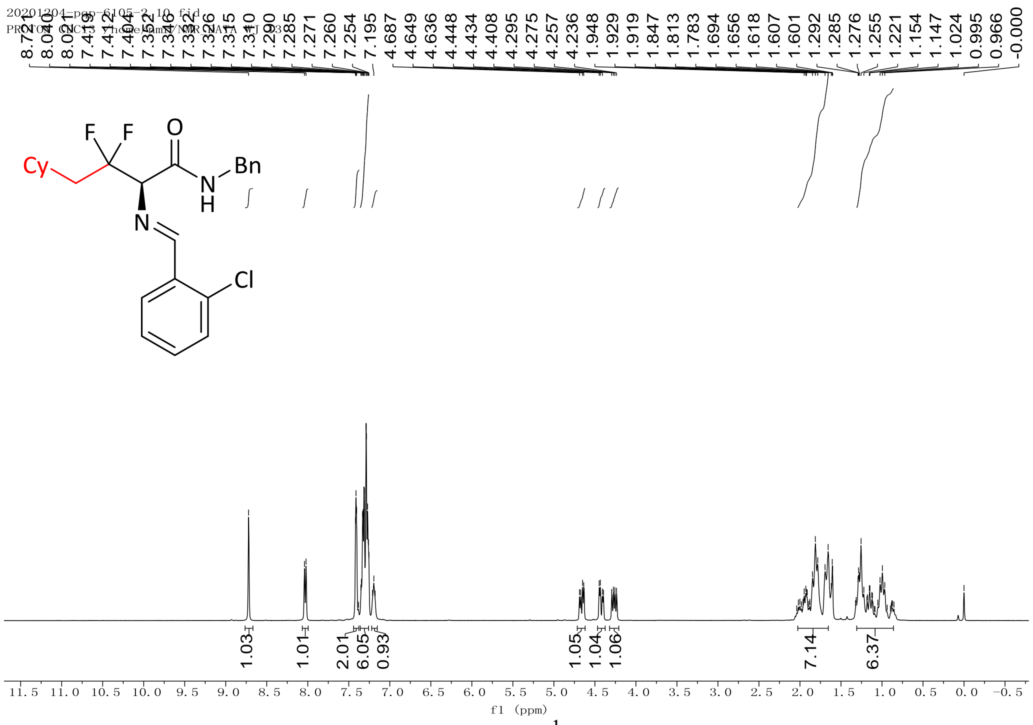

Supplementary Figure 13. ^{1}H NMR Spectra of **2d**

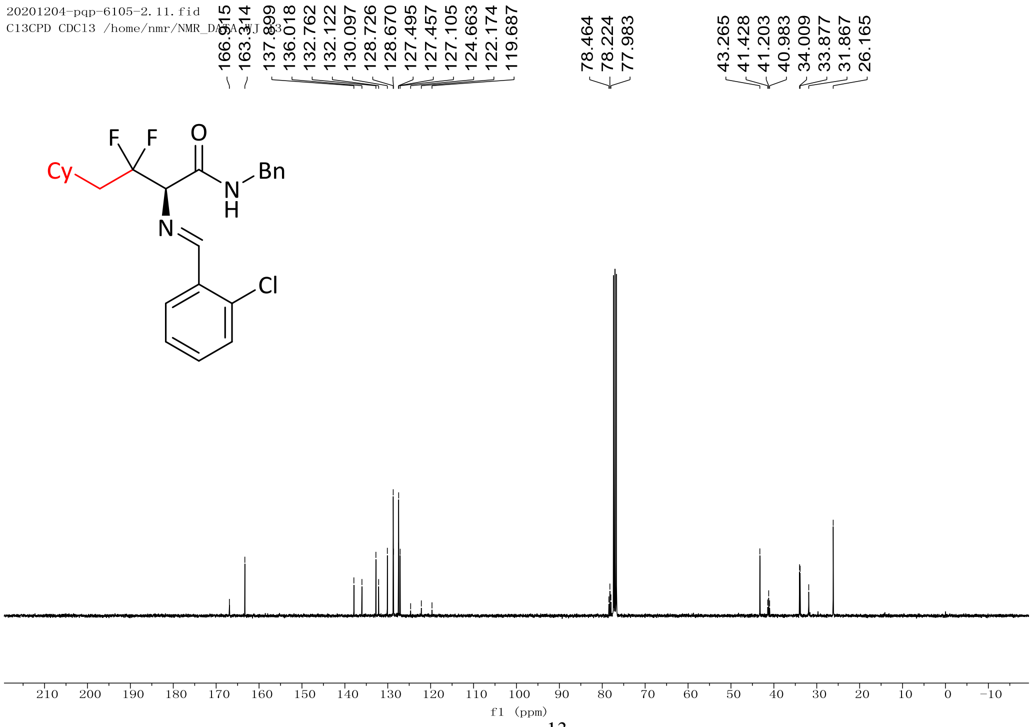

Supplementary Figure 14. ^{13}C NMR Spectra of **2d**

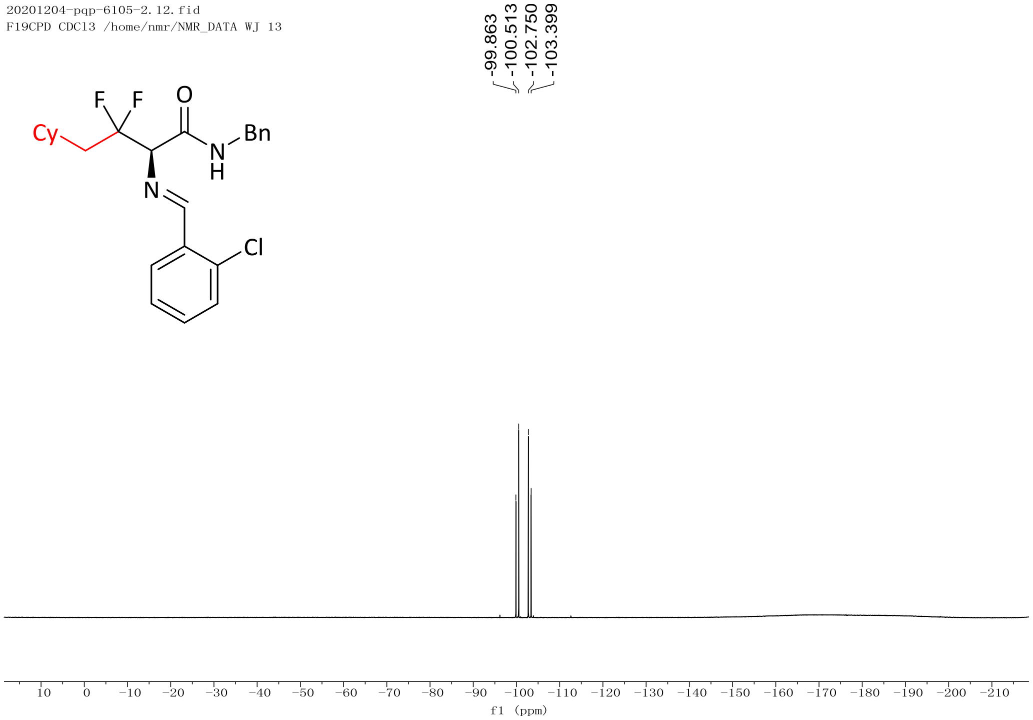

Supplementary Figure 15. ^{19}F NMR Spectra of **2d**

2e

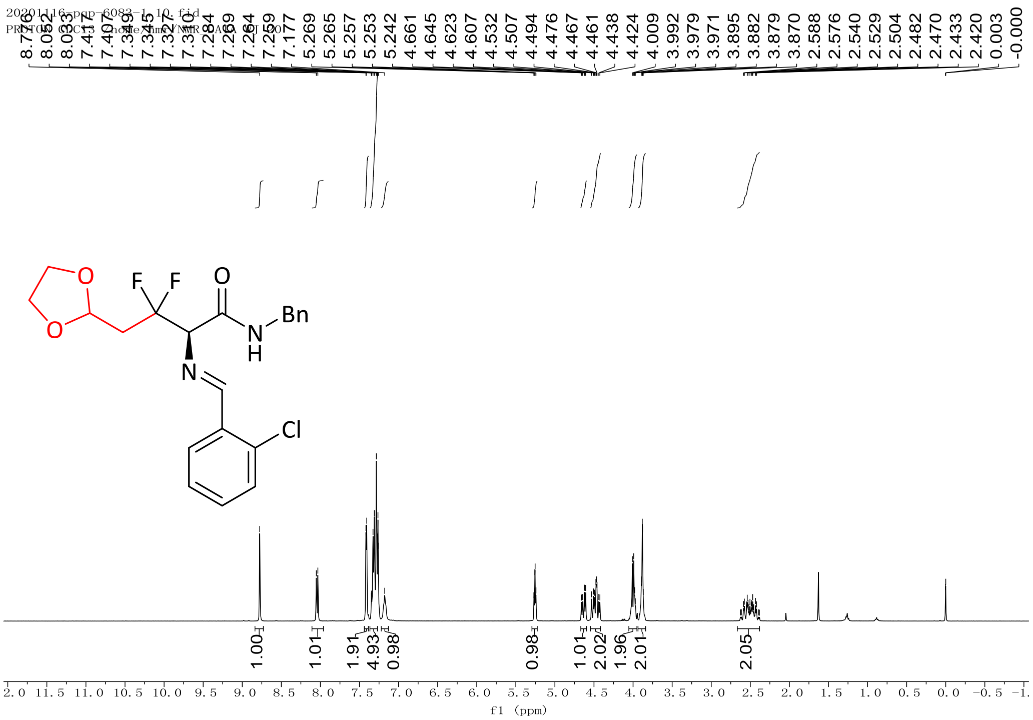

Supplementary Figure 16. ^{1}H NMR Spectra of **2e**

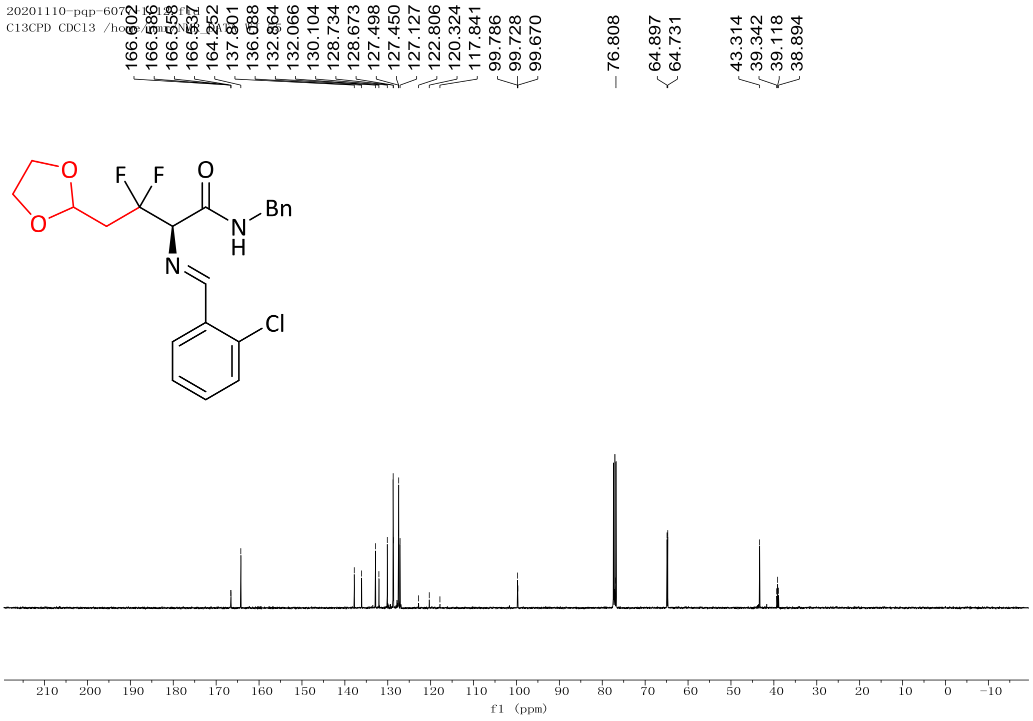

Supplementary Figure 17. ^{13}C NMR Spectra of **2e**

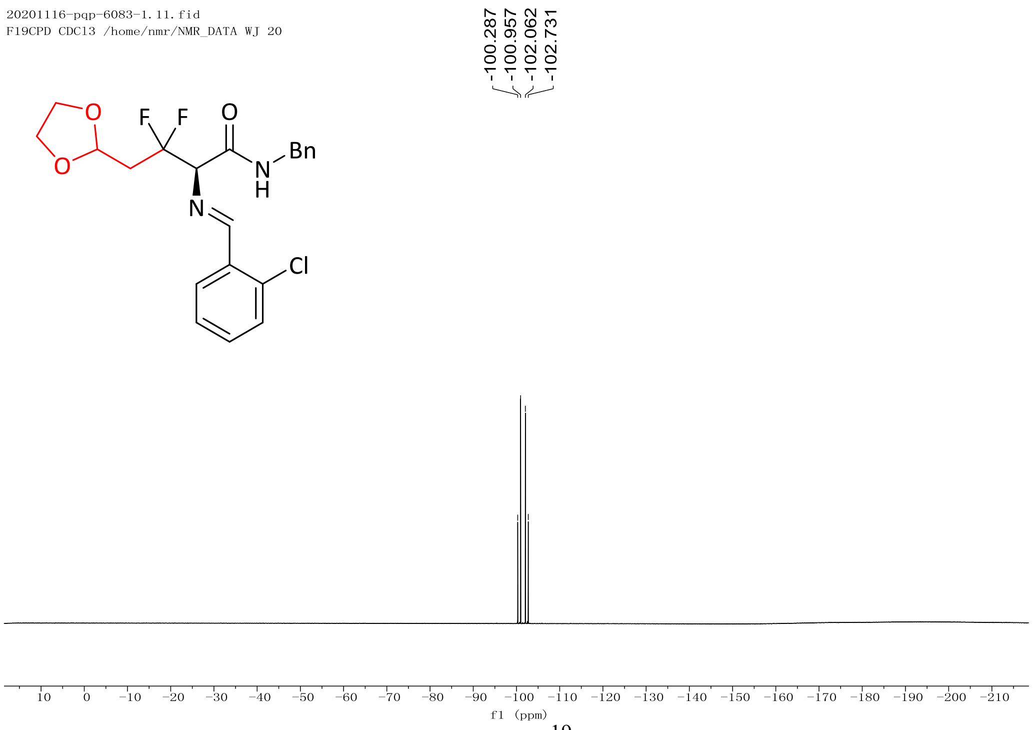

Supplementary Figure 18. ^{19}F NMR Spectra of **2e**

2f

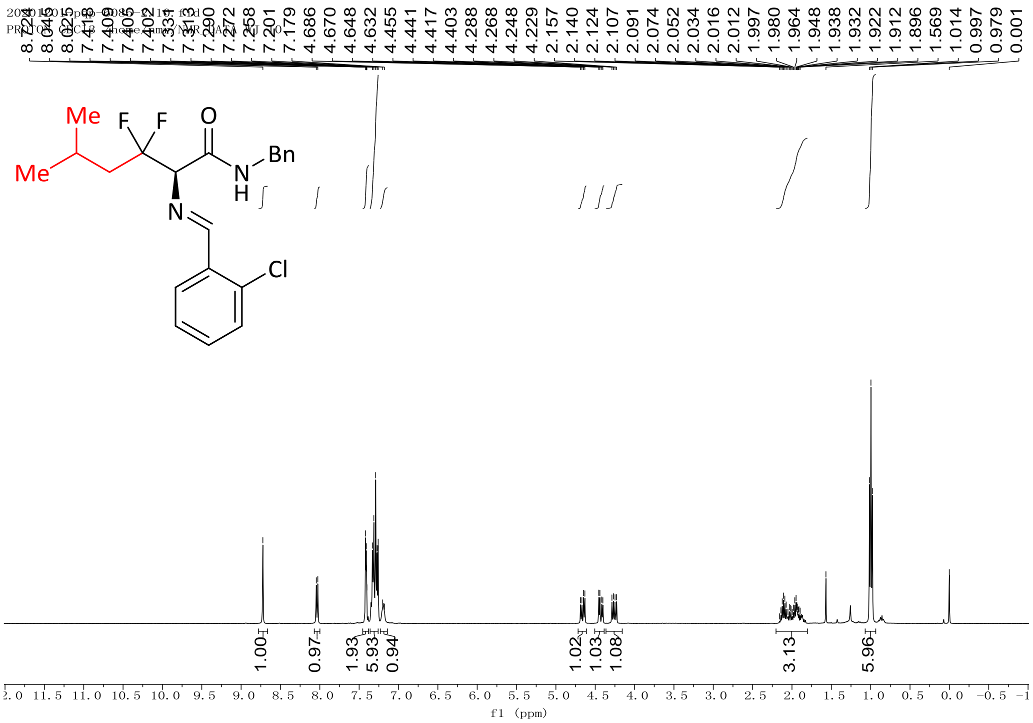

Supplementary Figure 19. ^{1}H NMR Spectra of **2f**

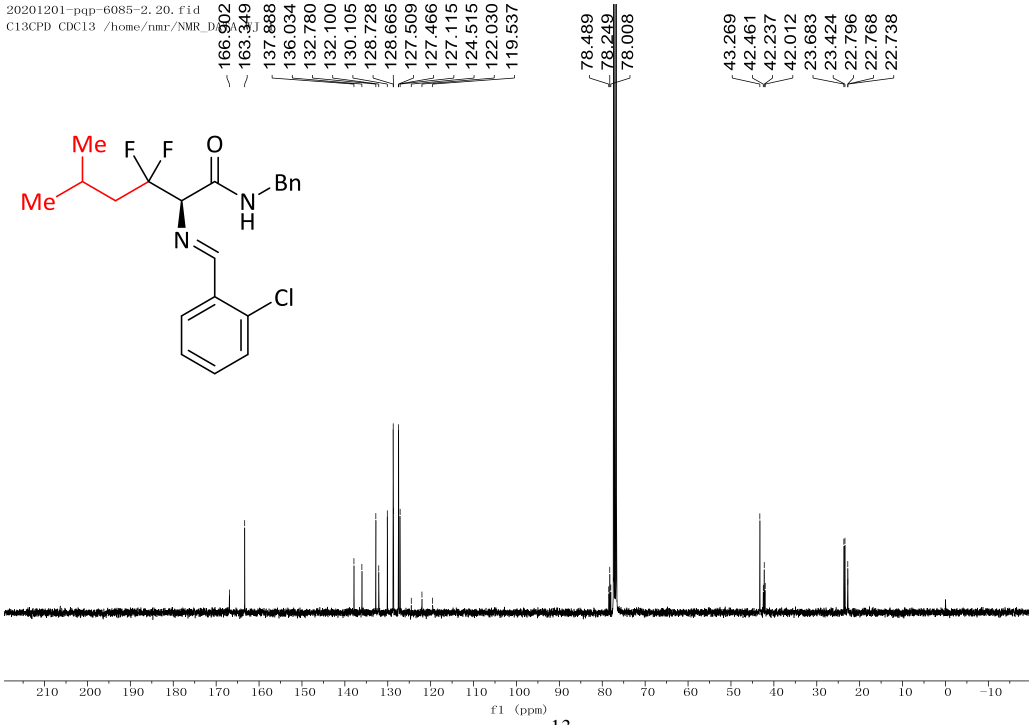

Supplementary Figure 20. ^{13}C NMR Spectra of **2f**

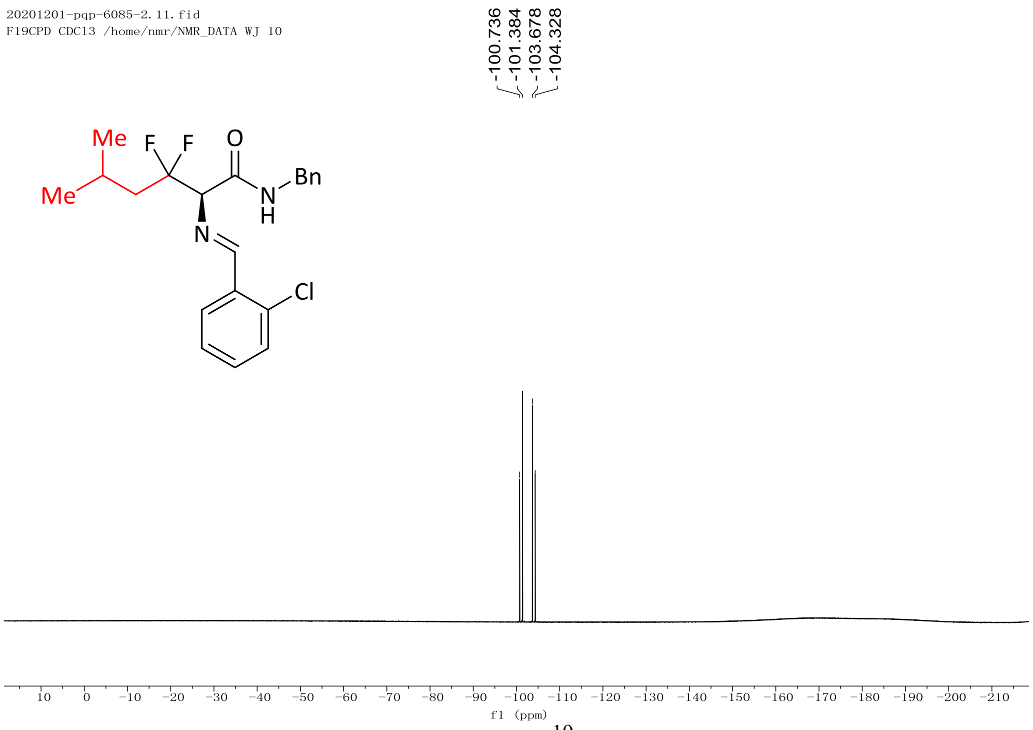

Supplementary Figure 21. ^{19}F NMR Spectra of **2f**

2g

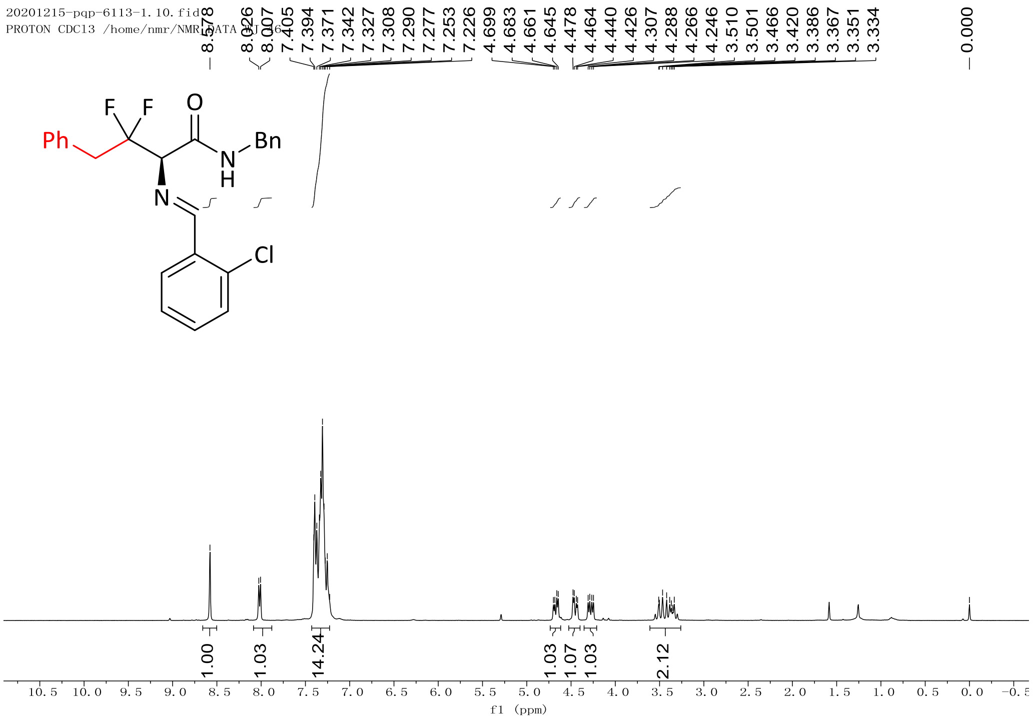

Supplementary Figure 22. ^{1}H NMR Spectra of **2g**

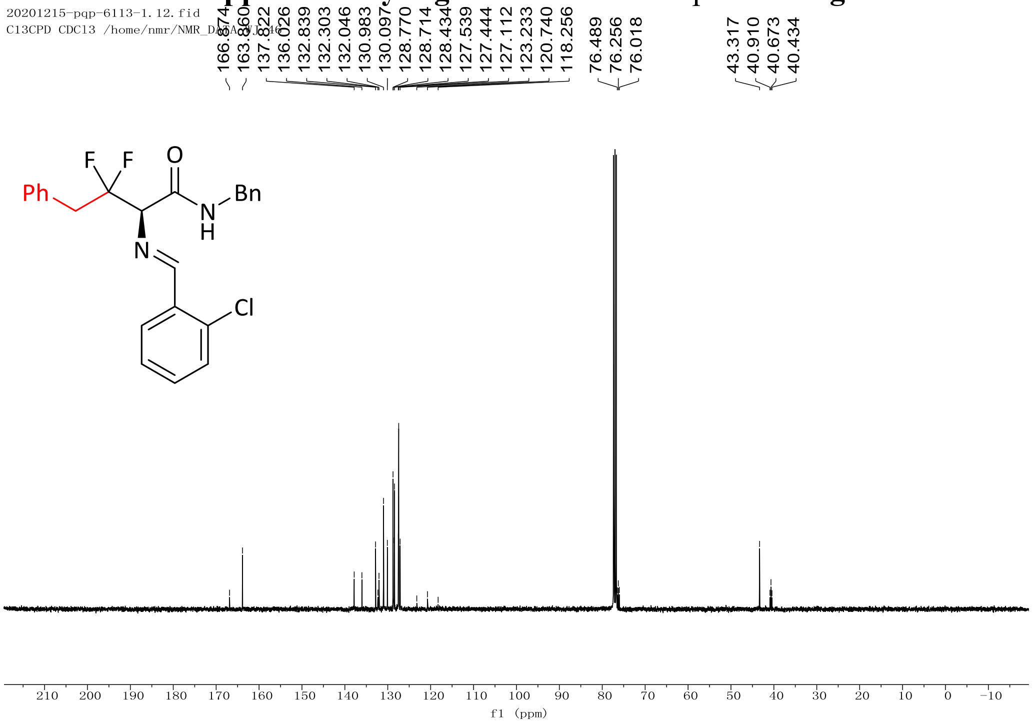

Supplementary Figure 23. ^{13}C NMR Spectra of **2g**

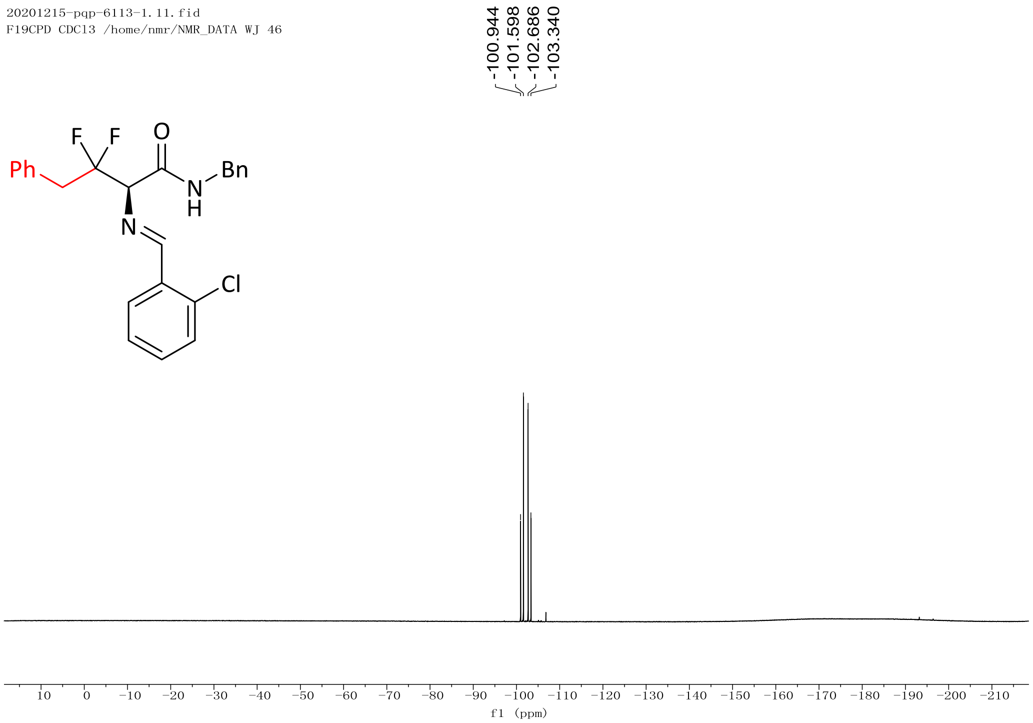

Supplementary Figure 24. ^{19}F NMR Spectra of **2g**

2h

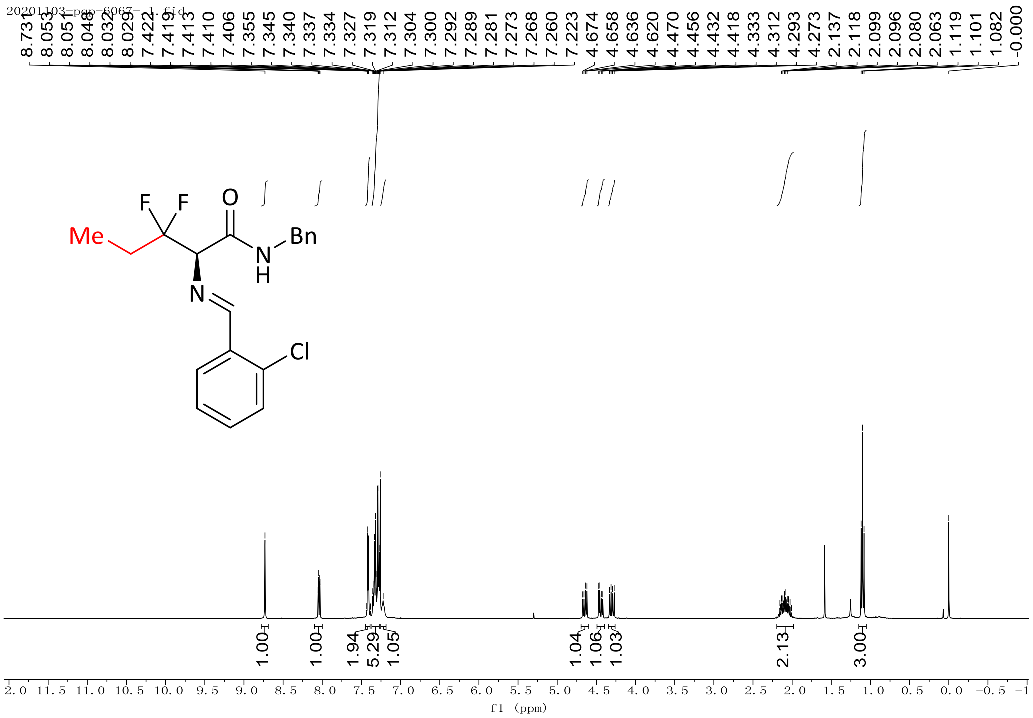

Supplementary Figure 25. ^{1}H NMR Spectra of **2h**

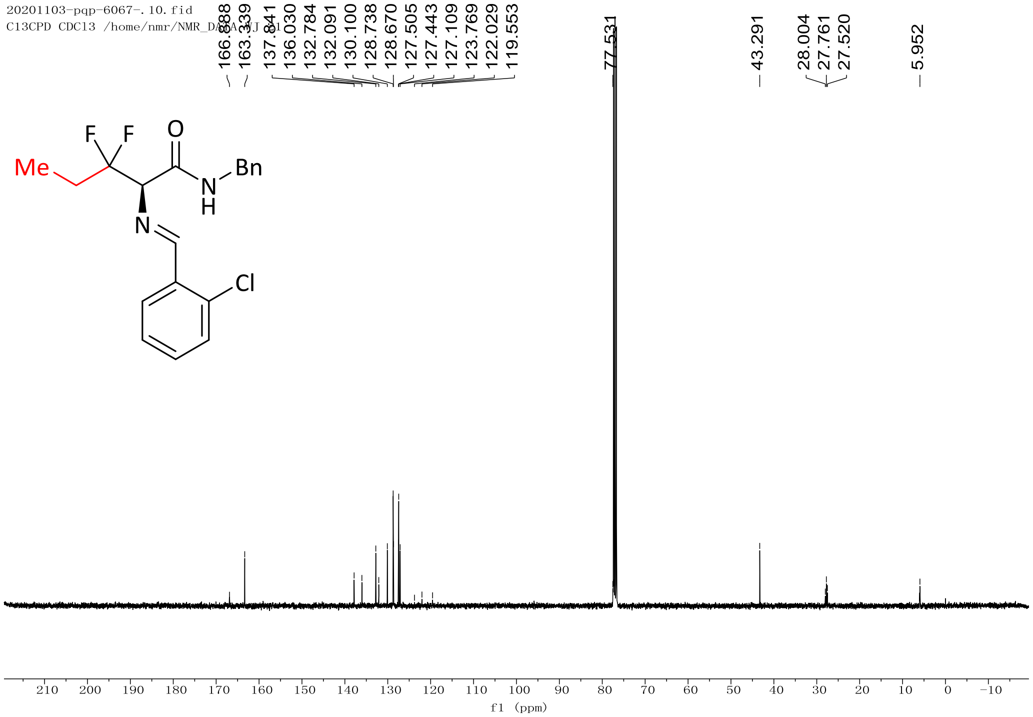

Supplementary Figure 26. ^{13}C NMR Spectra of **2h**

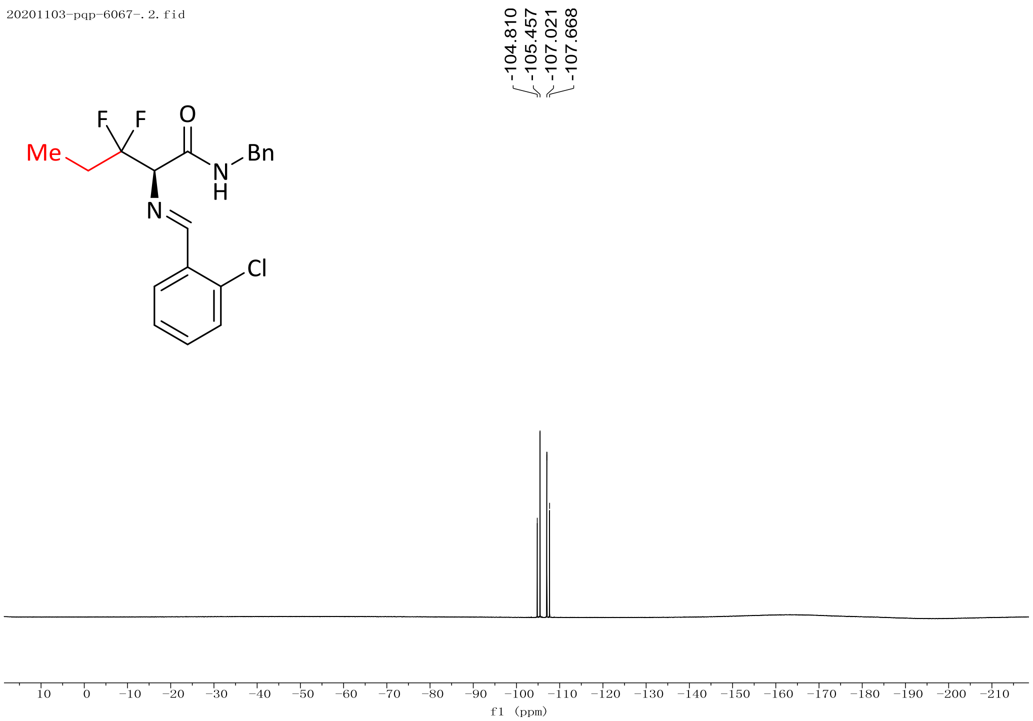

Supplementary Figure 27. ^{19}F NMR Spectra of **2h**

2i

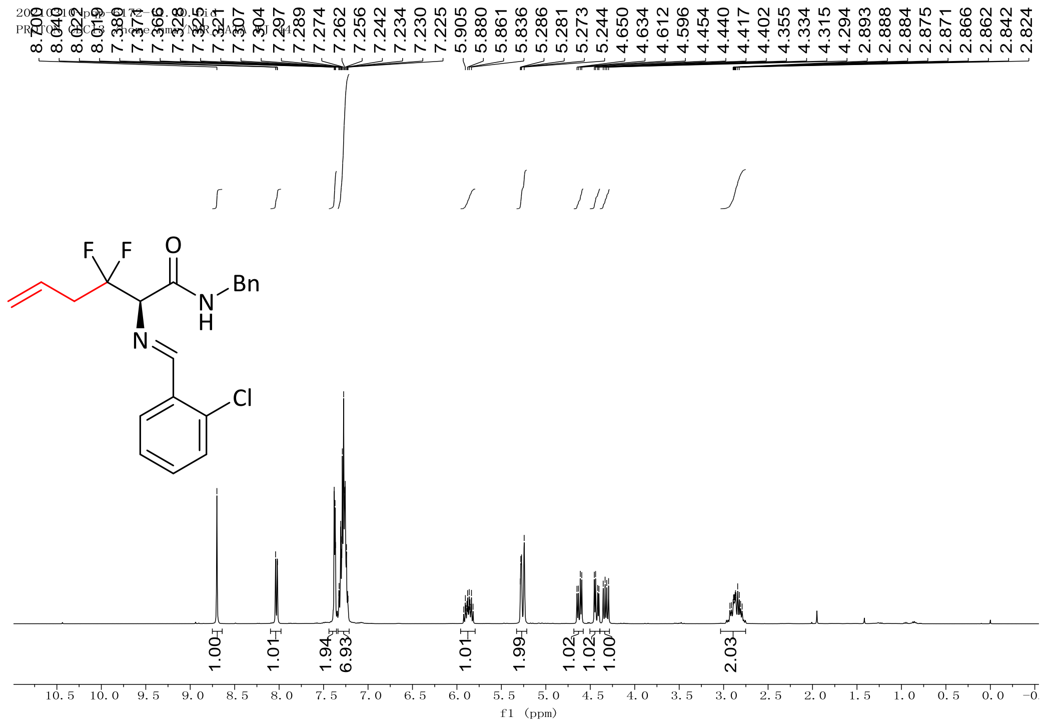

Supplementary Figure 28. ^{1}H NMR Spectra of **2i**

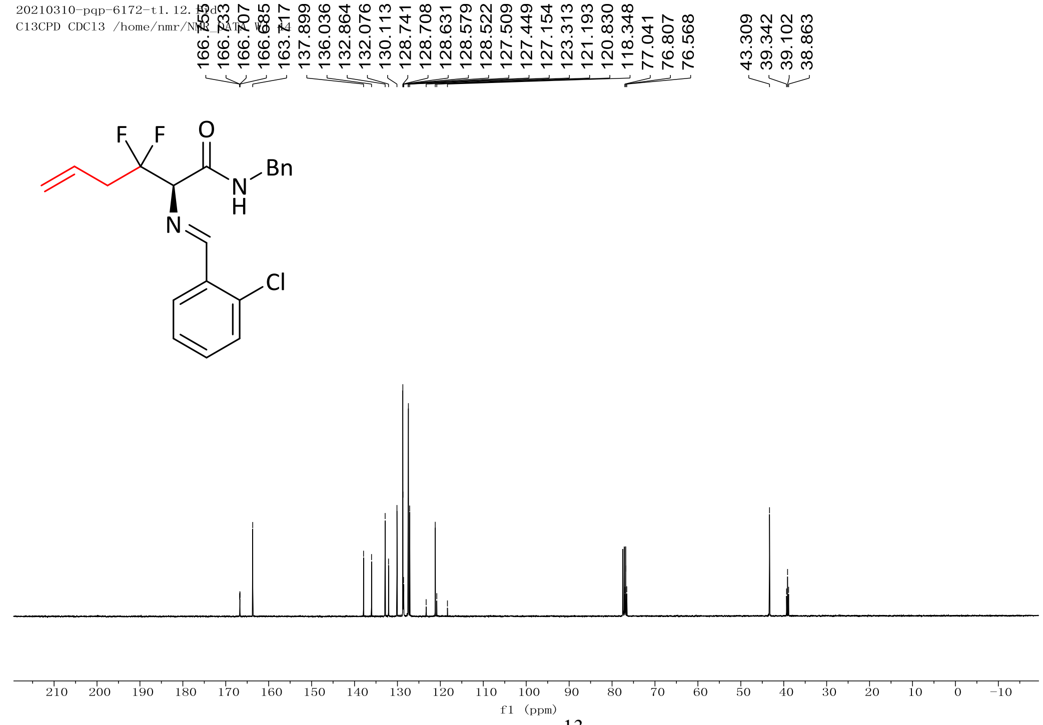

Supplementary Figure 29. ^{13}F NMR Spectra of **2i**

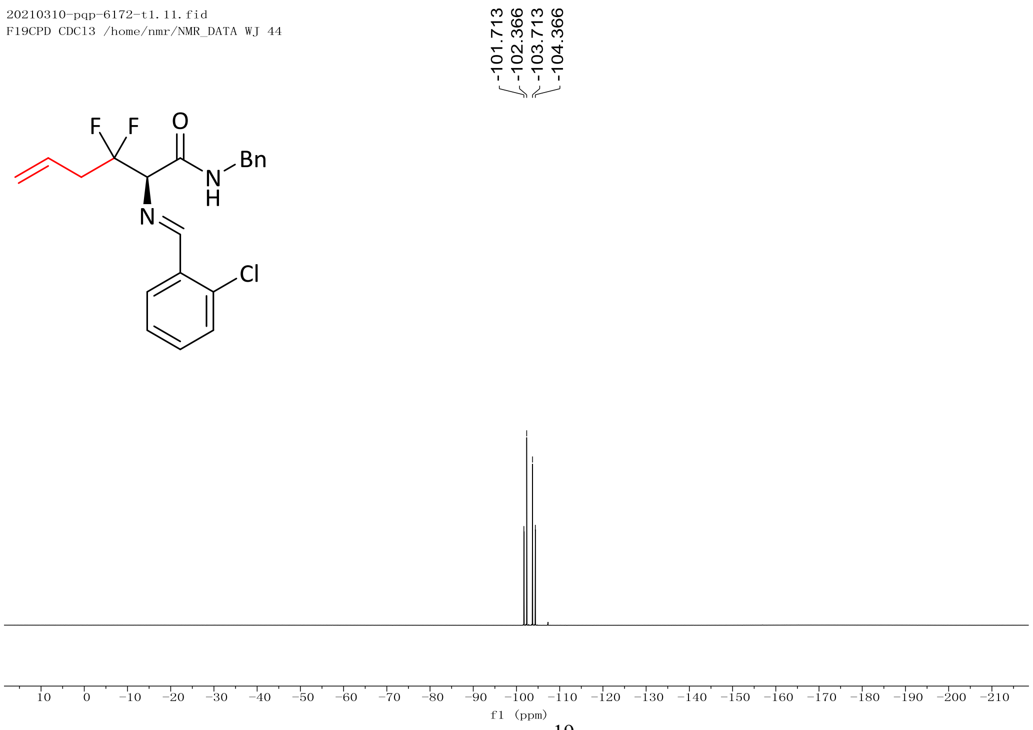

Supplementary Figure 30. ^{19}F NMR Spectra of **2i**

2j

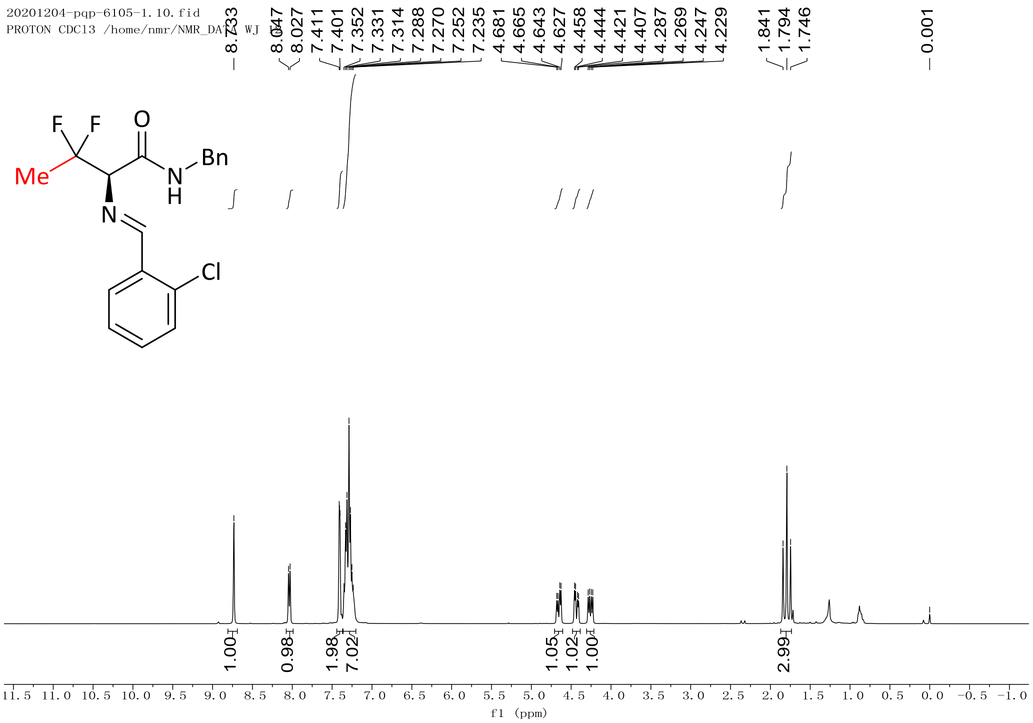

Supplementary Figure 31. ^{1}H NMR Spectra of **2j**

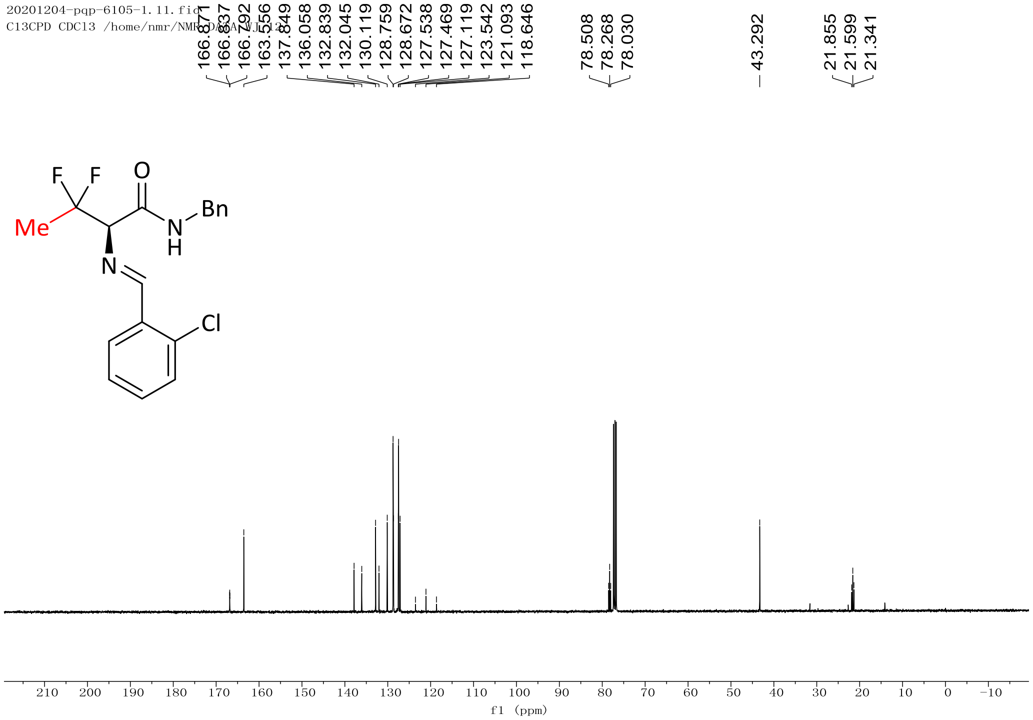

Supplementary Figure 32. ^{13}C NMR Spectra of **2j**

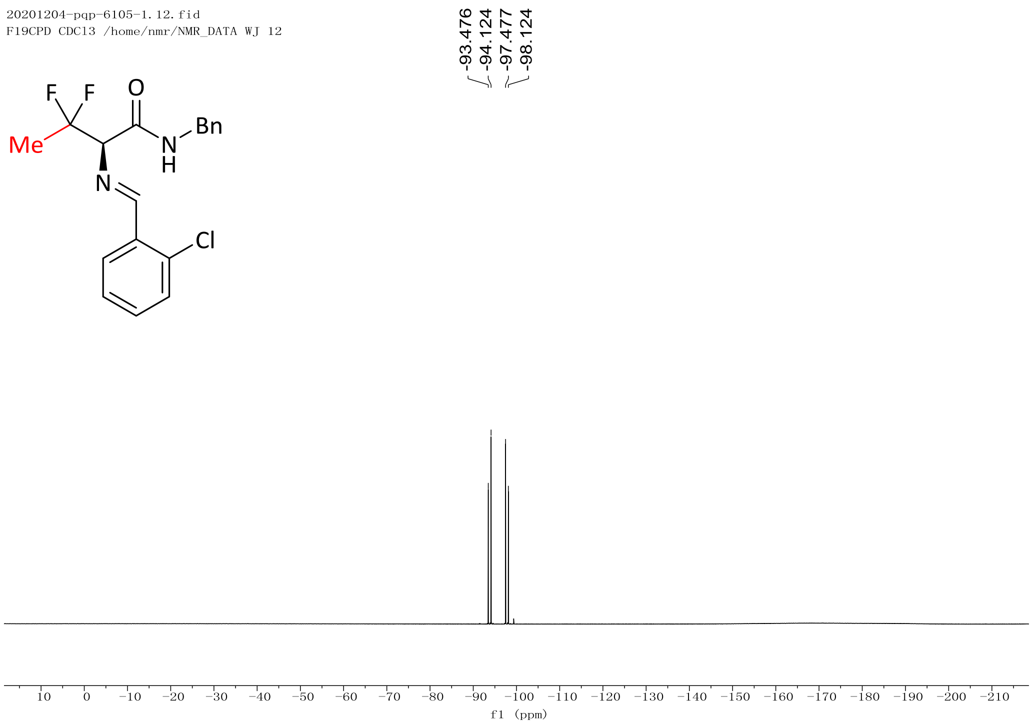

Supplementary Figure 33. ^{19}F NMR Spectra of **2j**

2k

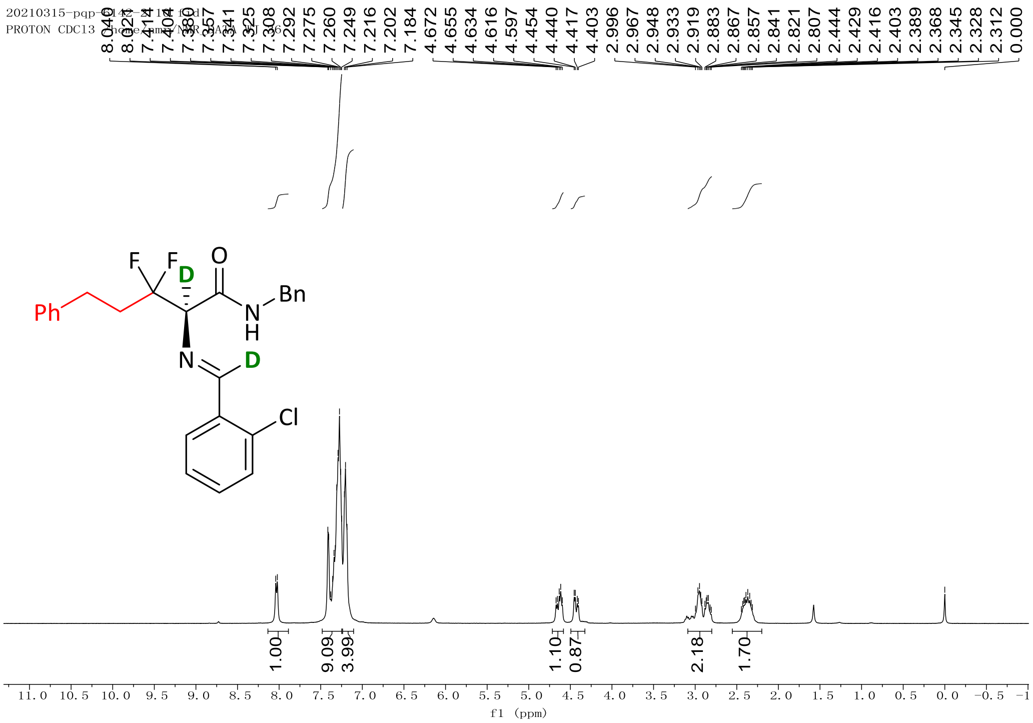

Supplementary Figure 34. ^{1}H NMR Spectra of **2k**

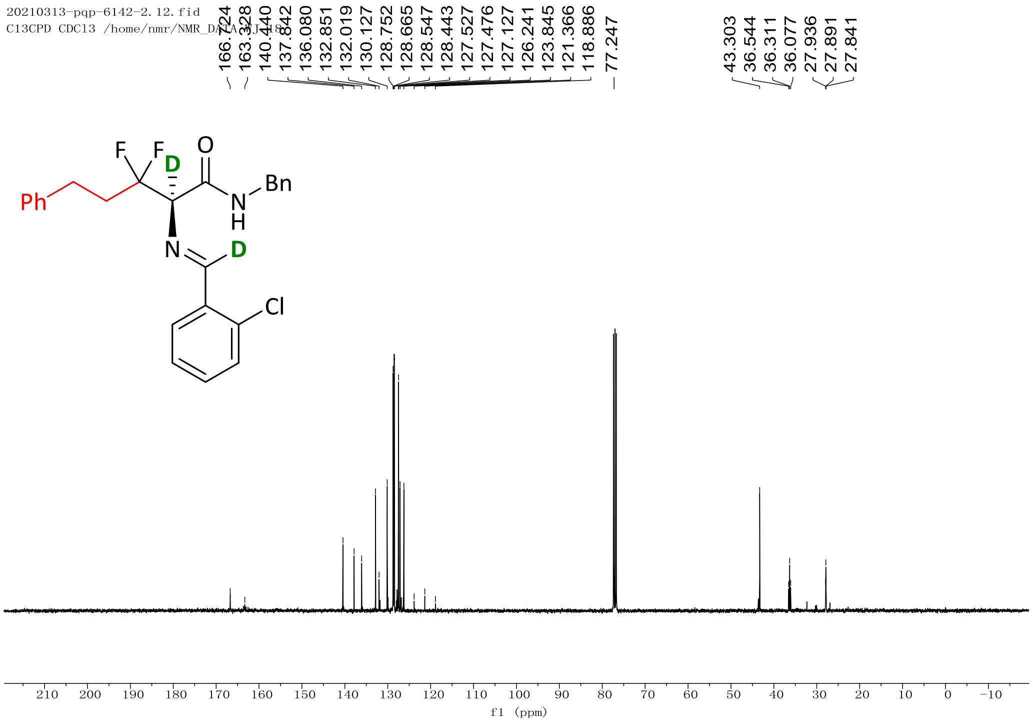

Supplementary Figure 35. ^{13}C NMR Spectra of **2k**

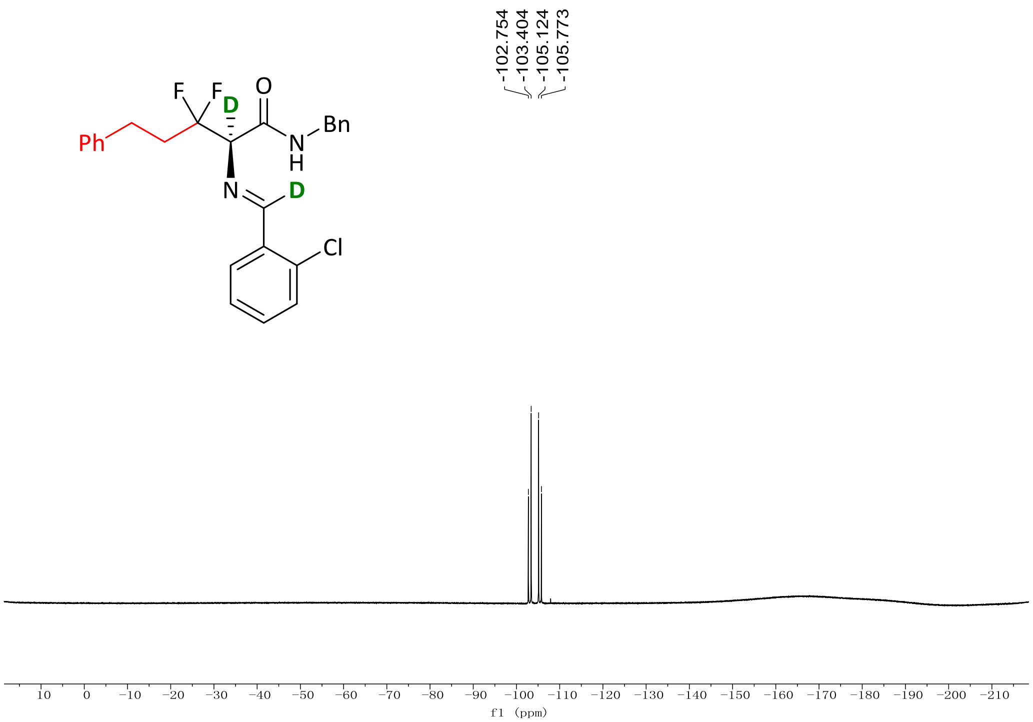

Supplementary Figure 36. ^{19}F NMR Spectra of **2k**

2l

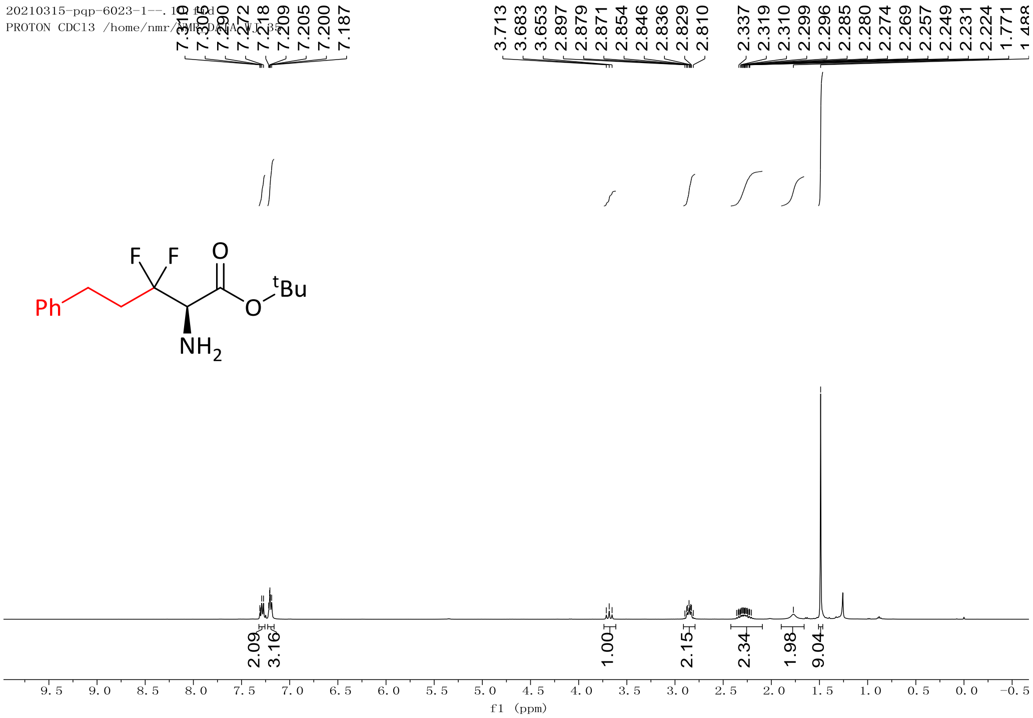

Supplementary Figure 37. ^{1}H NMR Spectra of **2l**

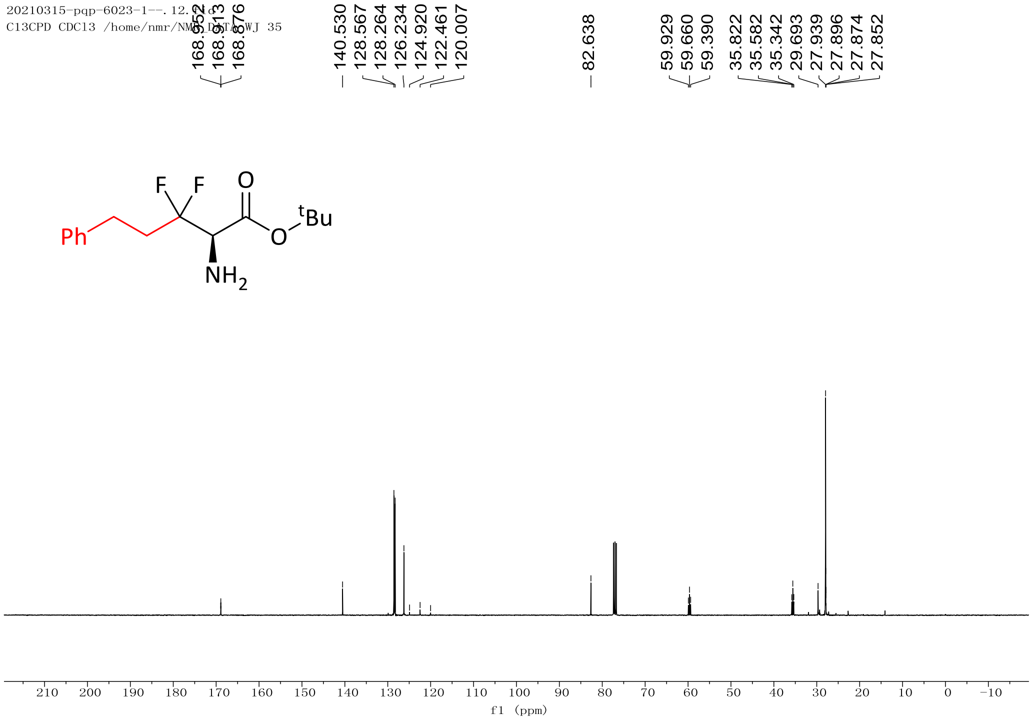

Supplementary Figure 38. ^{13}C NMR Spectra of **2l**

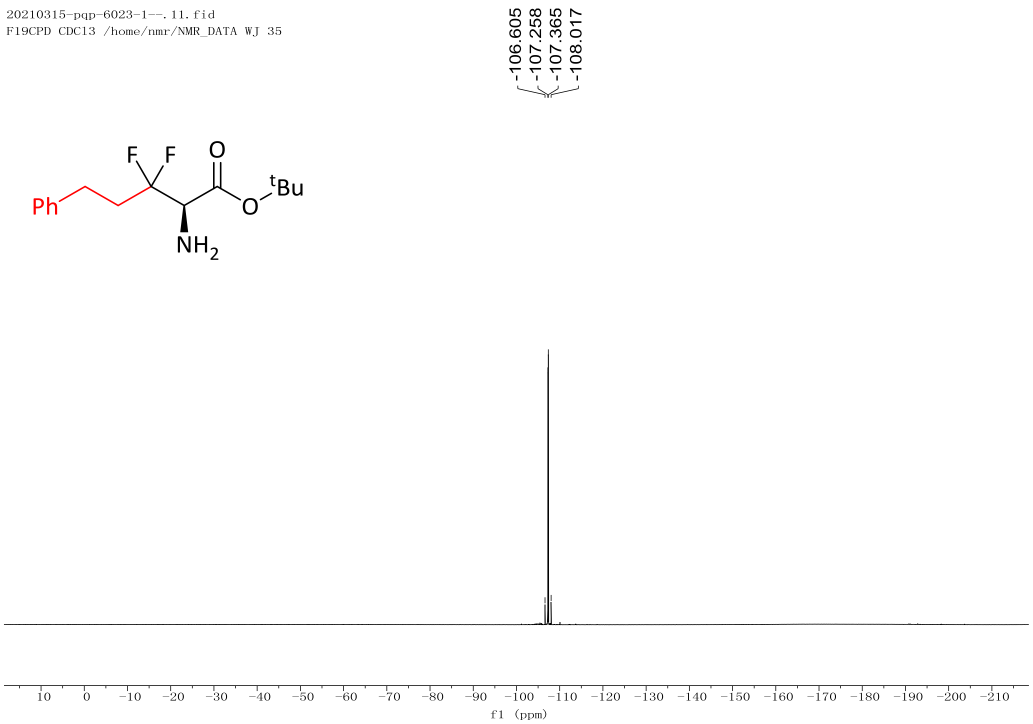

Supplementary Figure 39. ^{19}F NMR Spectra of **2l**

2m

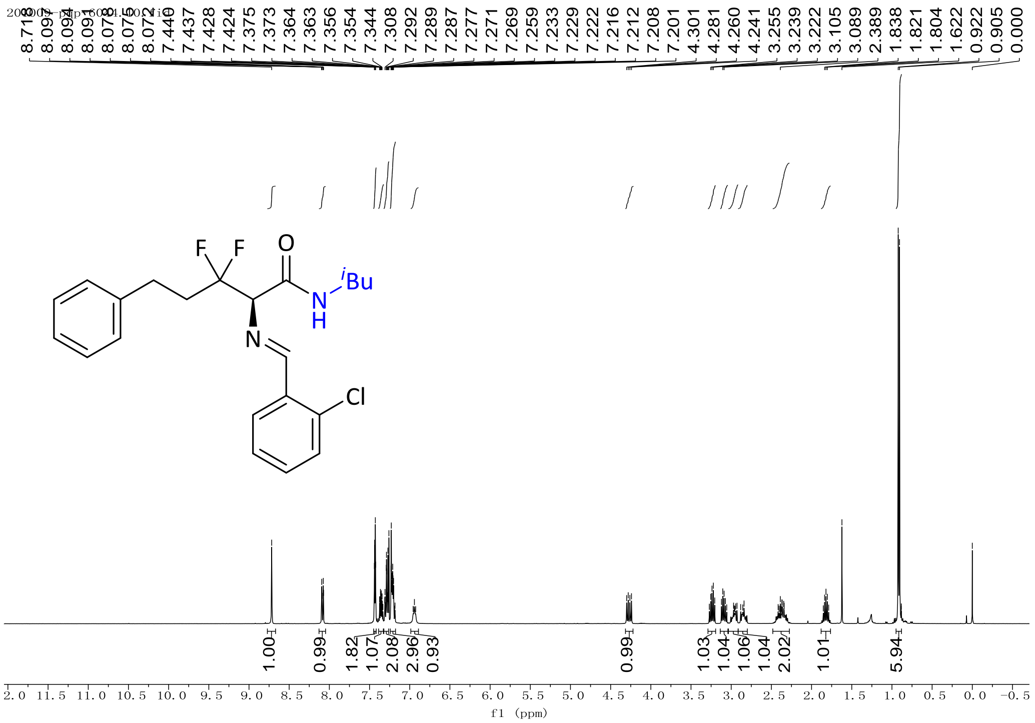

Supplementary Figure 40. ^{1}H NMR Spectra of **2m**

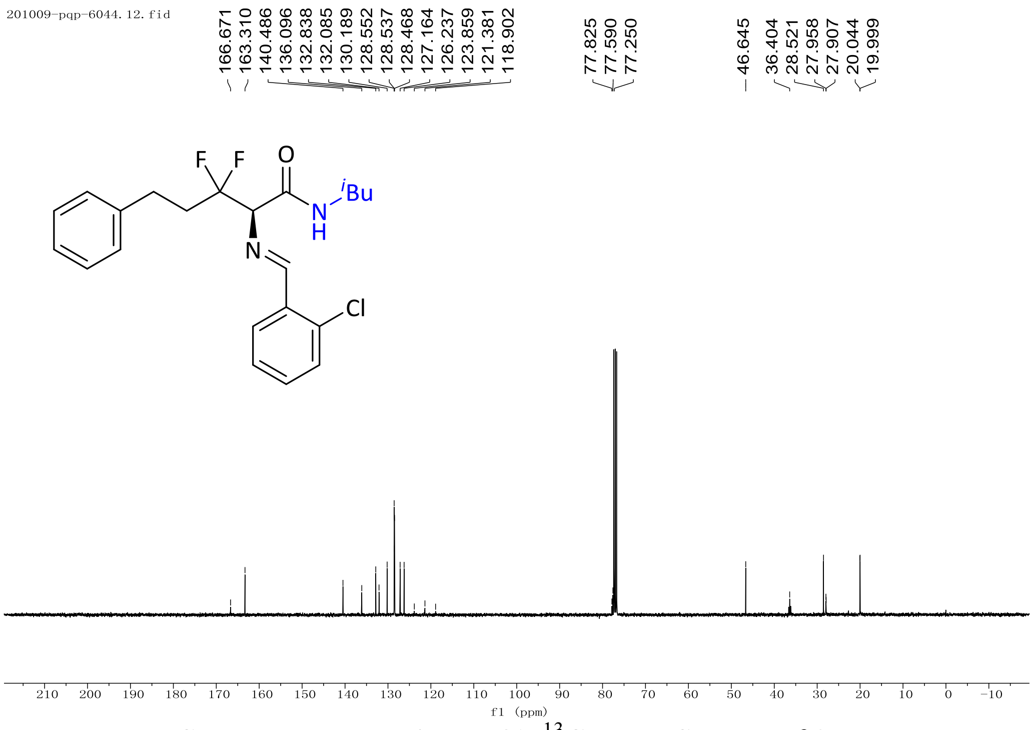

Supplementary Figure 41. ^{13}C NMR Spectra of **2m**

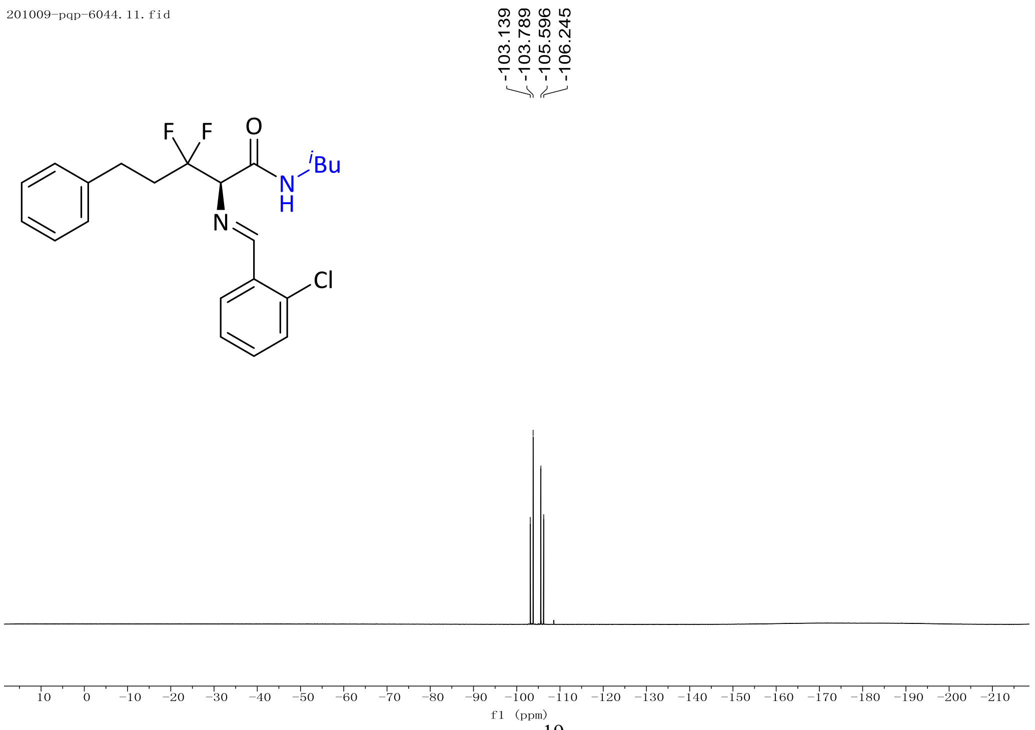

Supplementary Figure 42. ^{19}C NMR Spectra of **2m**

2n

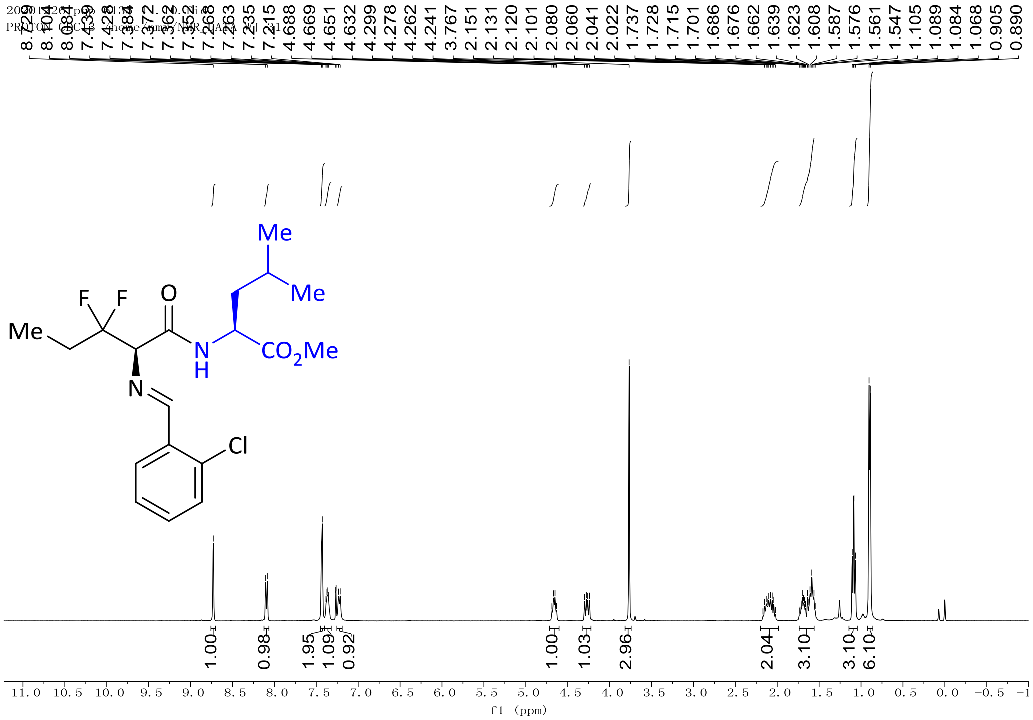

Supplementary Figure 43. ^{1}H NMR Spectra of **2n**

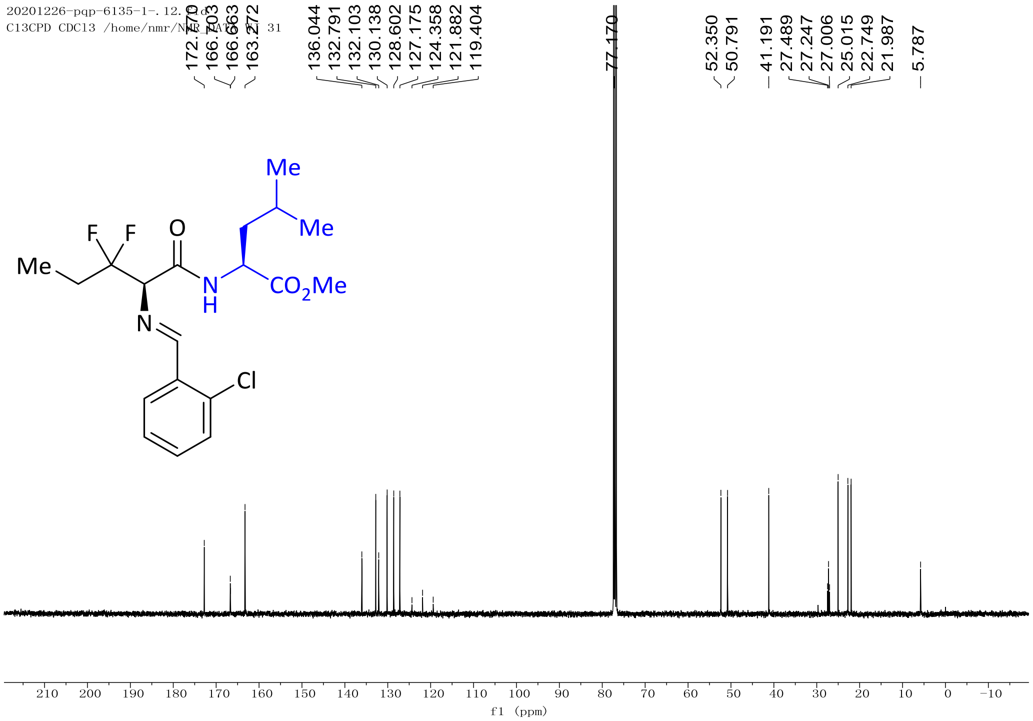

Supplementary Figure 44. ^{13}C NMR Spectra of **2n**

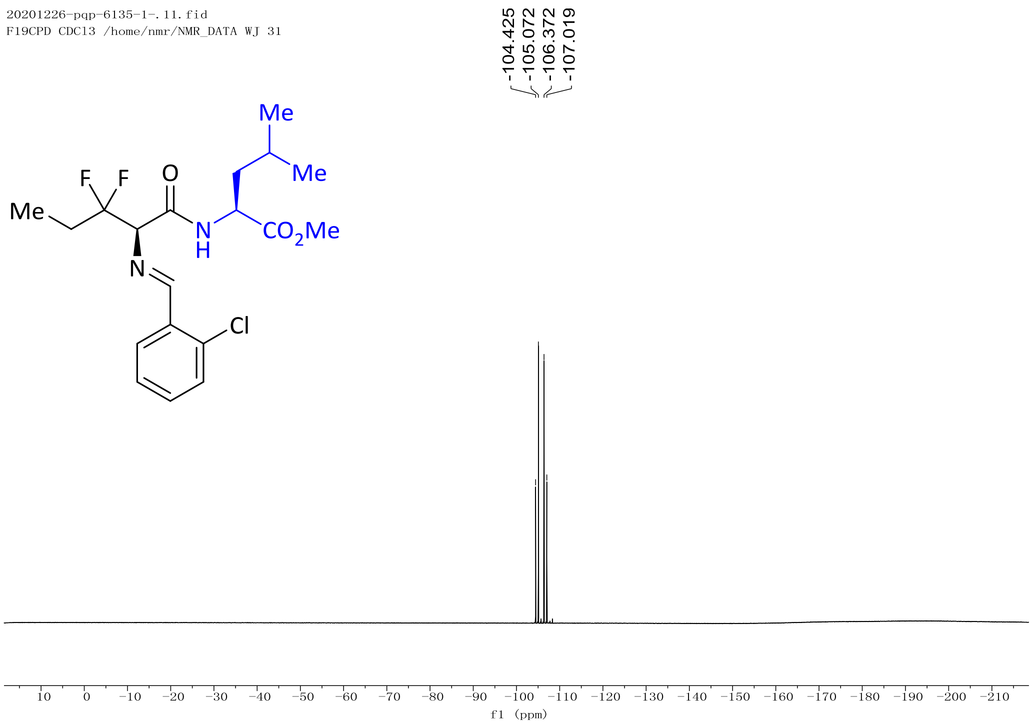

Supplementary Figure 45. ^{19}F NMR Spectra of **2n**

2o

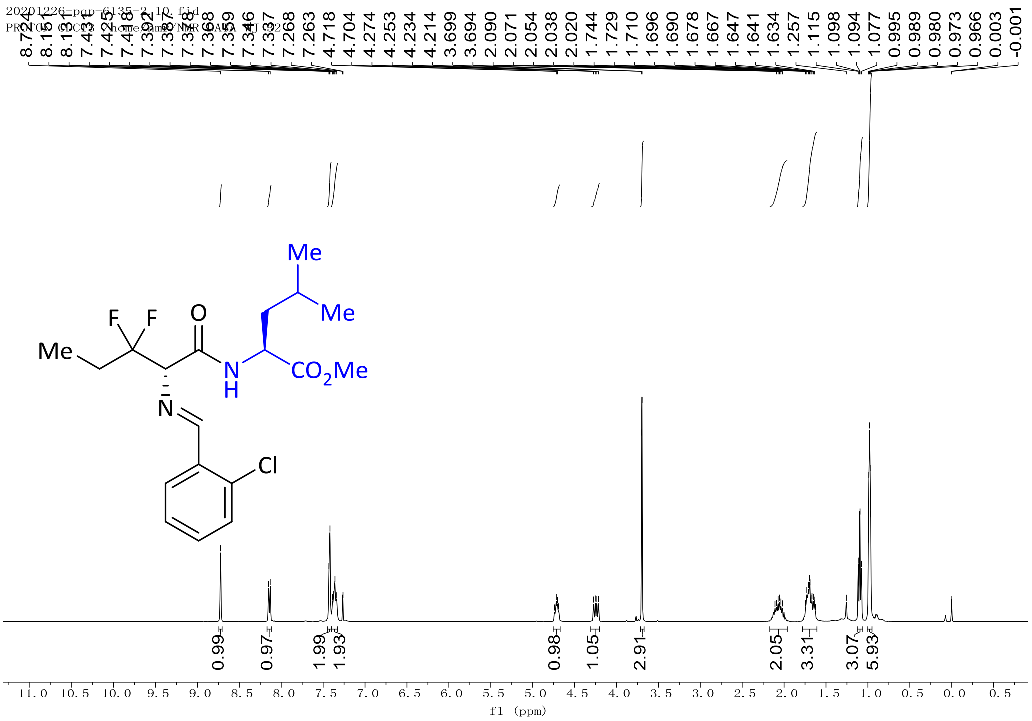

Supplementary Figure 46. ^{1}H NMR Spectra of **2o**

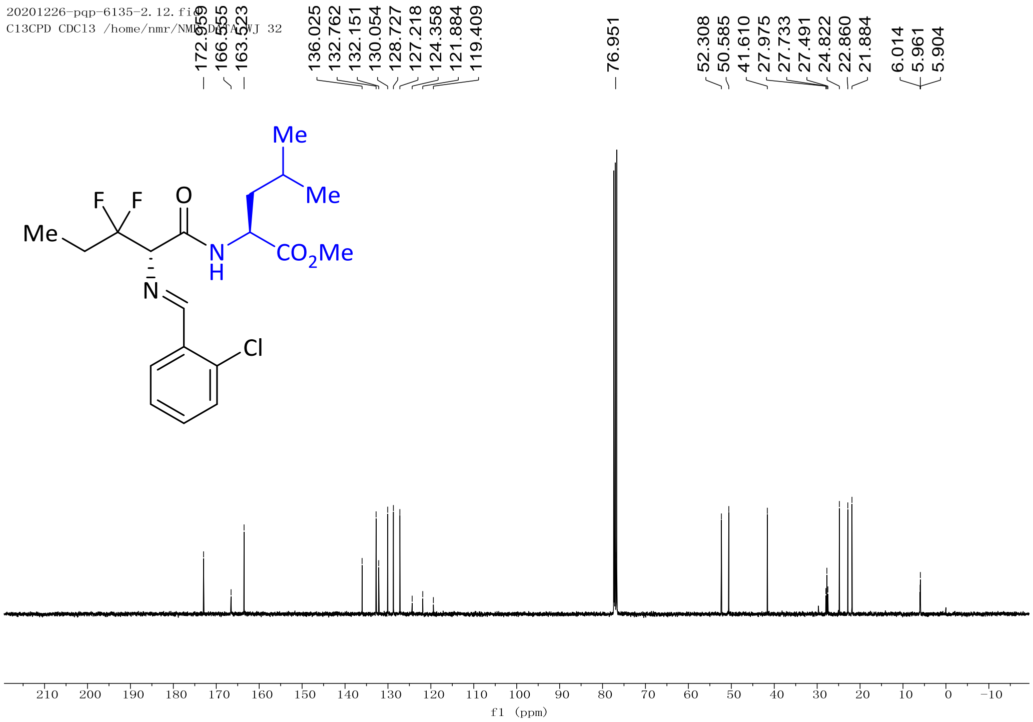

Supplementary Figure 47. ^{13}C NMR Spectra of **2o**

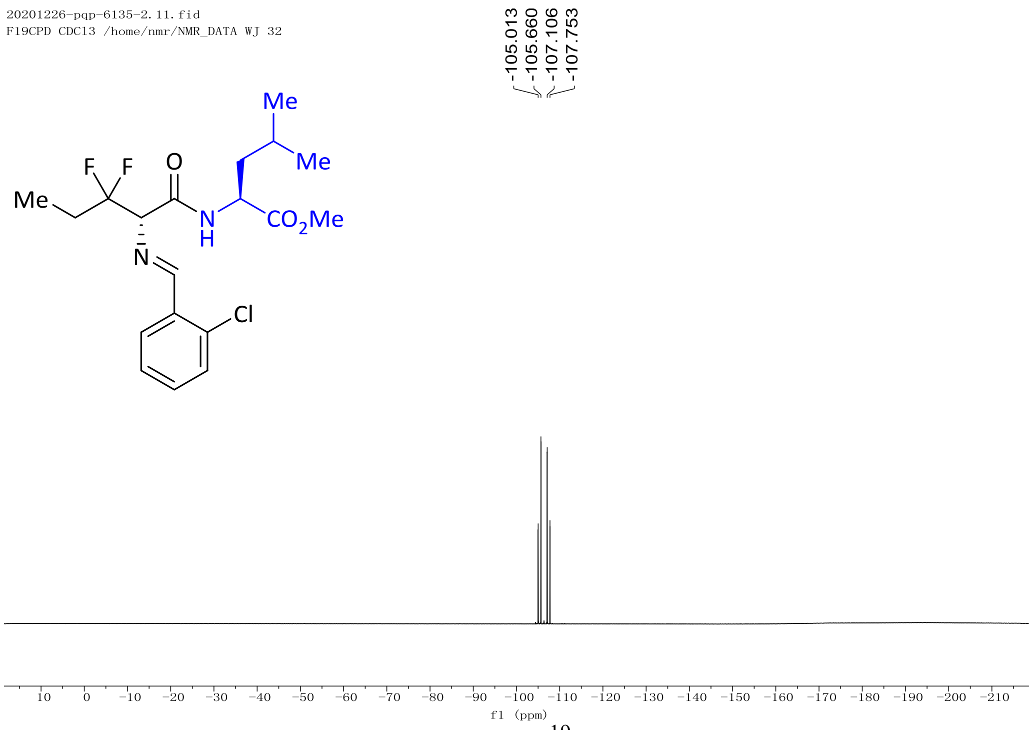

Supplementary Figure 48. ^{19}F NMR Spectra of **2o**

2p

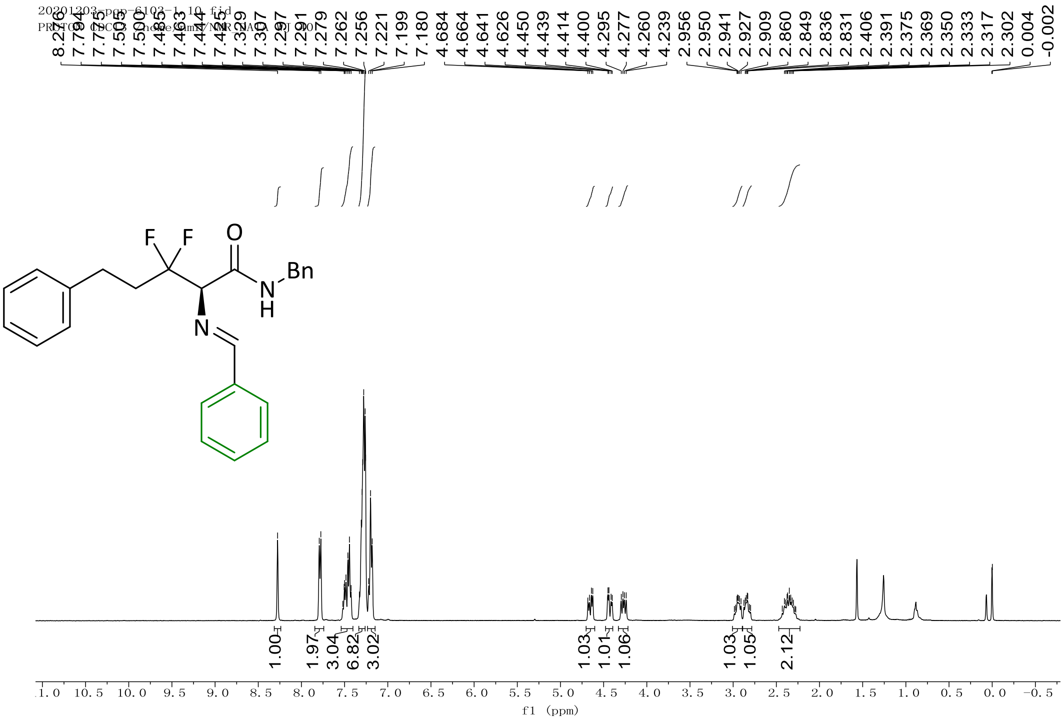

Supplementary Figure 49. ^{1}H NMR Spectra of **2p**

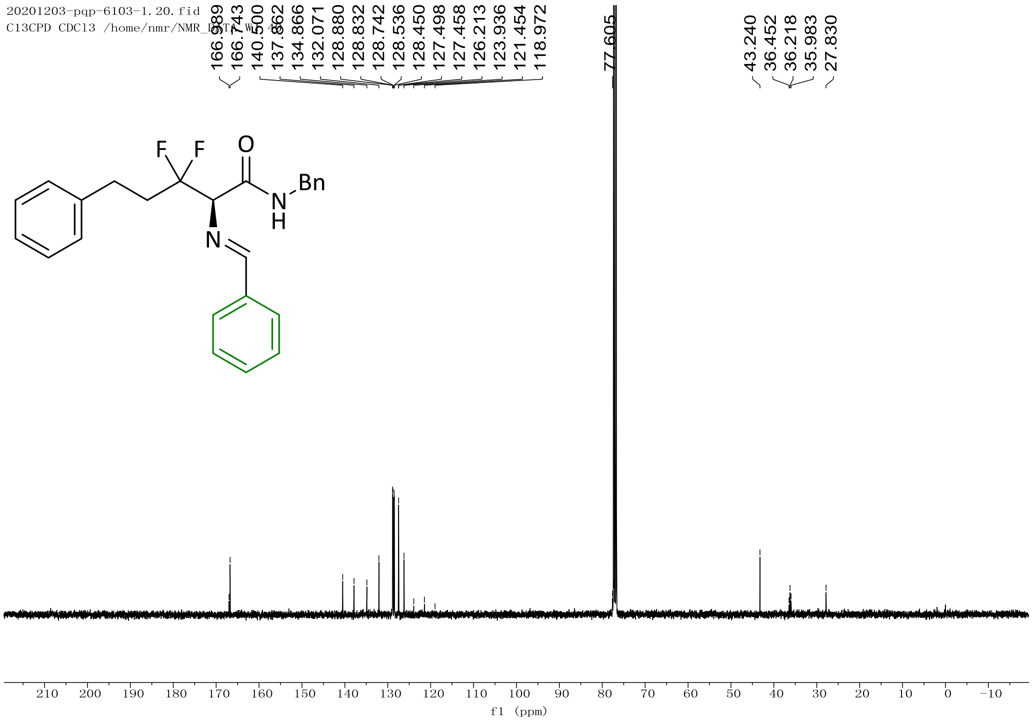

Supplementary Figure 50. ^{13}C NMR Spectra of **2p**

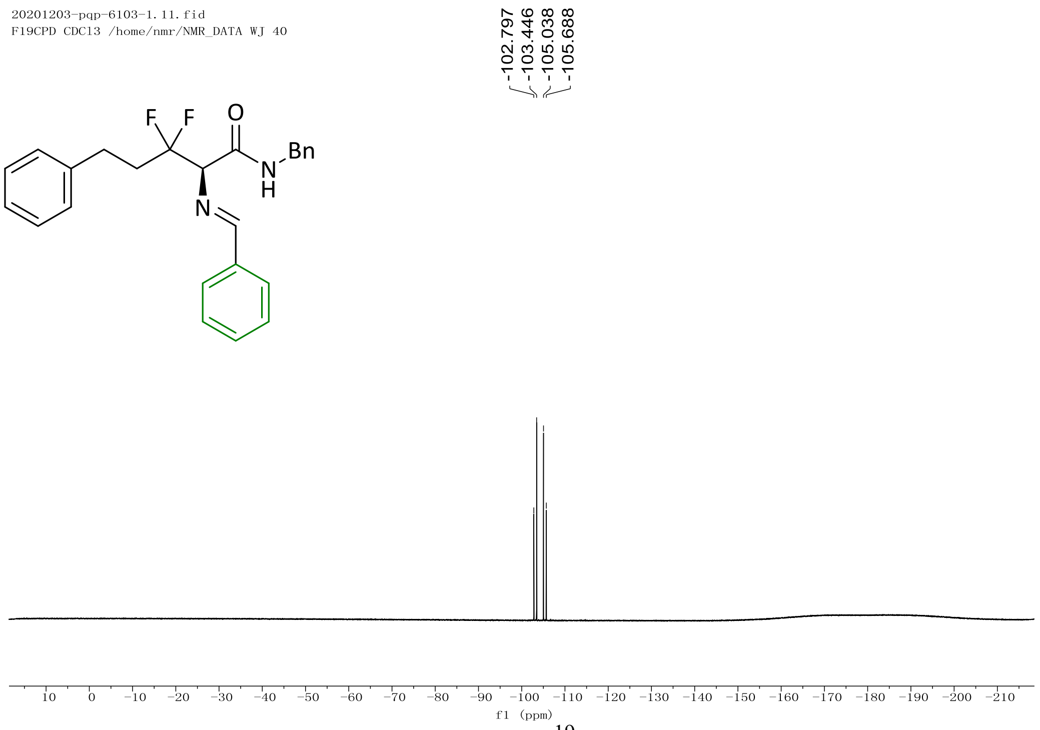

Supplementary Figure 51. ^{19}F NMR Spectra of **2p**

2q

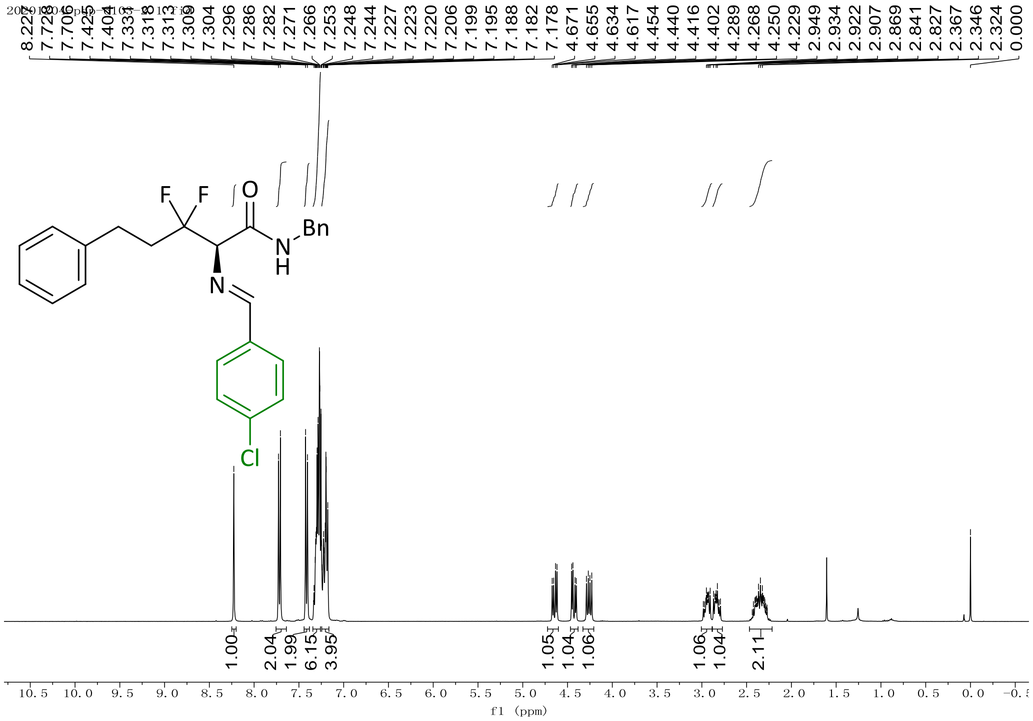

Supplementary Figure 52. ^{1}H NMR Spectra of **2q**

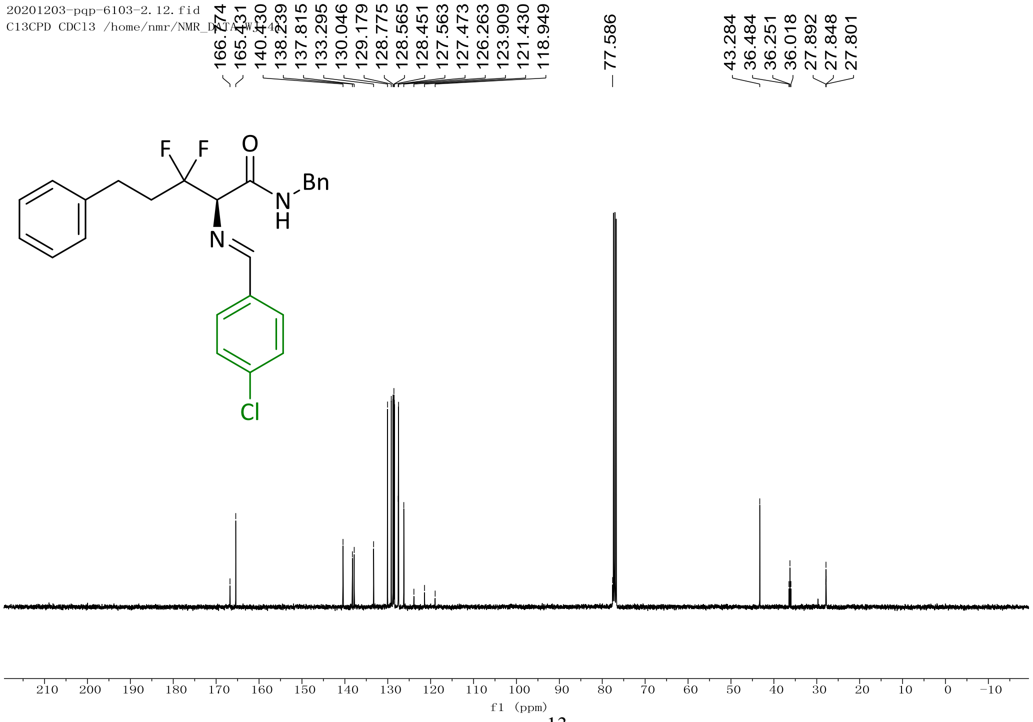

Supplementary Figure 53. ^{13}C NMR Spectra of **2q**

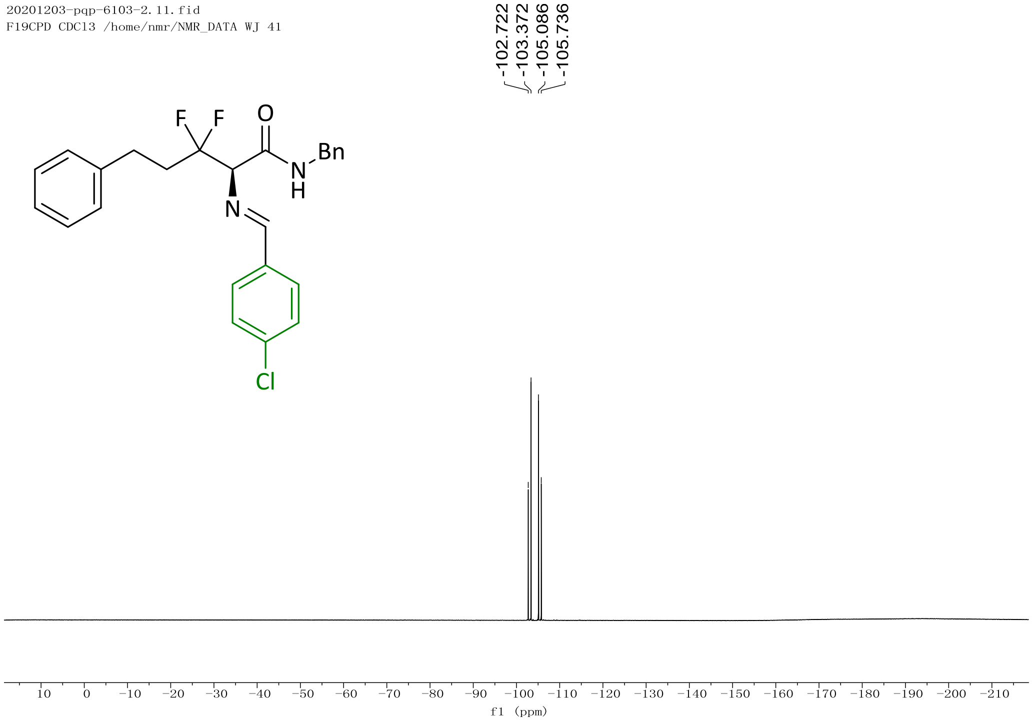

Supplementary Figure 54. ^{19}F NMR Spectra of **2q**

2r

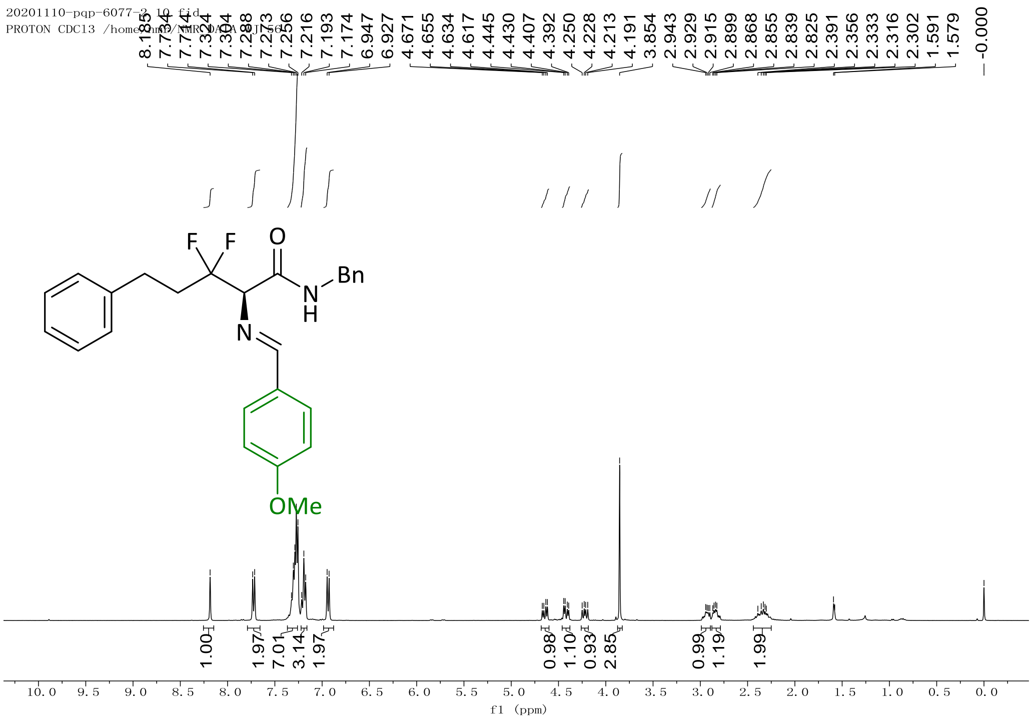

Supplementary Figure 55. ^{1}H NMR Spectra of **2r**

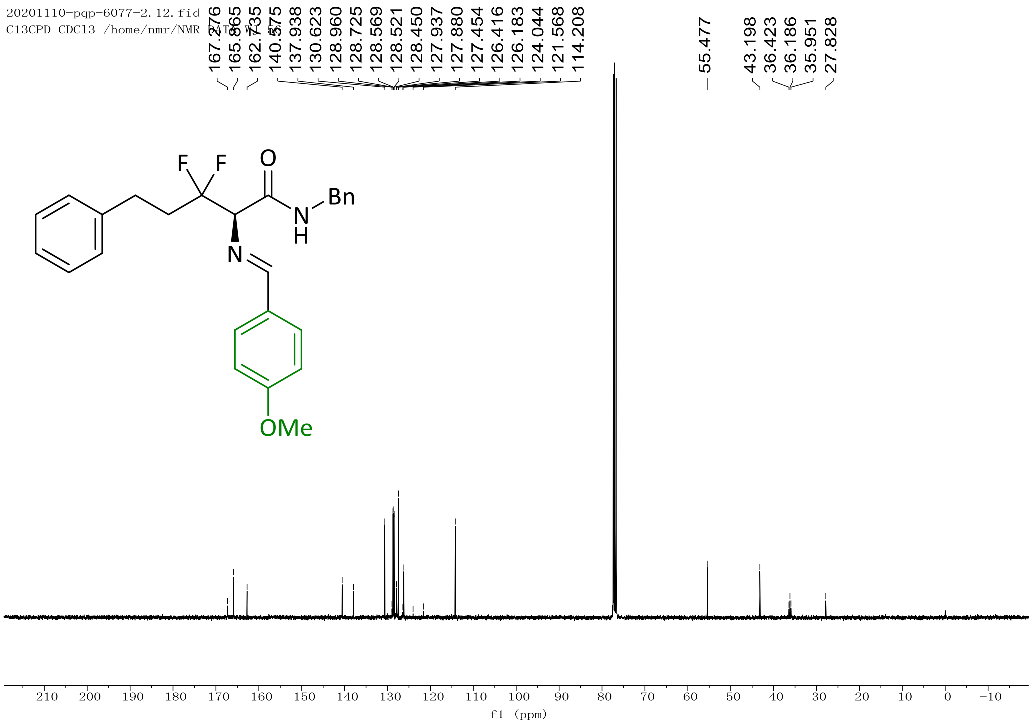

Supplementary Figure 56. ^{13}C NMR Spectra of **2r**

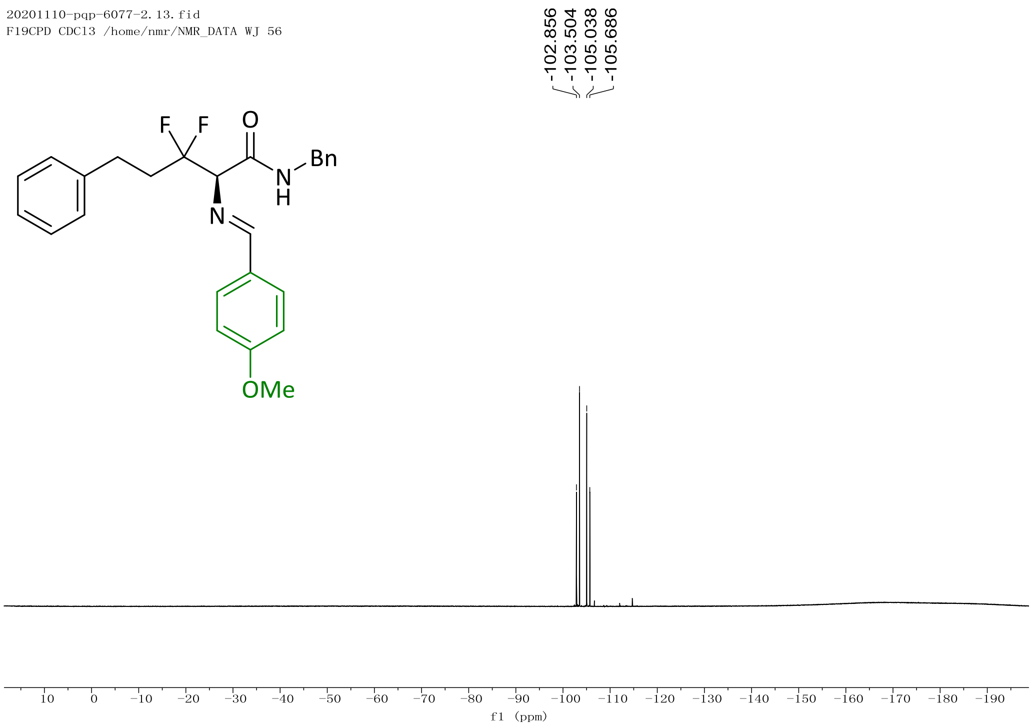

Supplementary Figure 57. ^{19}F NMR Spectra of **2r**

2s

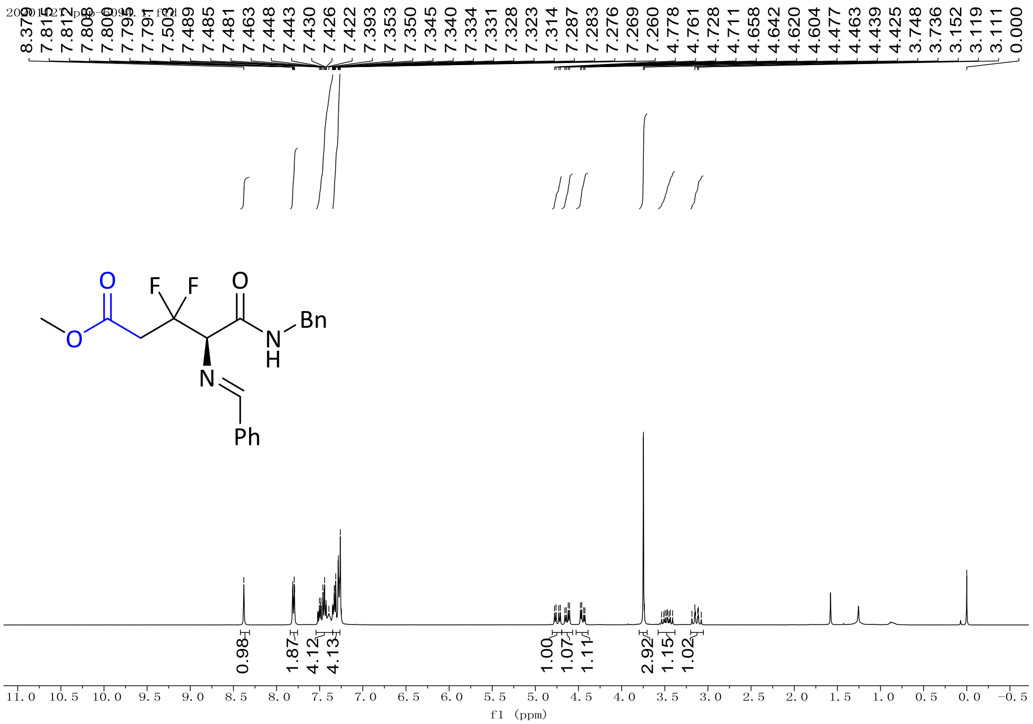

Supplementary Figure 58. ^{1}H NMR Spectra of **2s**

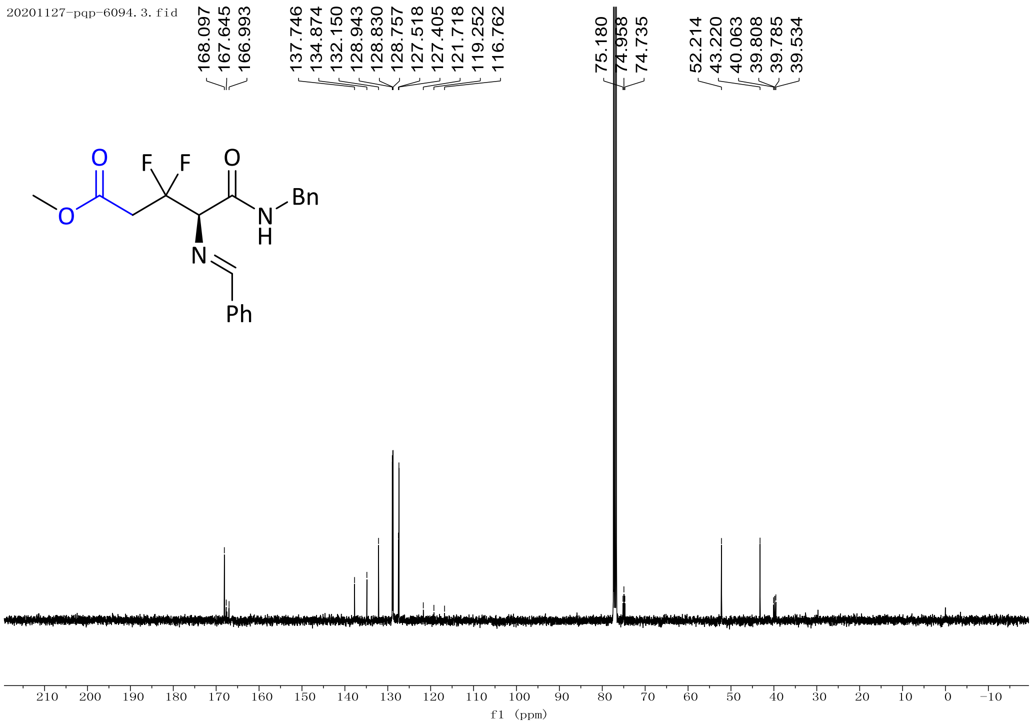

Supplementary Figure 59. ^{13}C NMR Spectra of **2s**

20201127-pqp-6094.2.fid

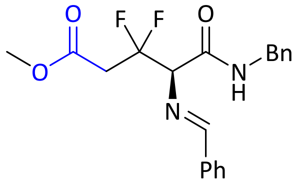

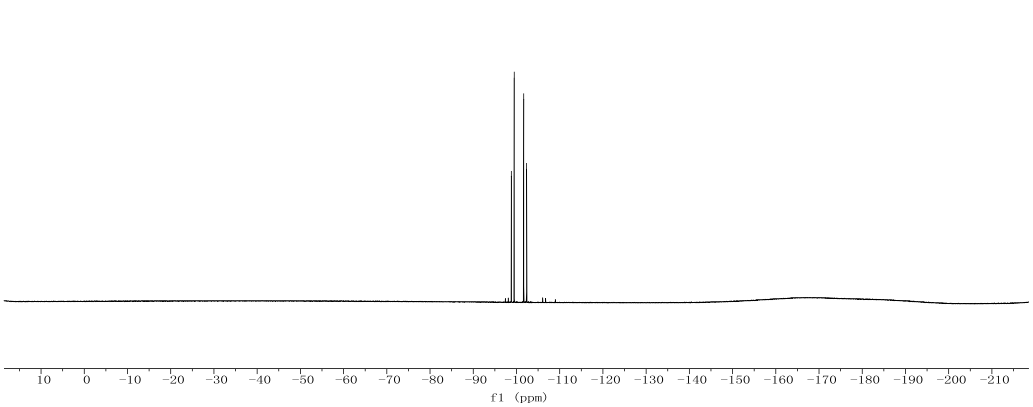

Supplementary Figure 60. ^{19}F NMR Spectra of **2s**

2t

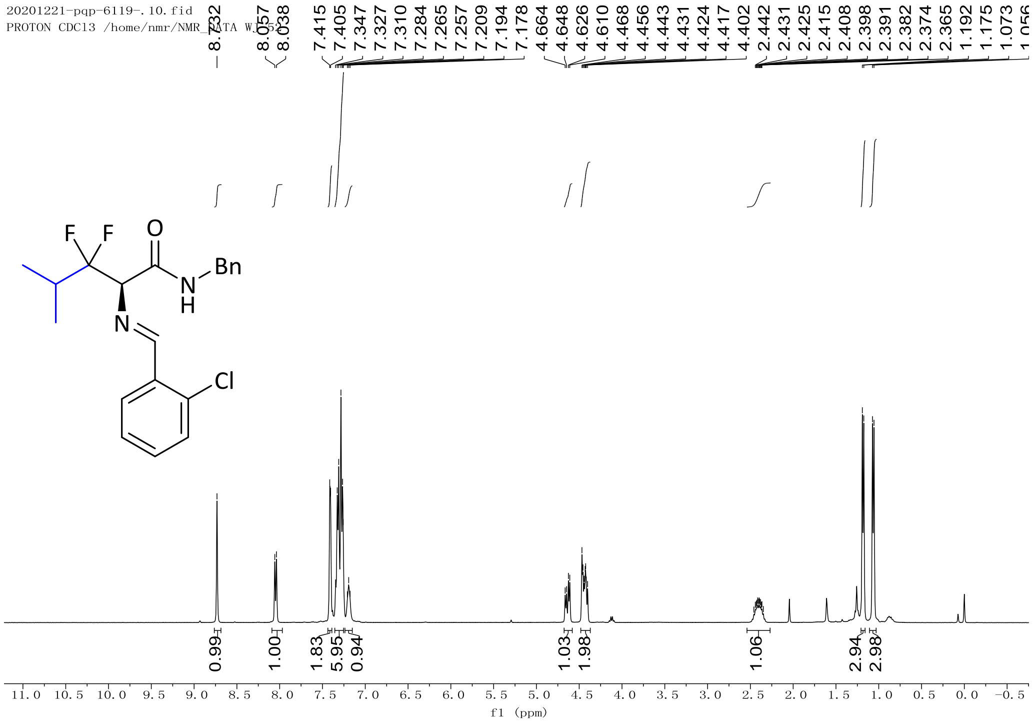

Supplementary Figure 61. ^{1}H NMR Spectra of **2t**

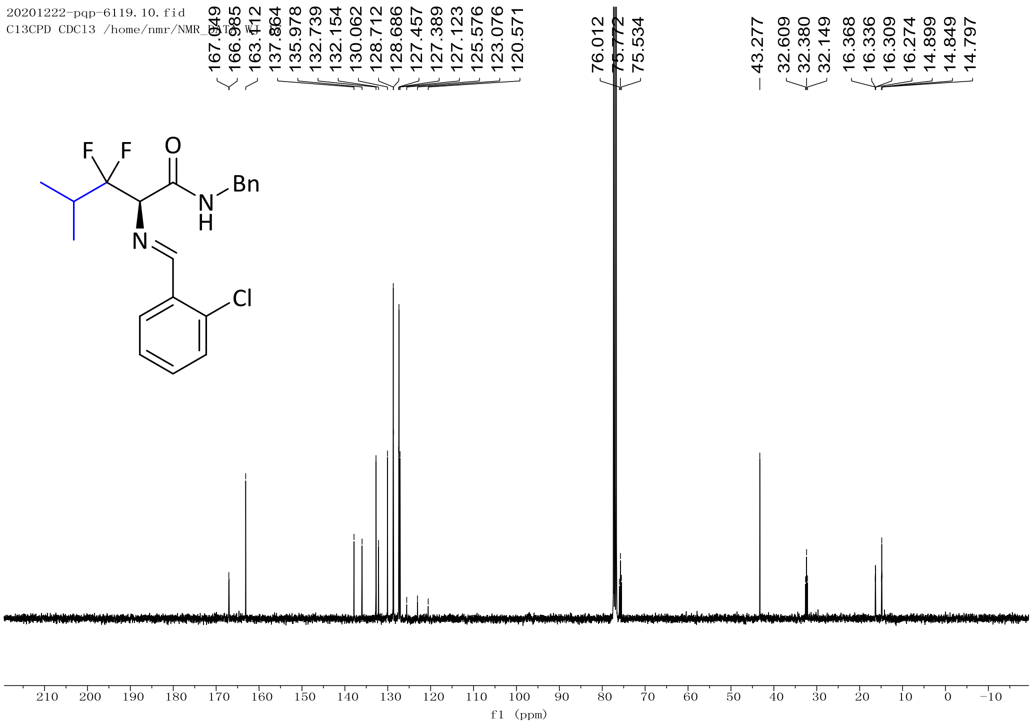

Supplementary Figure 62. ^{13}C NMR Spectra of **2t**

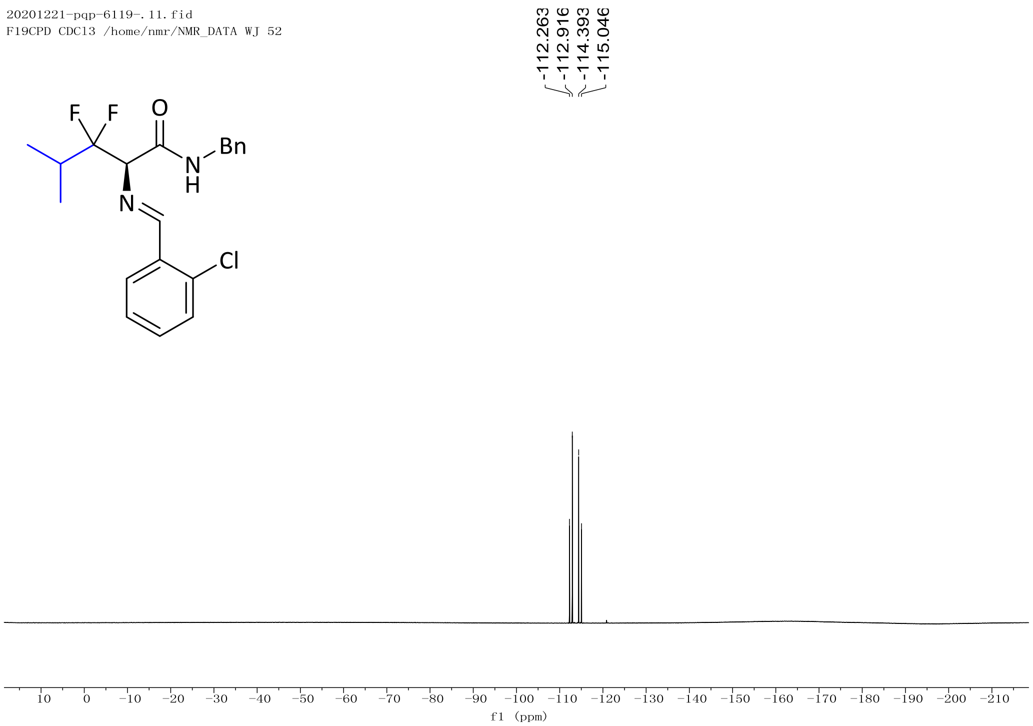

Supplementary Figure 63. ^{19}F NMR Spectra of **2t**

2u

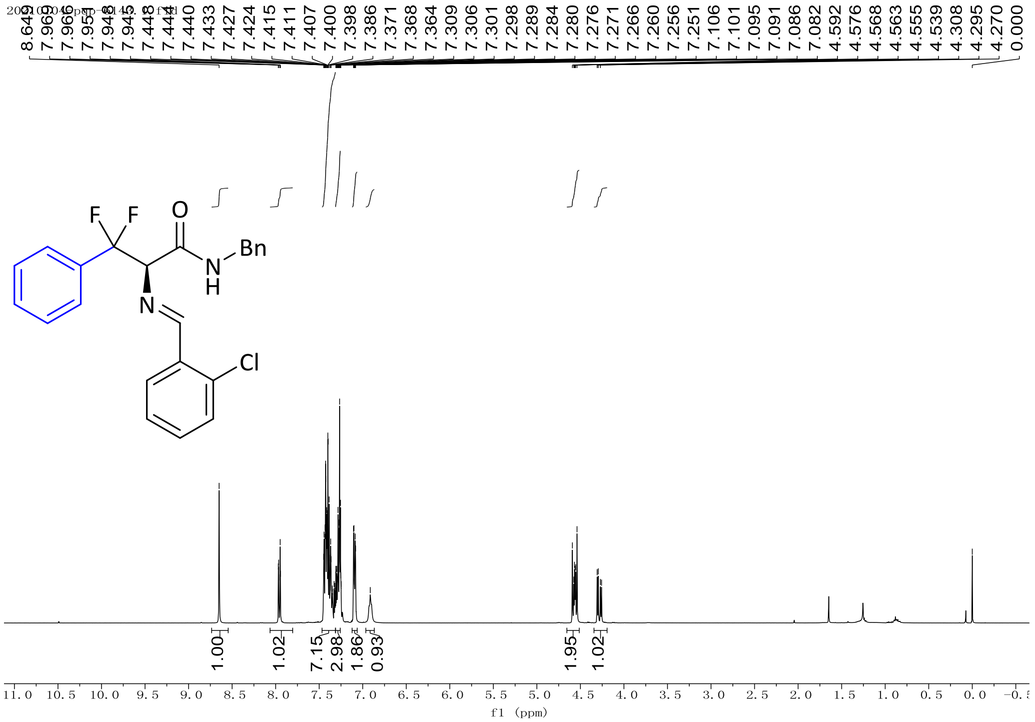

Supplementary Figure 64. ^{1}H NMR Spectra of **2u**

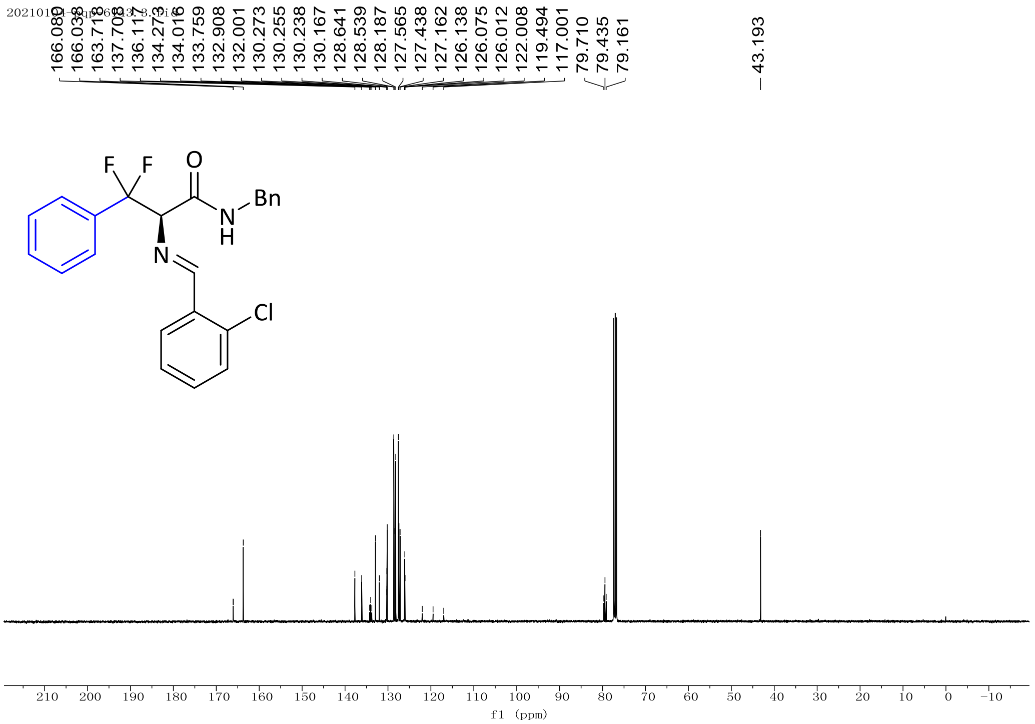

Supplementary Figure 65. ^{13}C NMR Spectra of **2u**

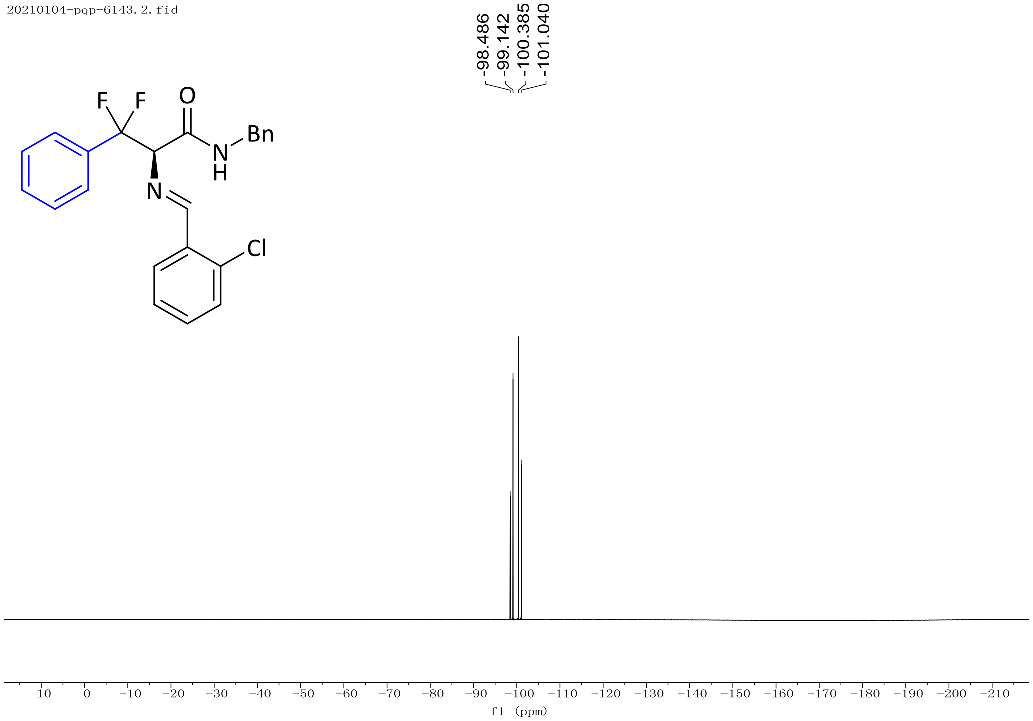

Supplementary Figure 66. ^{19}F NMR Spectra of **2u**

4a

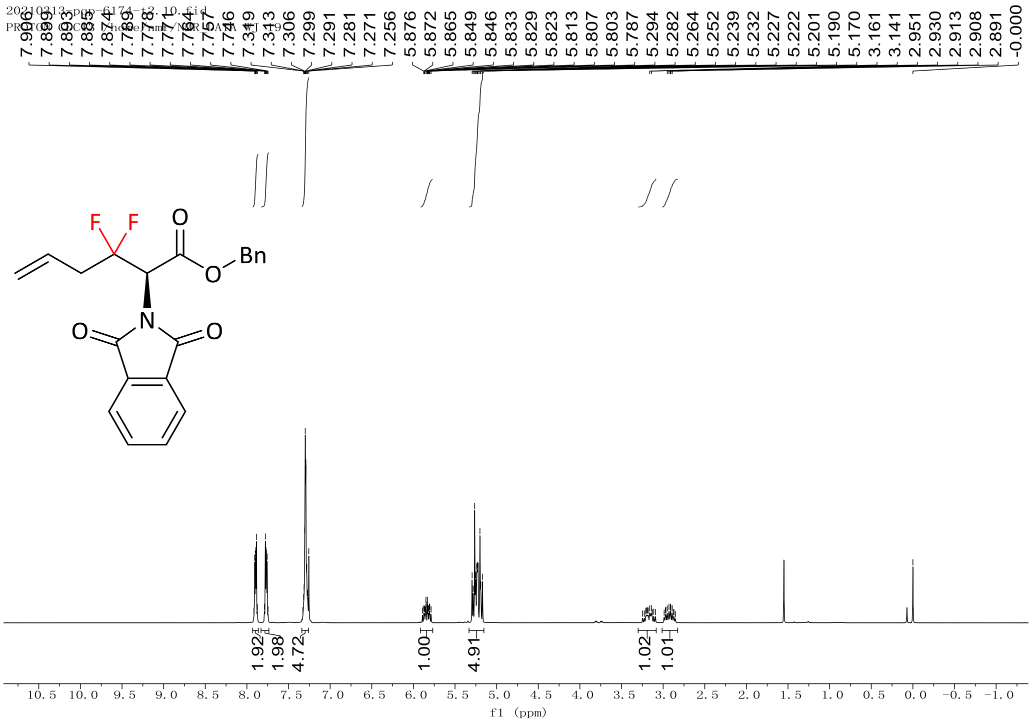

Supplementary Figure 67. ^{1}H NMR Spectra of **4a**

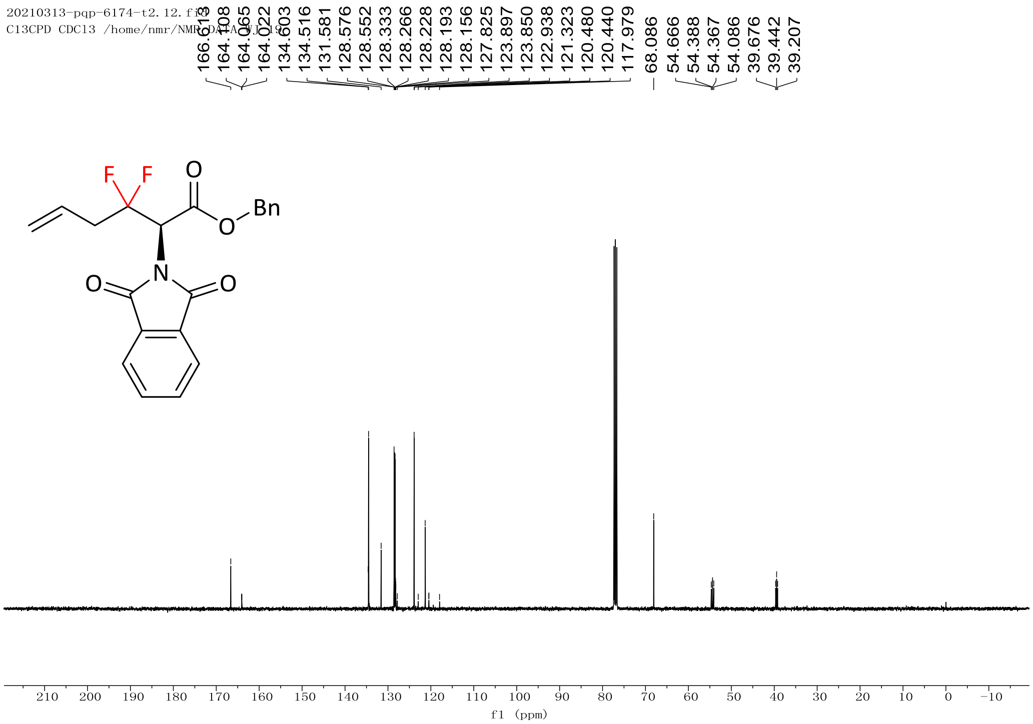

Supplementary Figure 68. ^{13}C NMR Spectra of **4a**

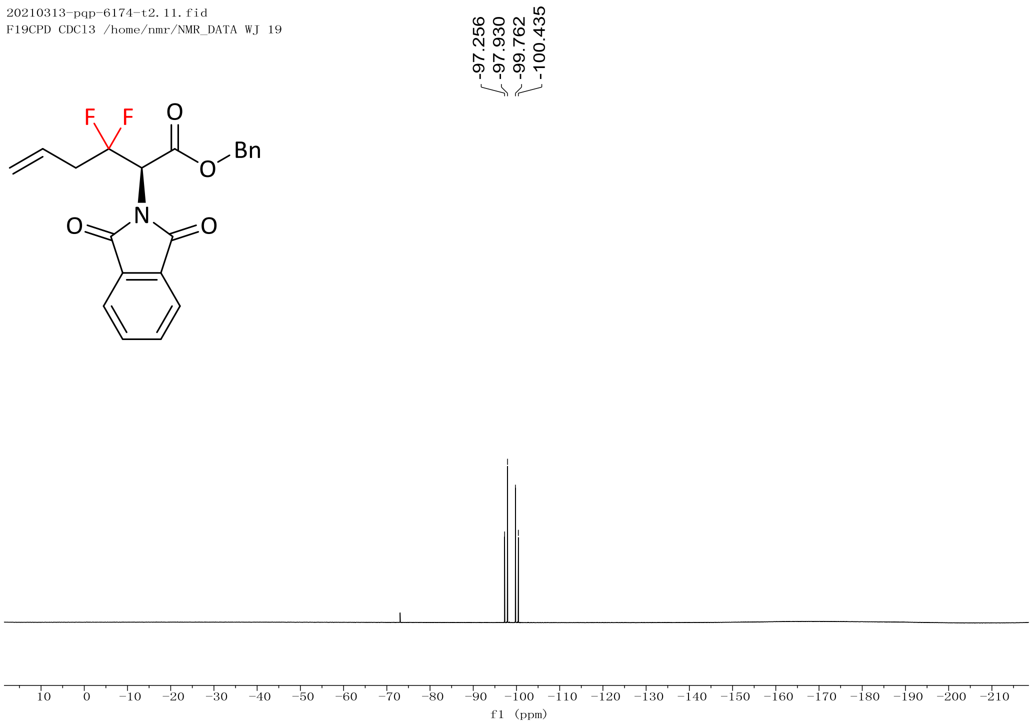

Supplementary Figure 69. ^{19}F NMR Spectra of **4a**

4b

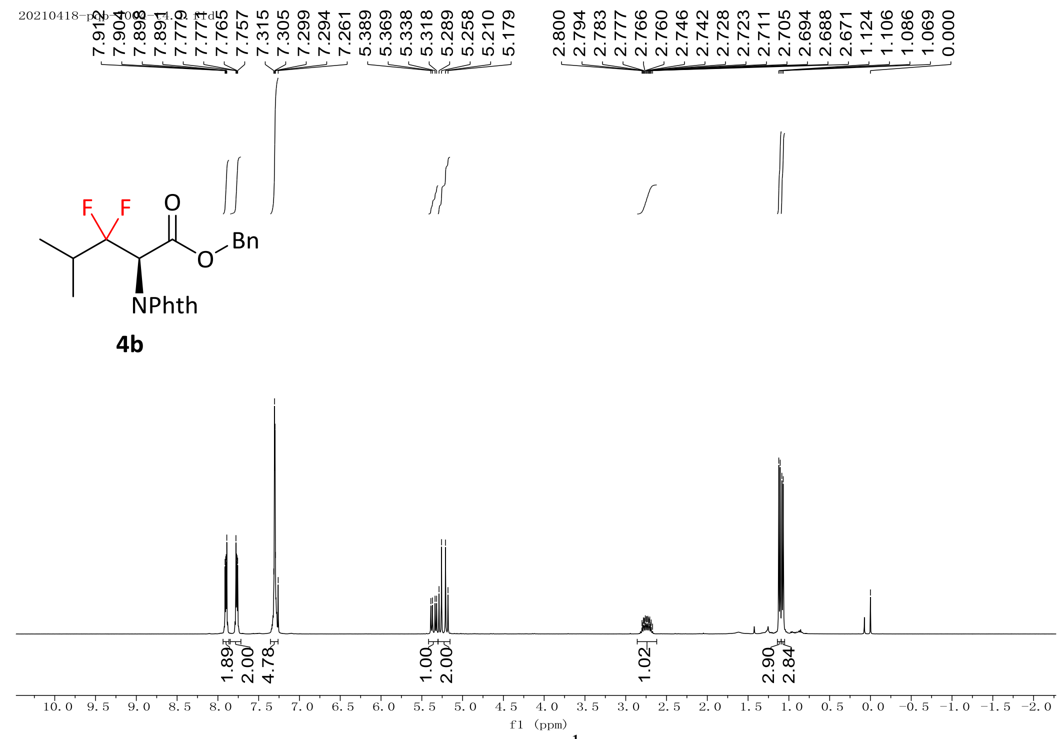

Supplementary Figure 70. ^{1}H NMR Spectra of **4b**

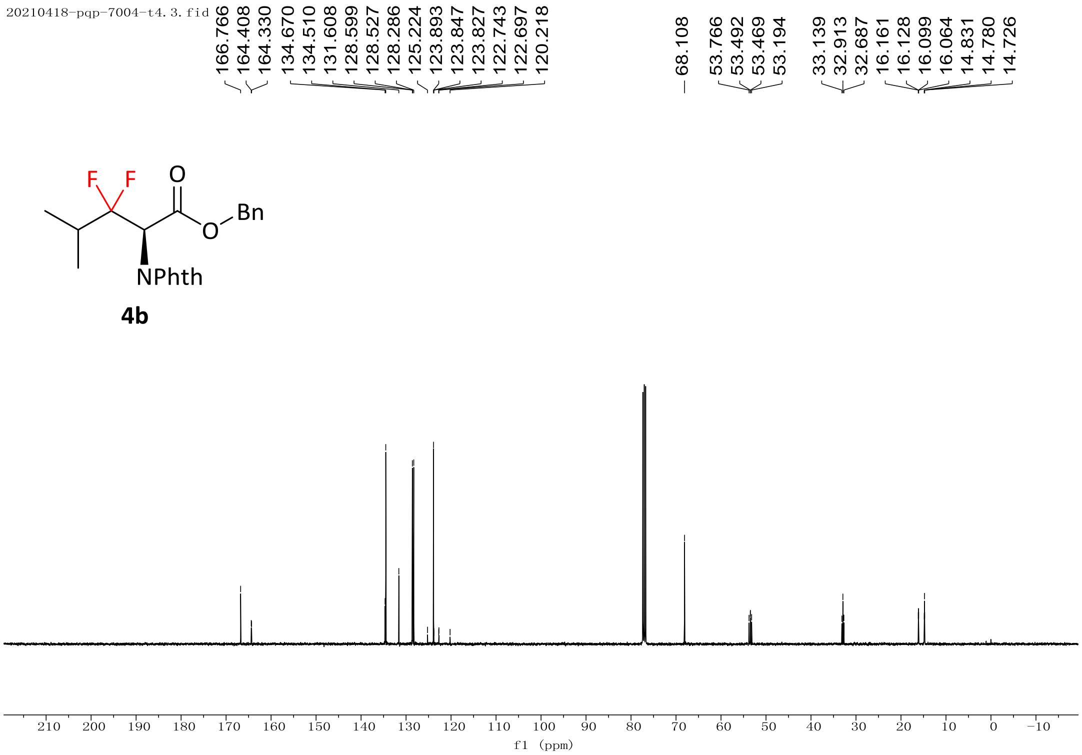

Supplementary Figure 71. ^{13}C NMR Spectra of **4b**

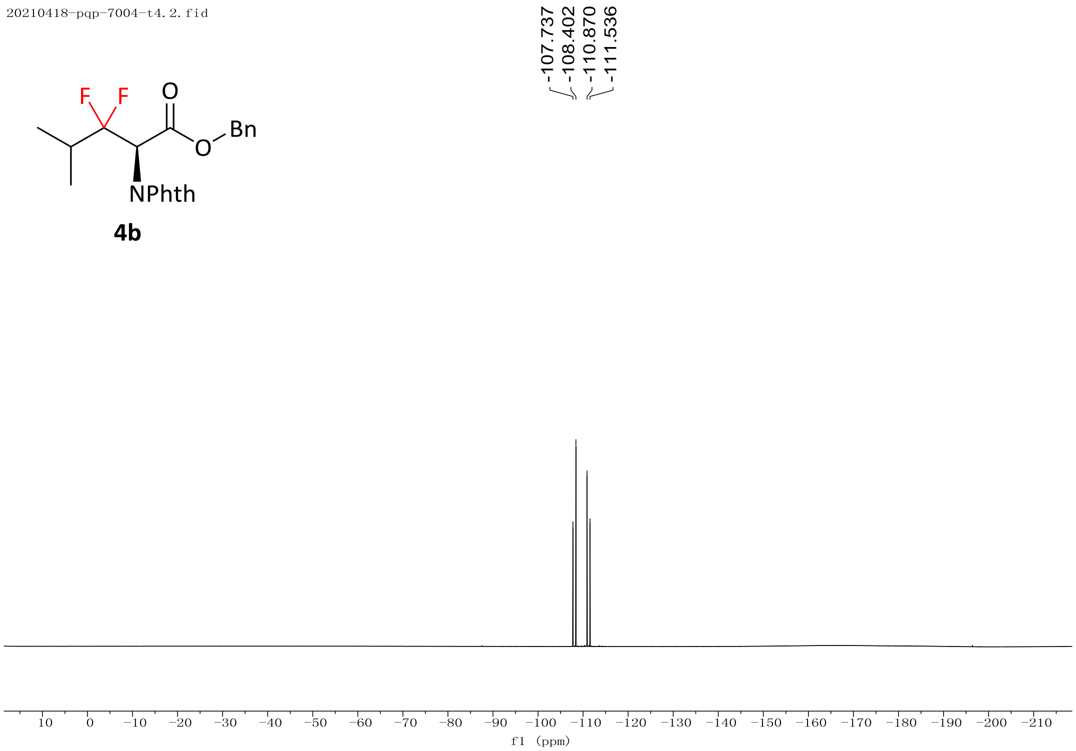

Supplementary Figure 72. ^{1}H NMR Spectra of **4b**

5

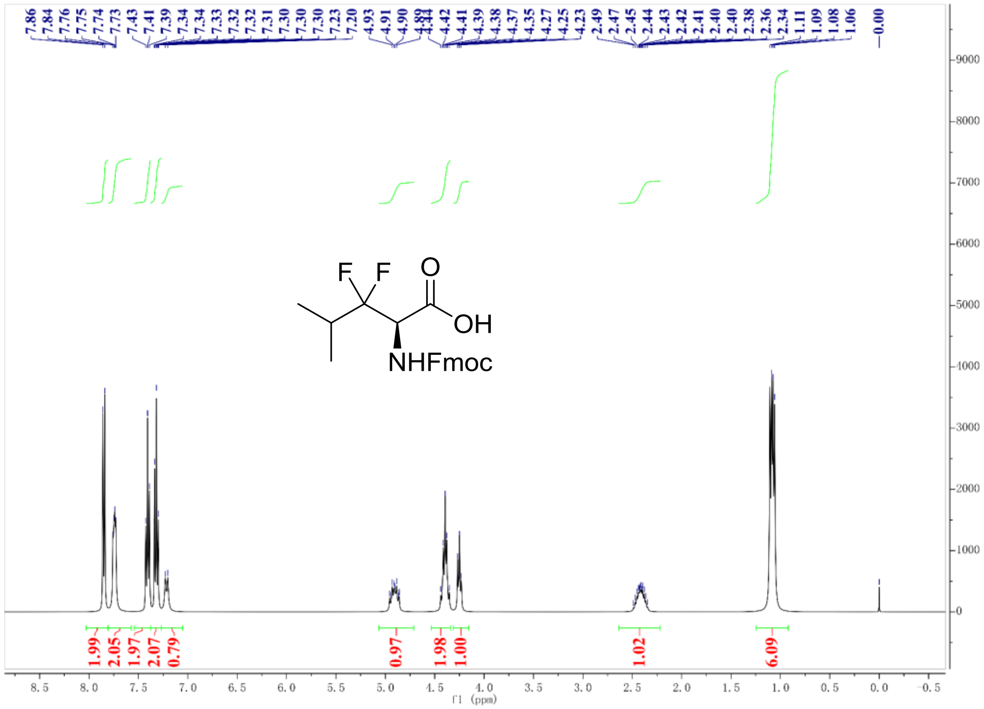

Supplementary Figure 73. ^{1}H NMR Spectra of **5**

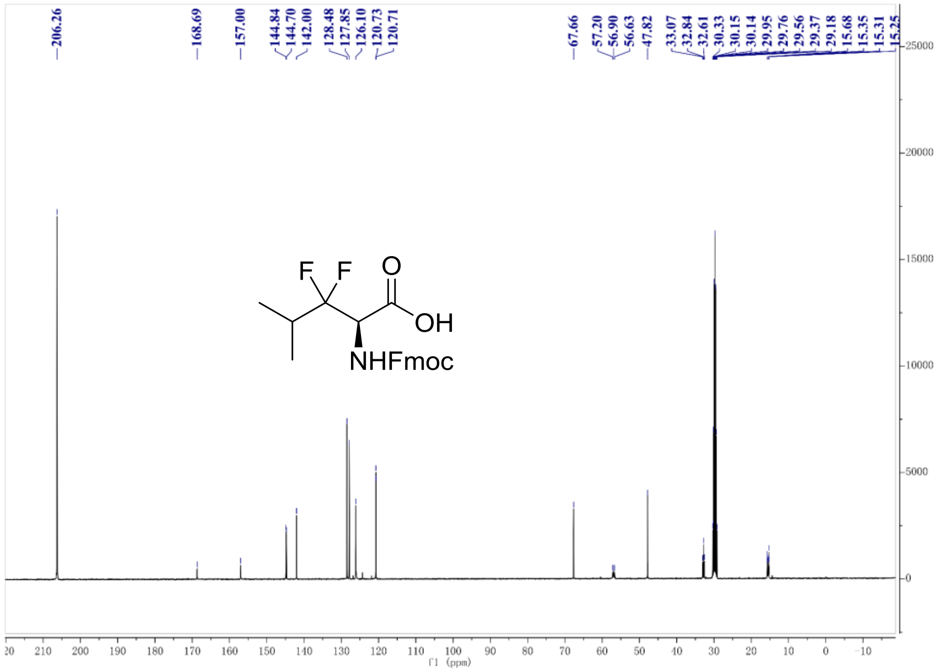

Supplementary Figure 74. ^{13}F NMR Spectra of **5**

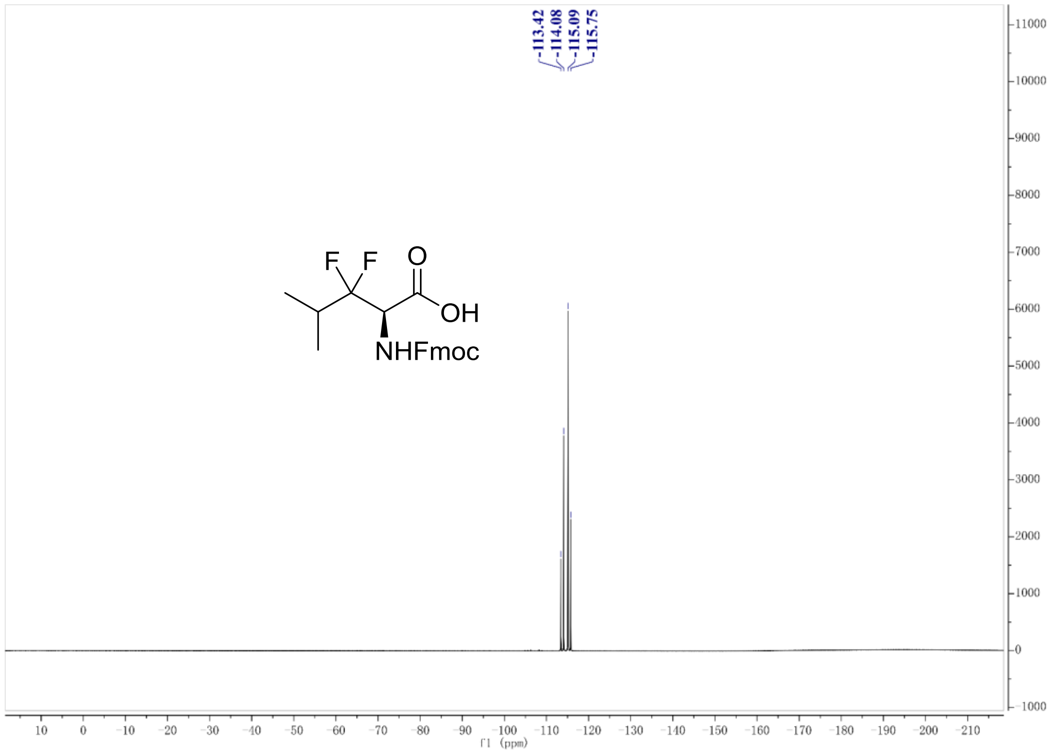

Supplementary Figure 75. ^{19}F NMR Spectra of **5**

HPLC Spectra

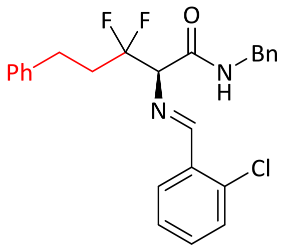

2a

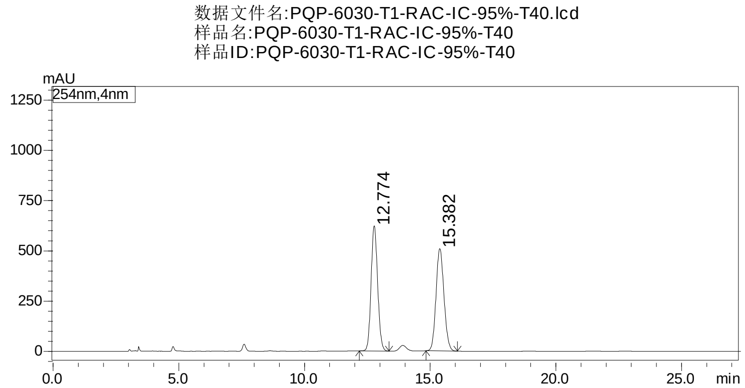

Peak	Ret. Time	Area	Height	Area%	Height%
1	12.774	11282321	623072	50.148	55.005
2	15.382	11215603	509681	49.852	44.995
Total		22497924	1132753	100.000	100.000

Supplementary Figure 76. HPLC spectrum of racemic **2a**

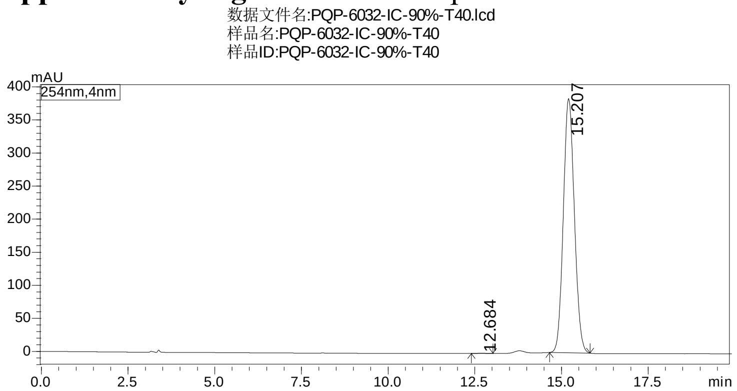

Peak	Ret. Time	Area	Height	Area%	Height%
1	12.684	6587	400	0.077	0.104
2	15.207	8497354	384104	99.923	99.896
Total		8503941	384504	100.000	100.000

Supplementary Figure 77. HPLC spectrum of **2a**

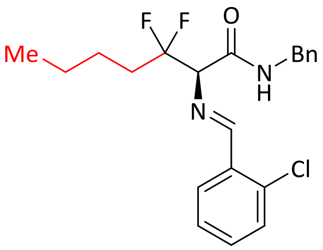

2b

数据文件名:PQP-6107-2-RAC-IC-90%-T40.lcd
样品名:PQP-6107-2-RAC-IC-90%-T40
样品ID:PQP-6107-2-RAC-IC-90%-T40

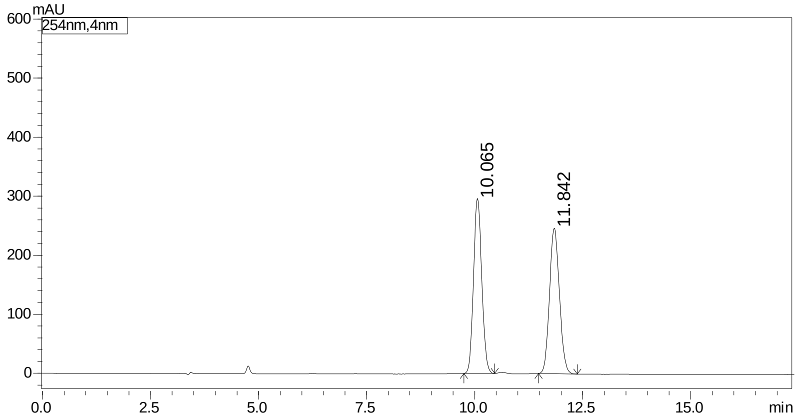

Peak	Ret. Time	Area	Height	Area%	Height%
1	10.065	3938655	296136	49.994	54.567
2	11.842	3939596	246564	50.006	45.433
Total		7878251	542699	100.000	100.000

Supplementary Figure 78. HPLC spectrum of racemic **2b**

数据文件名:PQP-6107-1-chiral-IC-90%-T40.lcd
样品名:PQP-6107-1-chiral-IC-90%-T40
样品ID:PQP-6107-1-chiral-I C-90%-T40

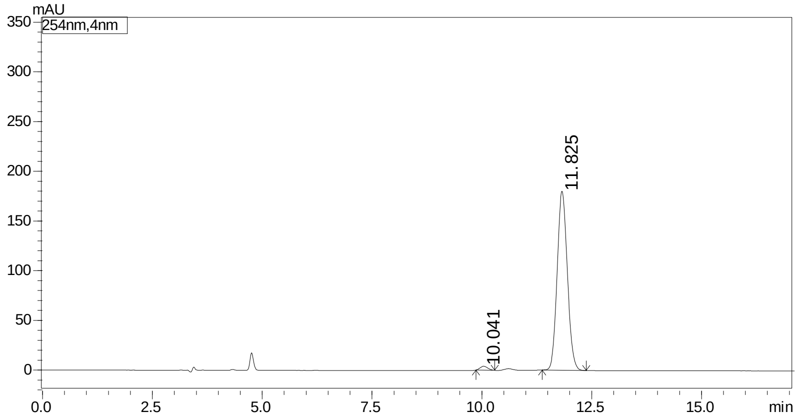

Peak	Ret. Time	Area	Height	Area%	Height%
1	10.041	47665	3925	1.609	2.133
2	11.825	2914416	180066	98.391	97.867
Total		2962081	183991	100.000	100.000

Supplementary Figure 79. HPLC spectrum of **2b**

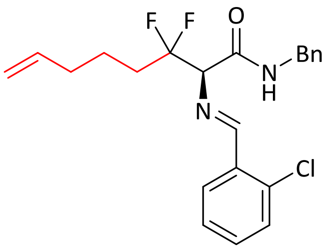

2c

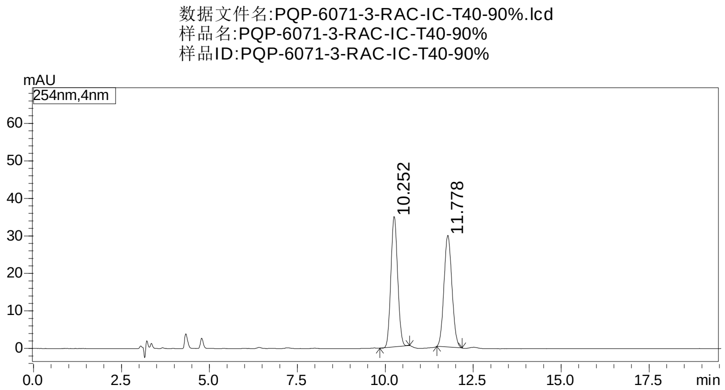

Peak	Ret. Time	Area	Height	Area%	Height%
1	10.252	465877	34778	49.828	53.873
2	11.778	469088	29778	50.172	46.127
Total		934965	64557	100.000	100.000

Supplementary Figure 80. HPLC spectrum of racemic **2c**

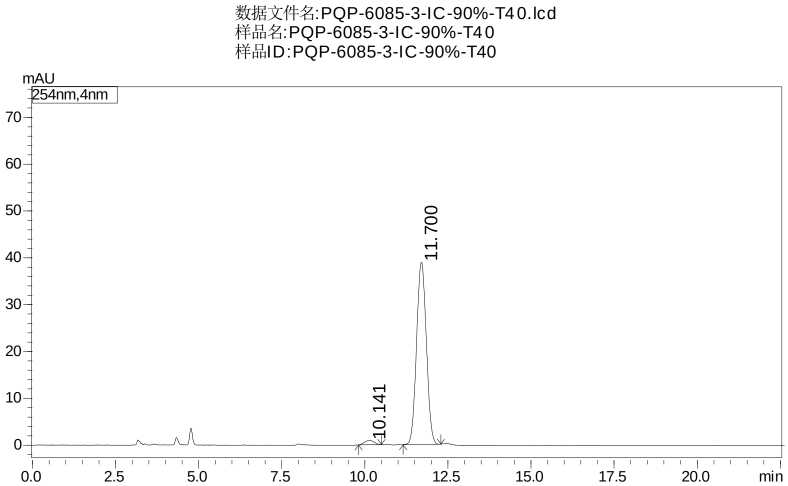

Peak	Ret. Time	Area	Height	Area%	Height%
1	10.141	18310	917	2.242	2.299
2	11.700	798449	38944	97.758	97.701
Total		816759	39861	100.000	100.000

Supplementary Figure 81. HPLC spectrum of **2c**

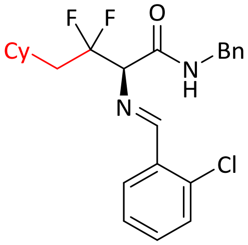

2d

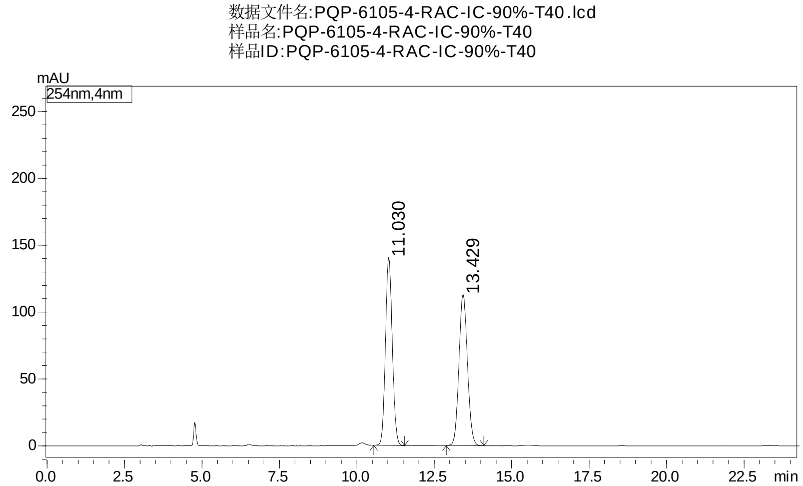

Peak	Ret. Time	Area	Height	Area%	Height%
1	11.030	2152530	140466	49.997	55.444
2	13.429	2152782	112884	50.003	44.556
Total		4305312	253351	100.000	100.000

Supplementary Figure 82. HPLC spectrum of racemic **2d**

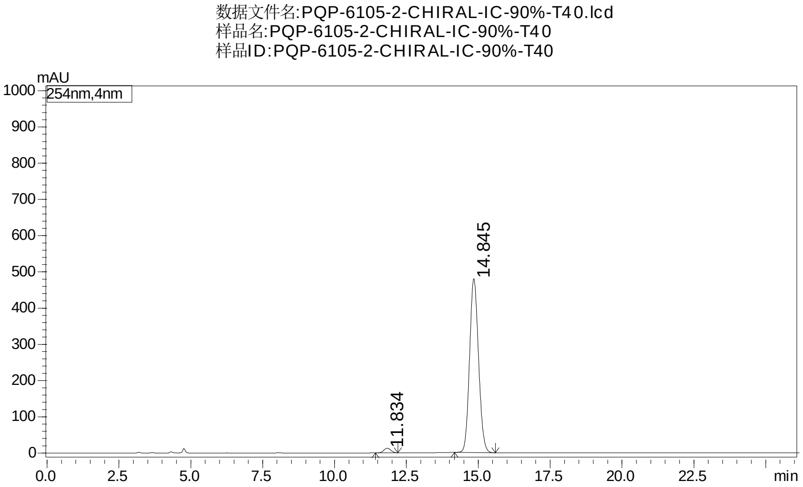

Peak	Ret. Time	Area	Height	Area%	Height%
1	11.834	223351	12874	2.101	2.614
2	14.845	10408062	479545	97.899	97.386
Total		10631413	492419	100.000	100.000

Supplementary Figure 83. HPLC spectrum of **2d**

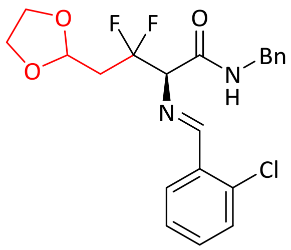

2e

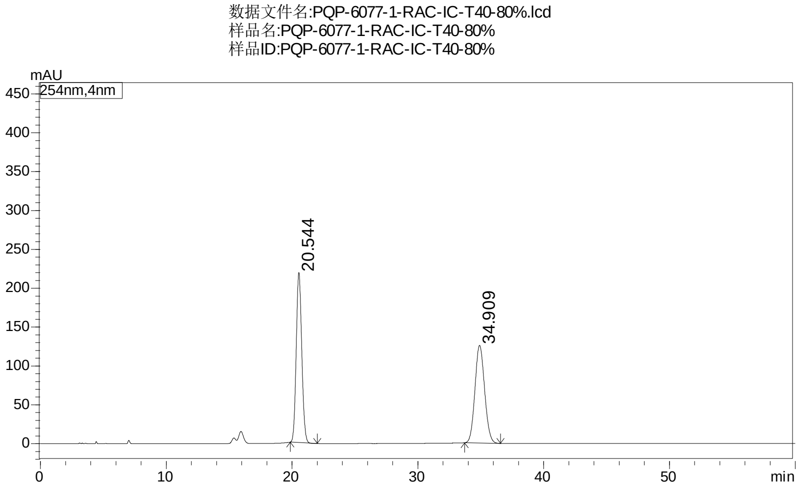

Peak	Ret. Time	Area	Height	Area%	Height%
1	20.544	6689456	218969	49.956	63.537
2	34.909	6701309	125662	50.044	36.463
Total		13390765	344631	100.000	100.000

Supplementary Figure 84. HPLC spectrum of racemic **2e**

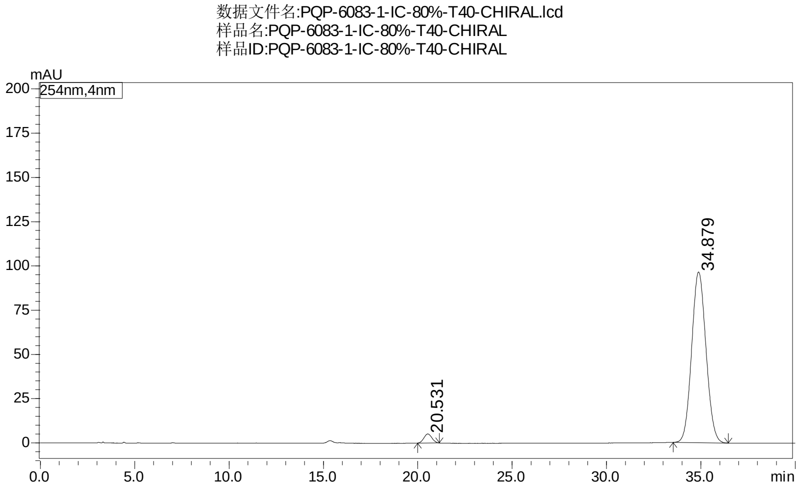

Peak	Ret. Time	Area	Height	Area%	Height%
1	20.531	151041	5138	2.875	5.056
2	34.879	5102097	96492	97.125	94.944
Total		5253139	101630	100.000	100.000

Supplementary Figure 85. HPLC spectrum of **2e**

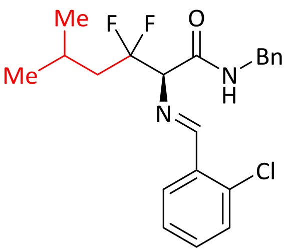

2f

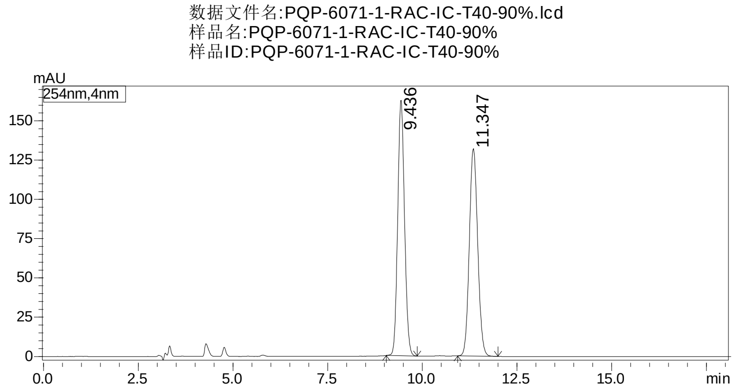

Peak	Ret. Time	Area	Height	Area%	Height%
1	9.436	2042772	162637	50.182	55.179
2	11.347	2027928	132107	49.818	44.821
Total		4070699	294744	100.000	100.000

Supplementary Figure 86. HPLC spectrum of racemic **2f**

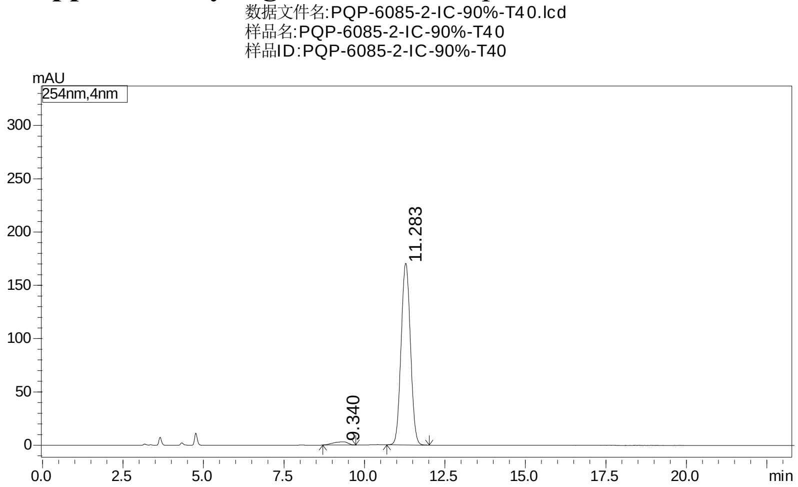

Peak	Ret. Time	Area	Height	Area%	Height%
1	9.340	96665	3057	2.738	1.761
2	11.283	3433445	170484	97.262	98.239
Total		3530110	173541	100.000	100.000

Supplementary Figure 87. HPLC spectrum of **2f**

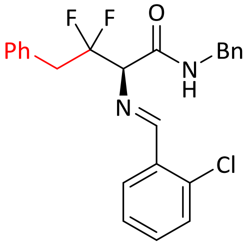

2g

数据文件名:PQP-6113-2-RAC-IC-90%-T40.lcd
样品名:PQP-6113-2-RAC-IC-90%-T40
样品ID:PQP-6113-2-RAC-IC-90%-T40

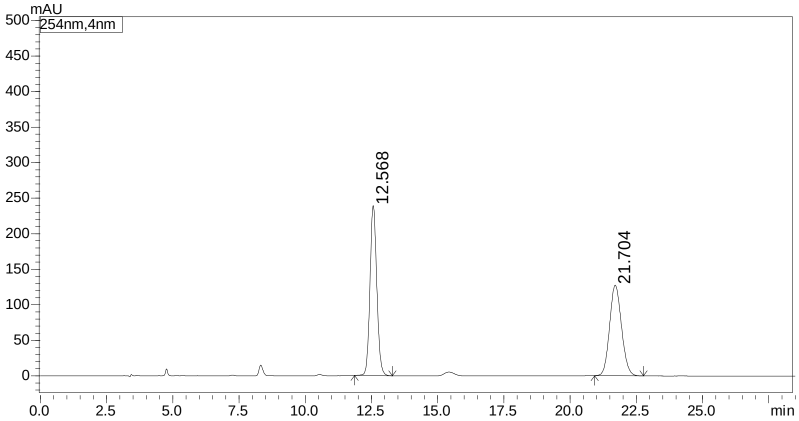

Peak	Ret. Time	Area	Height	Area%	Height%
1	12.568	4279088	239347	51.766	65.265
2	21.704	3987135	127383	48.234	34.735
Total		8266223	366730	100.000	100.000

Supplementary Figure 88. HPLC spectrum of racemic **2g**

数据文件名:PQP-6113-1-CHIRAL-IC-90-T40-RE-2.lcd
样品名:PQP-6113-1-CHIRAL-IC-90-T40
样品ID:PQP-6113-1-CHIRAL-IC-90-T40

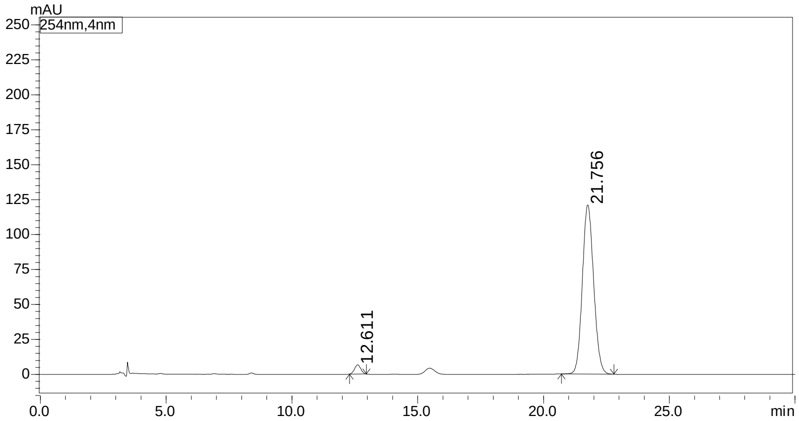

Peak	Ret. Time	Area	Height	Area%	Height%
1	12.611	114166	6584	2.930	5.161
2	21.756	3782013	120975	97.070	94.839
Total		3896179	127558	100.000	100.000

Supplementary Figure 89. HPLC spectrum of **2g**

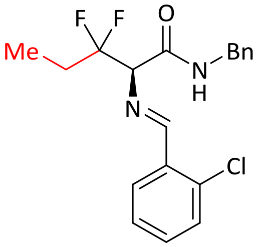

2h

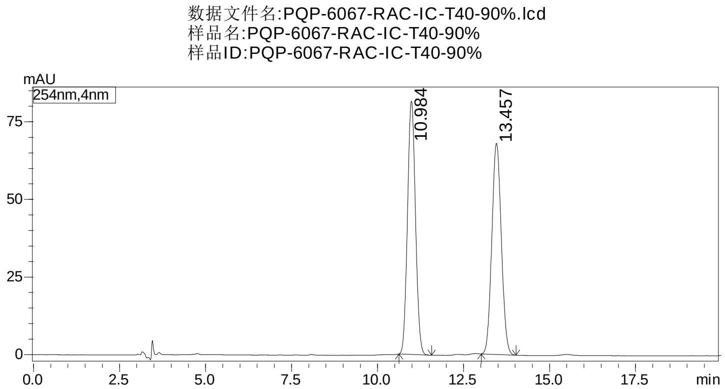

Peak	Ret. Time	Area	Height	Area%	Height%
1	10.984	1312867	81538	50.104	54.514
2	13.457	1307417	68036	49.896	45.486
Total		2620285	149574	100.000	100.000

Supplementary Figure 90. HPLC spectrum of racemic **2h**

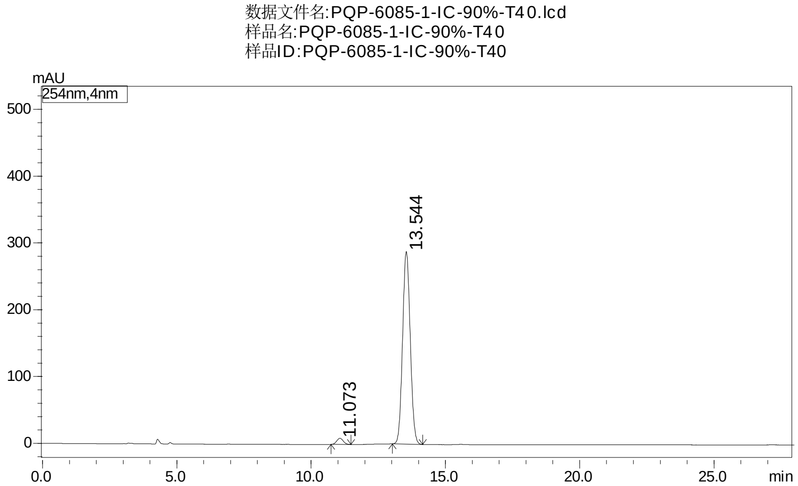

Peak	Ret. Time	Area	Height	Area%	Height%
1	11.073	155890	9151	2.658	3.077
2	13.544	5709558	288256	97.342	96.923
Total		5865449	297407	100.000	100.000

Supplementary Figure 91. HPLC spectrum of **2h**

2i

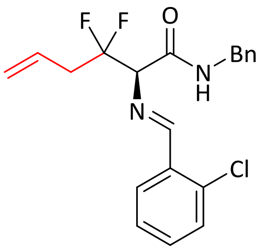

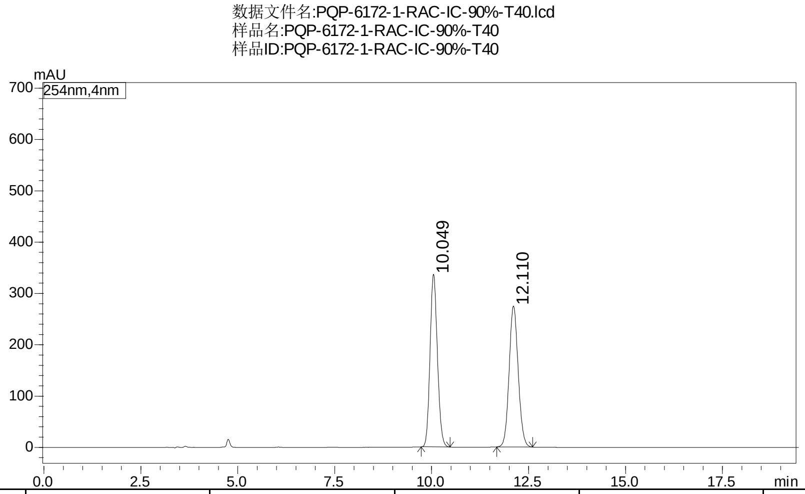

Peak	Ret. Time	Area	Height	Area%	Height%
1	10.049	4429775	336340	49.742	55.017
2	12.110	4475676	274999	50.258	44.983
Total		8905451	611339	100.000	100.000

Supplementary Figure 92. HPLC spectrum of racemic **2i**

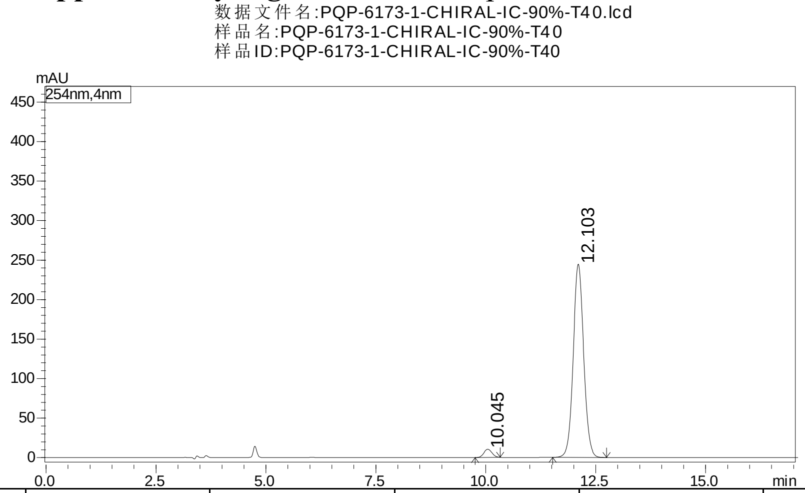

Peak	Ret. Time	Area	Height	Area%	Height%
1	10.045	134120	10399	3.201	4.078
2	12.103	4055551	244582	96.799	95.922
Total		4189671	254981	100.000	100.000

Supplementary Figure 93. HPLC spectrum of **2i**

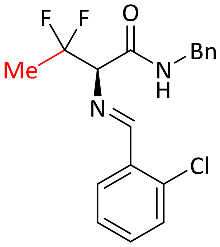

2j

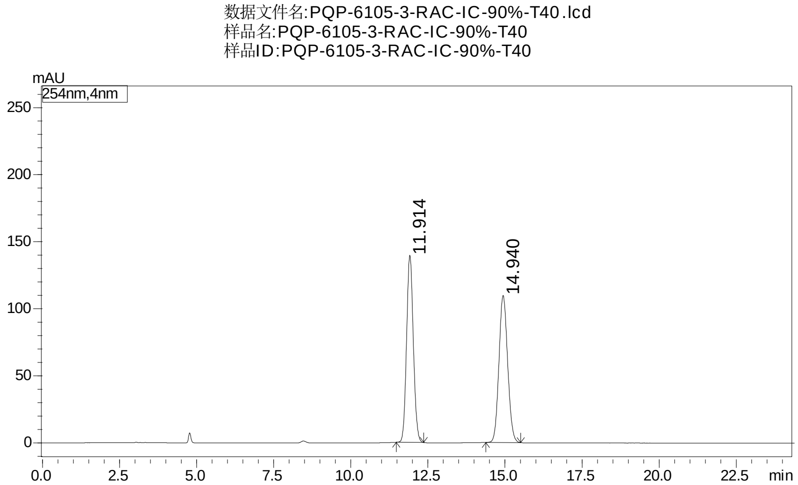

Peak	Ret. Time	Area	Height	Area%	Height%
1	11.914	2125173	139645	49.672	56.003
2	14.940	2153253	109709	50.328	43.997
Total		4278425	249353	100.000	100.000

Supplementary Figure 94. HPLC spectrum of racemic **2j**

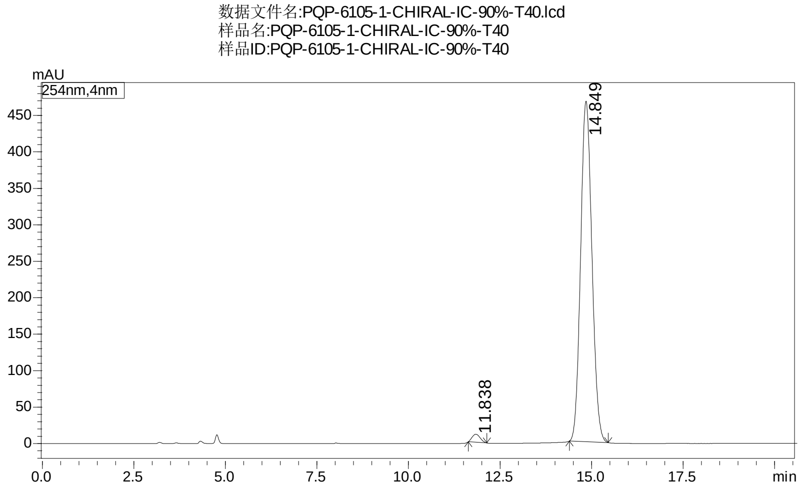

Peak	Ret. Time	Area	Height	Area%	Height%
1	11.838	164596	10928	1.615	2.285
2	14.849	10029342	467227	98.385	97.715
Total		10193938	478155	100.000	100.000

Supplementary Figure 95. HPLC spectrum of **2j**

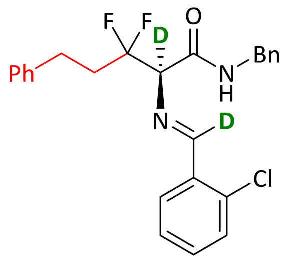

2k

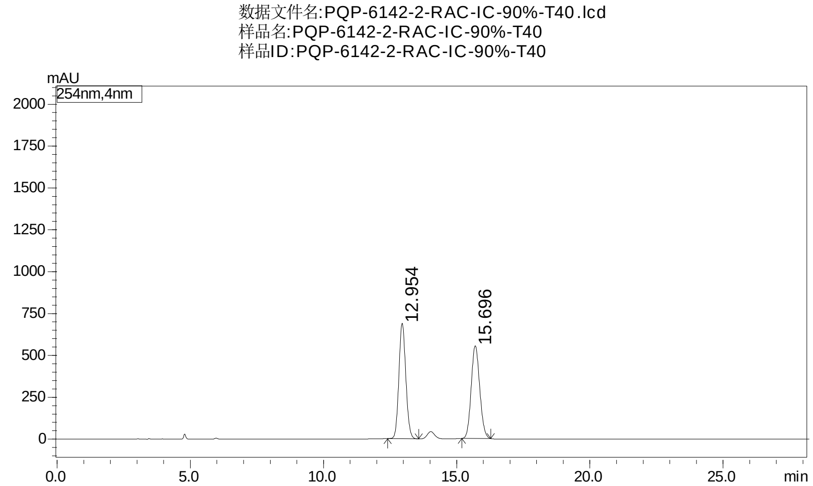

Peak	Ret. Time	Area	Height	Area%	Height%
1	12.954	12489231	689884	50.280	55.462
2	15.696	12349920	554004	49.720	44.538
Total		24839151	1243888	100.000	100.000

Supplementary Figure 95. HPLC spectrum of racemic **2k**

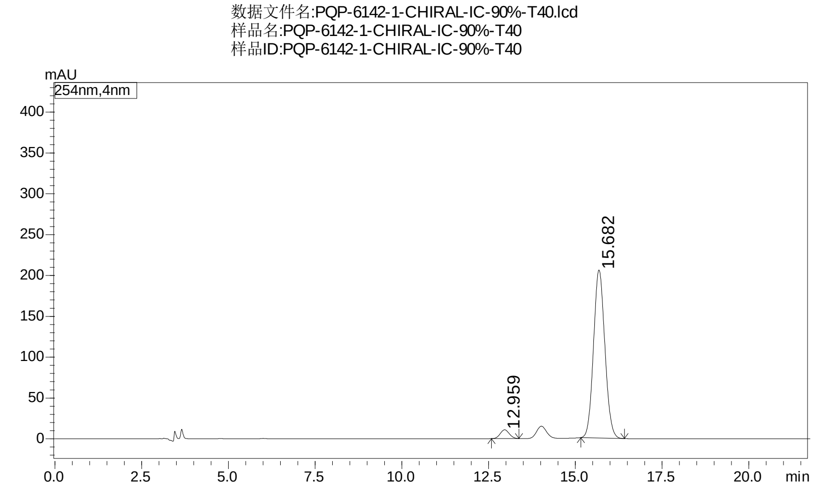

Peak	Ret. Time	Area	Height	Area%	Height%
1	12.959	199043	10850	4.074	5.008
2	15.682	4686601	205786	95.926	94.992
Total		4885644	216636	100.000	100.000

Supplementary Figure 96. HPLC spectrum of **2k**

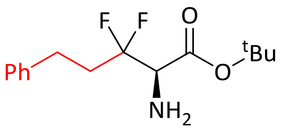

2l

数据文件名:PQP-6023-1-RAC-IC-95%-T40.lcd
样品名:PQP-6023-1-RAC-IC-95%-T40
样品ID:PQP-6023-1-RAC-IC-95%-T40

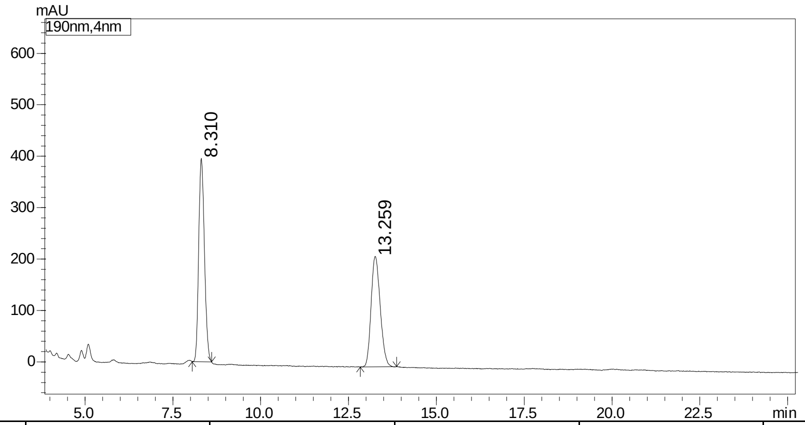

Peak	Ret. Time	Area	Height	Area%	Height%
1	8.310	4498013	395687	53.814	64.857
2	13.259	3860433	214407	46.186	35.143
Total		8358446	610094	100.000	100.000

Supplementary Figure 97. HPLC spectrum of racemic **2l**

数据文件名:PQP-6023-1-chiral-IC-95%-T40.lcd
样品名:PQP-6023-1-chiral-IC-95%-T40
样品ID:PQP-6023-1-chiral-I C-95%-T40

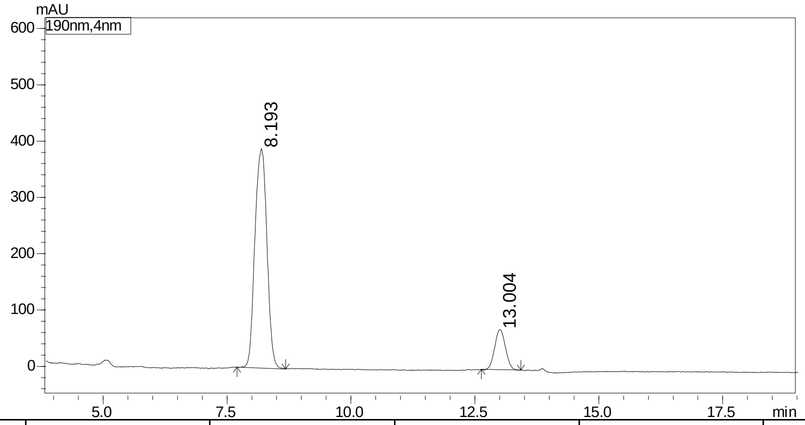

Peak	Ret. Time	Area	Height	Area%	Height%
1	8.193	6576533	389843	85.983	84.522
2	13.004	1072093	71389	14.017	15.478
Total		7648626	461232	100.000	100.000

Supplementary Figure 98. HPLC spectrum of **2l**

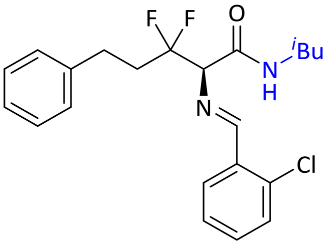

2m

数据文件名:PQP-6044-RAC-IC-90%-T40.lcd
样品名:PQP-6044-RAC-IC-90%-T40
样品ID:PQP-6044-RAC-IC-90%-T40

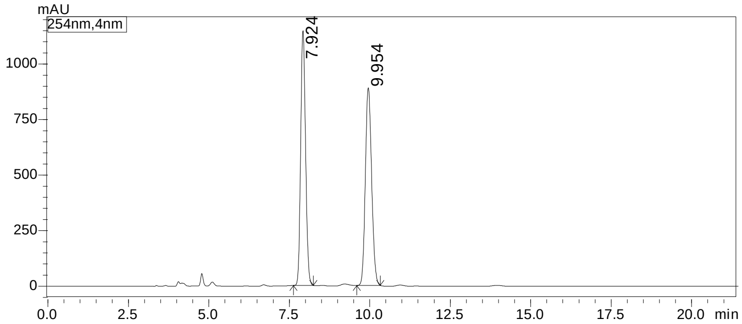

Peak	Ret. Time	Area	Height	Area%	Height%
1	7.924	12103263	1145740	49.970	56.267
2	9.954	12117910	890509	50.030	43.733
Total		24221173	2036249	100.000	100.000

Supplementary Figure 99. HPLC spectrum of racemic **2m**

数据文件名:PQP-6045-Chiral-IC-90%-T40.lcd
样品名:PQP-6045-Chiral-IC-90%-T40
样品ID:PQP-6045-Chiral-IC-90%-T40

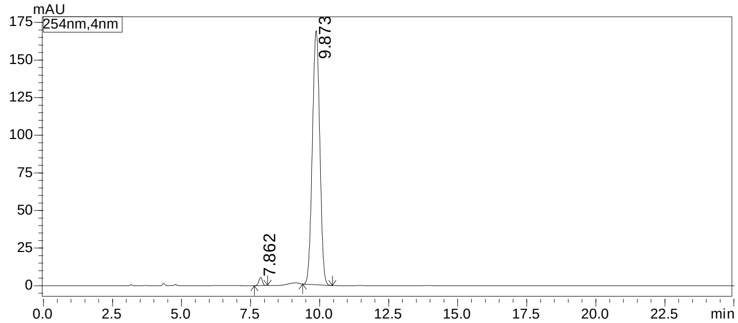

Peak	Ret. Time	Area	Height	Area%	Height%
1	7.862	47396	5389	1.470	3.093
2	9.873	3175992	168852	98.530	96.907
Total		3223389	174240	100.000	100.000

Supplementary Figure 100. HPLC spectrum of **2m**

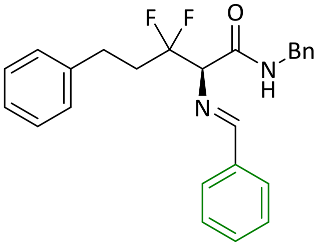

2p

数据文件名:PQP-6071-4-RAC-IC-T40-90%.lcd
样品名:PQP-6071-4-RAC-IC-T40-90%
样品ID:PQP-6071-4-RAC-IC-T40-90%

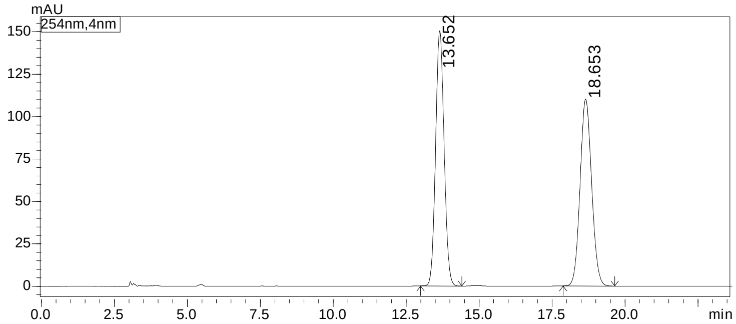

Peak	Ret. Time	Area	Height	Area%	Height%
1	13.652	3045490	150382	50.022	57.724
2	18.653	3042853	110136	49.978	42.276
Total		6088342	260518	100.000	100.000

Supplementary Figure 101. HPLC spectrum of racemic **2p**

数据文件名:PQP-6103-1-RE-CHIRAL-IC-90%-T40.lcd
样品名:PQP-6103-1-CHIRAL-IC-90%-T40
样品ID:PQP-6103-1-CHIRAL-IC-90%-T40

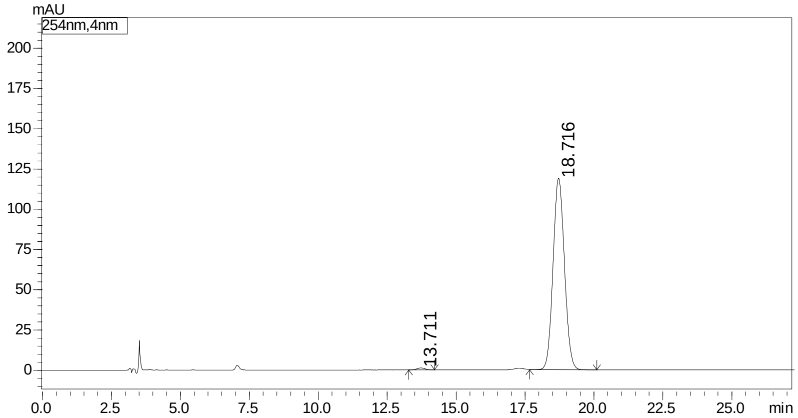

Peak	Ret. Time	Area	Height	Area%	Height%
1	13.711	24279	1190	0.705	0.992
2	18.716	3418449	118818	99.295	99.008
Total		3442728	120008	100.000	100.000

Supplementary Figure 102. HPLC spectrum of **2p**

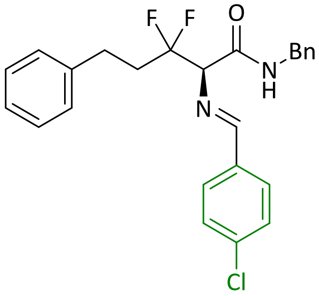

2q

数据文件名:PQP-6071-5-RAC-IC-T40-90%.lcd
样品名:PQP-6071-5-RAC-IC-T40-90%
样品ID:PQP-6071-5-RAC-IC-T40-90%

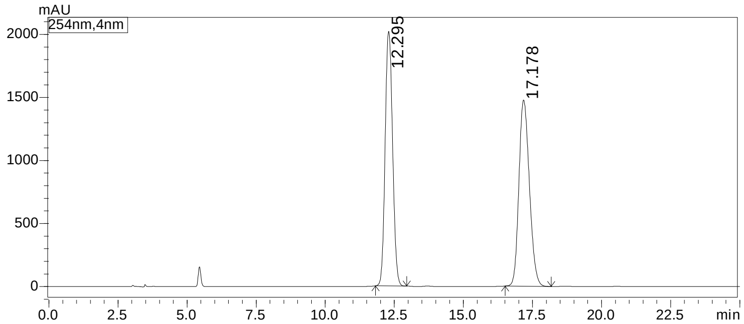

Peak	Ret. Time	Area	Height	Area%	Height%
1	12.295	37731584	2017938	49.157	57.762
2	17.178	39025385	1475578	50.843	42.238
Total		76756969	3493516	100.000	100.000

Supplementary Figure 103. HPLC spectrum of racemic **2q**

数据文件名:PQP-6103-2-CHIRAL-IC-90%-T40.lcd
样品名:PQP-6103-2-CHIRAL-IC-90%-T40
样品ID:PQP-6103-2-CHIRAL-IC-90%-T40

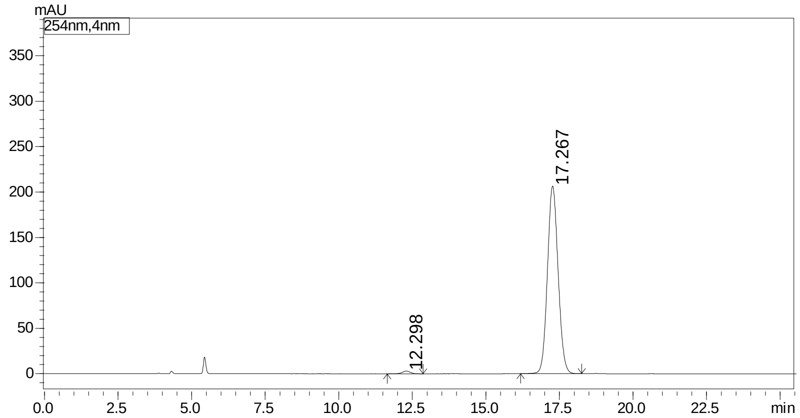

Peak	Ret. Time	Area	Height	Area%	Height%
1	12.298	55661	2955	1.054	1.410
2	17.267	5225388	206654	98.946	98.590
Total		5281050	209609	100.000	100.000

Supplementary Figure 104. HPLC spectrum of **2q**

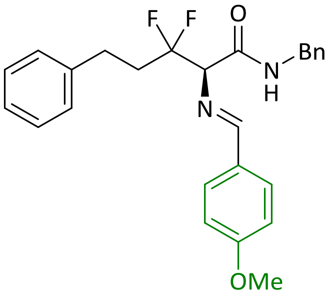

2r

数据文件名:PQP-6077-2-RAC-IC-T40-80%.lcd
样品名:PQP-6077-2-RAC-IC-T40-80%
样品ID:PQP-6077-2-RAC-IC-T40-80%

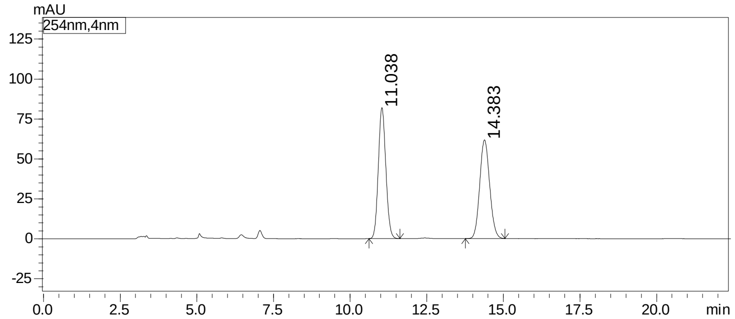

Peak	Ret. Time	Area	Height	Area%	Height%
1	11.038	1397767	81901	50.074	57.000
2	14.383	1393626	61785	49.926	43.000
Total		2791394	143686	100.000	100.000

Supplementary Figure 105. HPLC spectrum of racemic **2r**

数据文件名:PQP-6083-2-IC-80%-T40-CHIRAL.lcd
样品名:PQP-6083-2-IC-80%-T40-CHIRAL
样品ID:PQP-6083-2-IC-80%-T40-CHIRAL

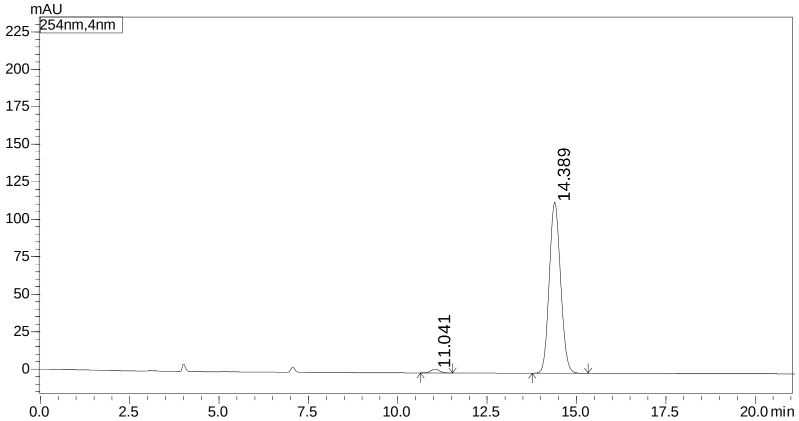

Peak	Ret. Time	Area	Height	Area%	Height%
1	11.041	42531	2545	1.662	2.183
2	14.389	2516863	114008	98.338	97.817
Total		2559394	116553	100.000	100.000

Supplementary Figure 106. HPLC spectrum of **2r**

2s

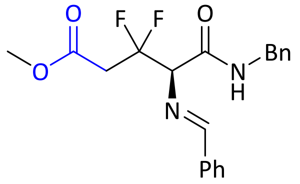

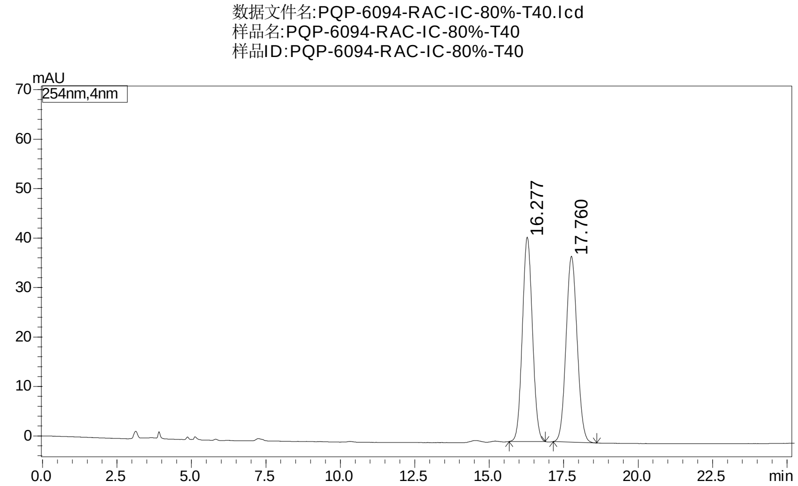

Peak	Ret. Time	Area	Height	Area%	Height%
1	16.277	973000	41342	49.921	52.367
2	17.760	976083	37604	50.079	47.633
Total		1949084	78946	100.000	100.000

Supplementary Figure 107. HPLC spectrum of racemic **2s**

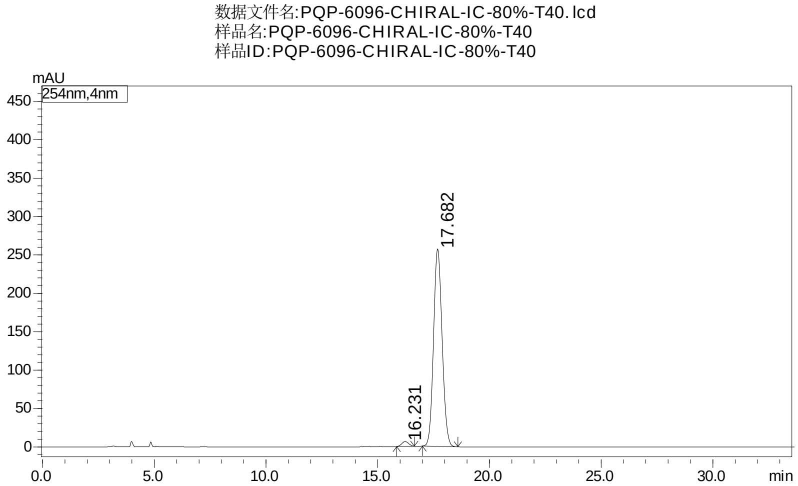

Peak	Ret. Time	Area	Height	Area%	Height%
1	16.231	143049	6466	2.061	2.454
2	17.682	6798519	257076	97.939	97.546
Total		6941568	263542	100.000	100.000

Supplementary Figure 108. HPLC spectrum of **2s**

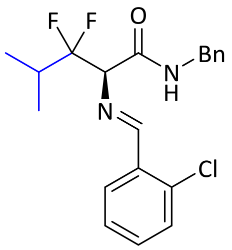

2t

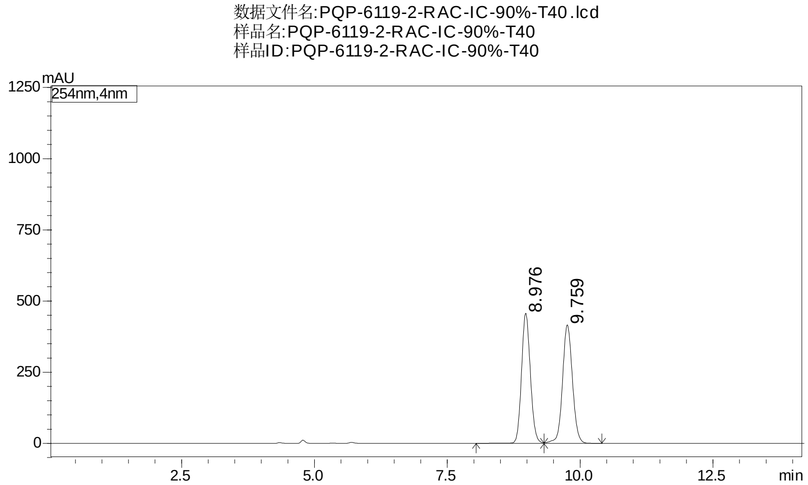

Peak	Ret. Time	Area	Height	Area%	Height%
1	8.976	5305725	456631	49.492	52.303
2	9.759	5414737	416414	50.508	47.697
Total		10720461	873044	100.000	100.000

Supplementary Figure 109. HPLC spectrum of racemic **2t**

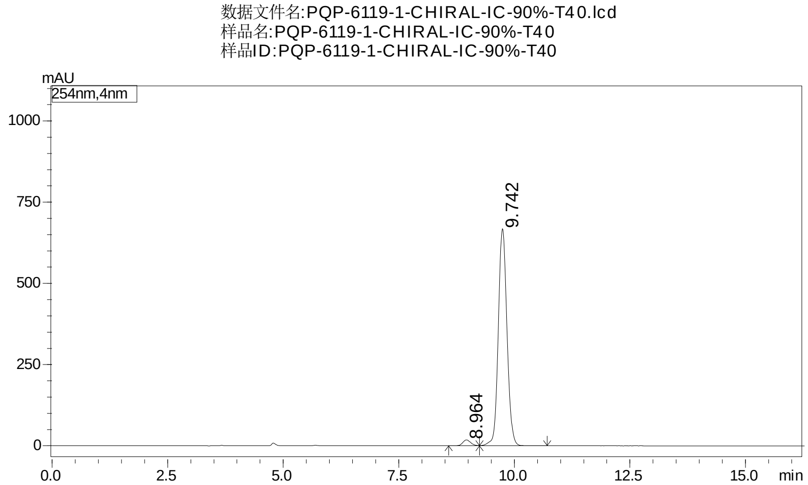

Peak	Ret. Time	Area	Height	Area%	Height%
1	8.964	219203	18153	2.451	2.645
2	9.742	8725655	668202	97.549	97.355
Total		8944857	686355	100.000	100.000

Supplementary Figure 110. HPLC spectrum of **2t**

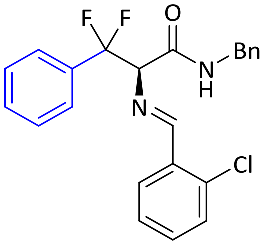

2u

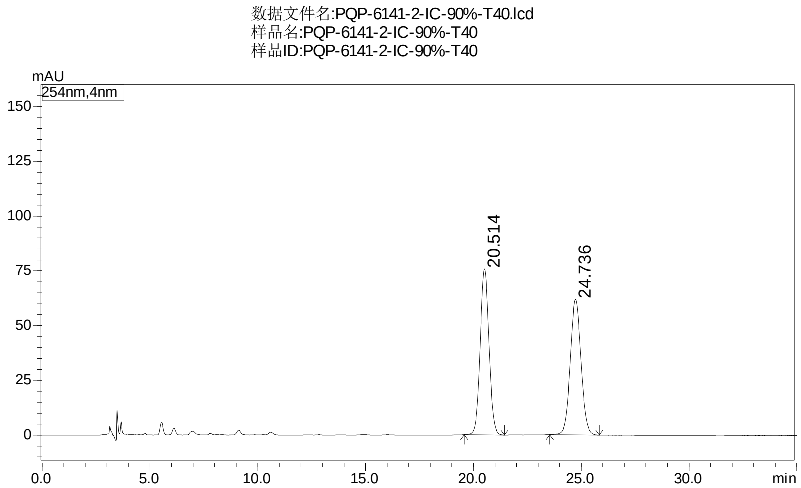

Peak	Ret. Time	Area	Height	Area%	Height%
1	20.514	2161966	75787	50.146	55.044
2	24.736	2149337	61897	49.854	44.956
Total		4311303	137684	100.000	100.000

Supplementary Figure 111. HPLC spectrum of racemic **2u**

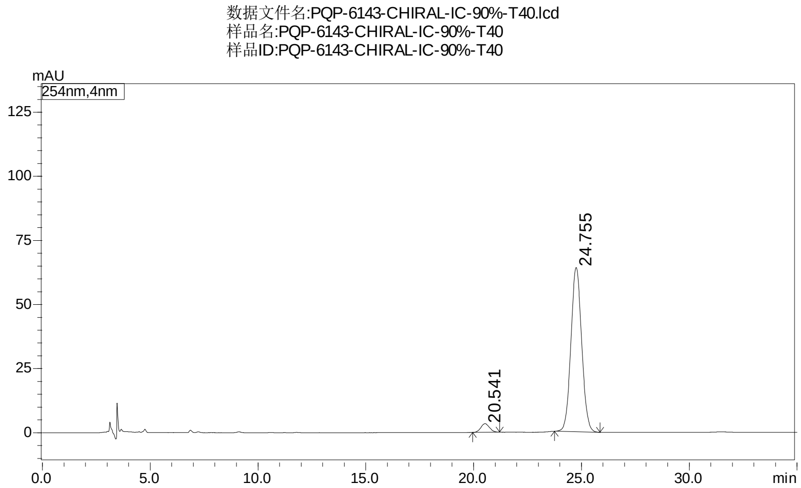

Peak	Ret. Time	Area	Height	Area%	Height%
1	20.541	93982	3346	4.050	4.962
2	24.755	2226380	64087	95.950	95.038
Total		2320362	67433	100.000	100.000

Supplementary Figure 112. HPLC spectrum of **2u**

4a

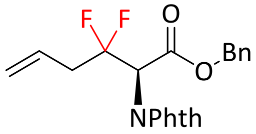

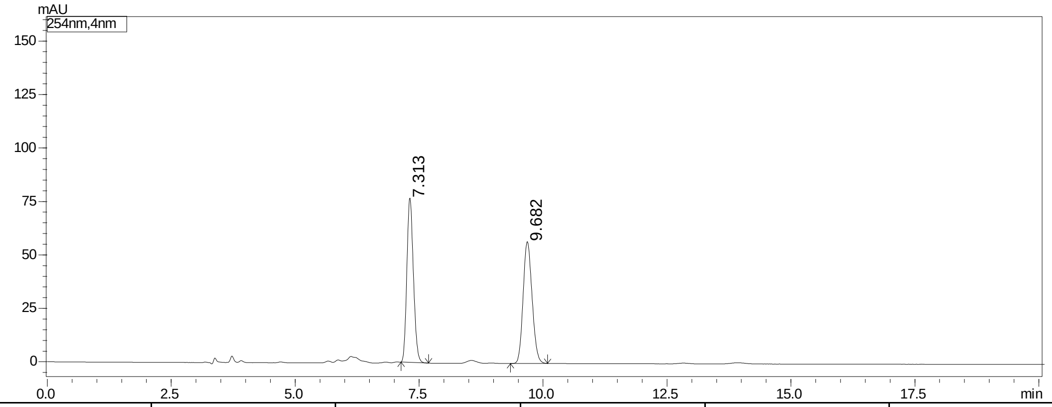

Peak	Ret. Time	Area	Height	Area%	Height%
1	7.313	707078	76790	49.656	57.409
2	9.682	716863	56970	50.344	42.591
Total		1423940	133760	100.000	100.000

Supplementary Figure 113. HPLC spectrum of racemic **4a**

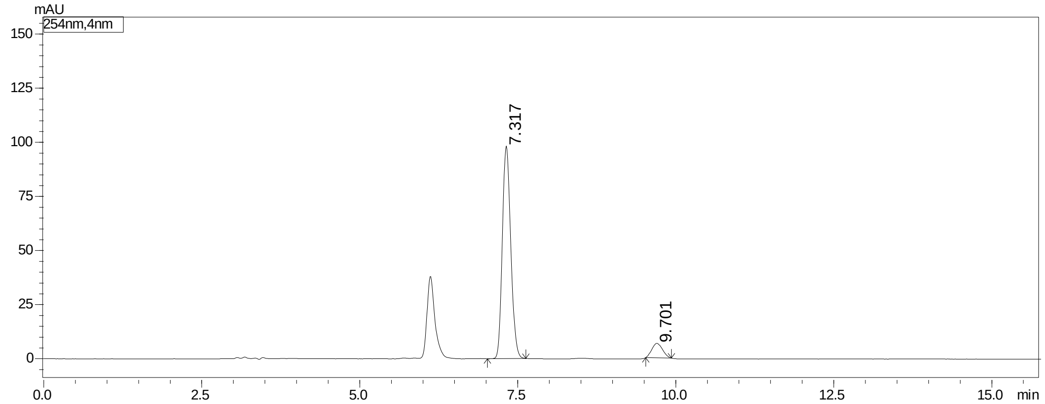

Peak	Ret. Time	Area	Height	Area%	Height%
1	7.317	908325	98200	92.066	93.678
2	9.701	78279	6627	7.934	6.322
Total		986603	104827	100.000	100.000

Supplementary Figure 114. HPLC spectrum of **4a**

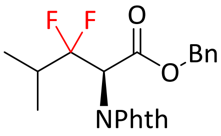

4b

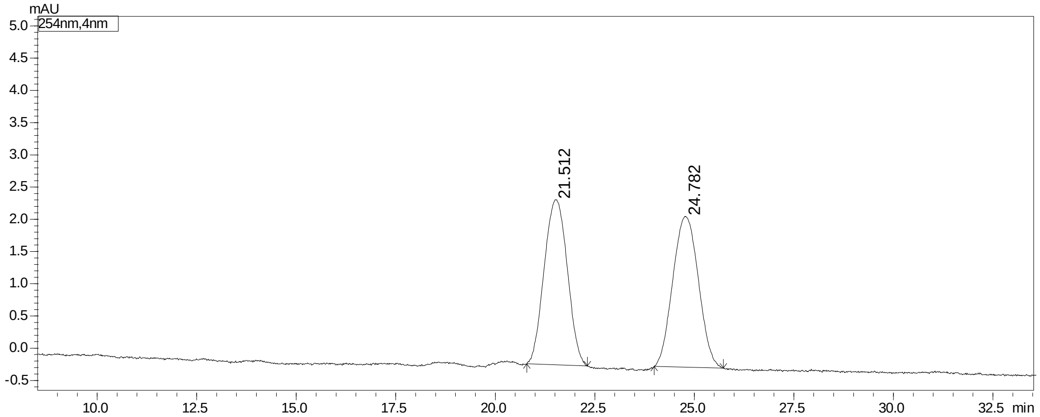

Peak	Ret. Time	Area	Height	Area%	Height%
1	21.512	103775	2561	49.714	52.249
2	24.782	104970	2341	50.286	47.751
Total		208745	4902	100.000	100.000

Supplementary Figure 115. HPLC spectrum of racemic **4b**

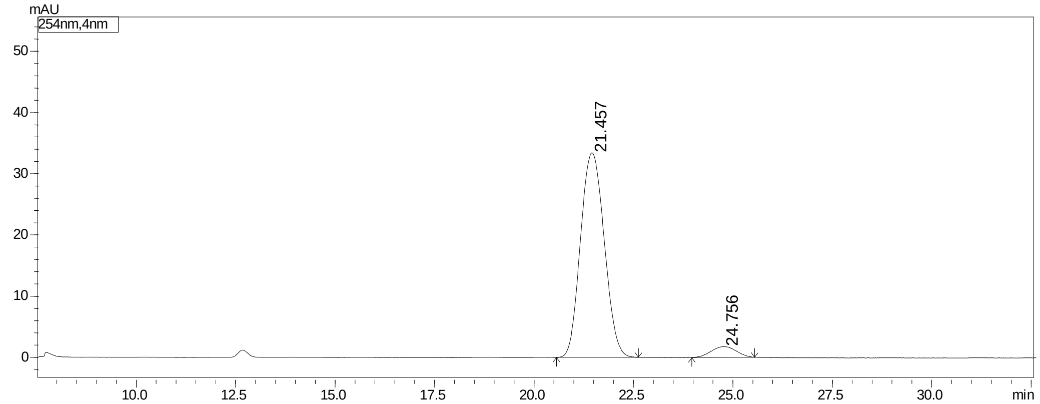

Peak	Ret. Time	Area	Height	Area%	Height%
1	21.457	1389247	33420	94.719	94.985
2	24.756	77463	1765	5.281	5.015
Total		1466710	35185	100.000	100.000

Supplementary Figure 116. HPLC spectrum of **4b**

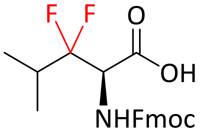

5

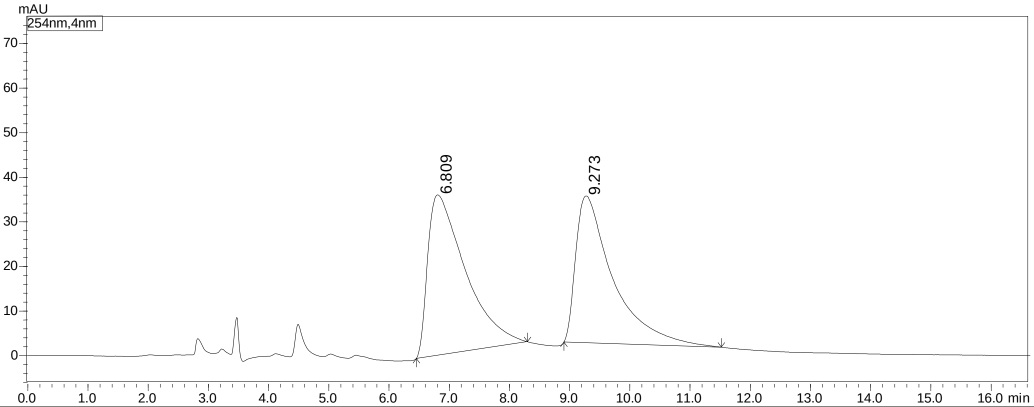

Peak	Ret. Time	Area	Height	Area%	Height%
1	6.809	1561155	35940	52.476	52.199
2	9.273	1413848	32912	47.524	47.801
Total		2975003	68852	100.000	100.000

Supplementary Figure 117. HPLC spectrum of racemic **5**

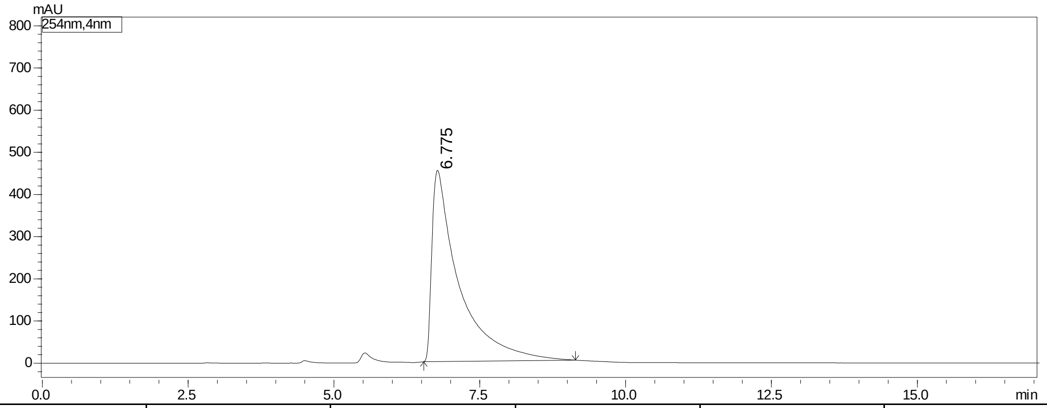

Peak	Ret. Time	Area	Height	Area%	Height%
1	6.775	14596798	453464	100.000	100.000
Total		14596798	453464	100.000	100.000

Supplementary Figure 118. HPLC spectrum of **5**

Supplementary Methods

General Information

Chemicals were purchased from Energy Chemical, J & K Scientific, Adamas-beta, and Sigma-Aldrich Co., used as received. Solvents were dried on alumina columns using a solvent dispensing system. Thin-layer chromatography (TLC) was conducted on plates (GF254) supplied by Yantai Chemicals (China) and visualized using a combination of UV, anisaldehyde, iodine, and potassium permanganate staining. ^{1}H NMR, ^{13}C NMR, ^{19}F NMR, spectra were recorded on a Bruker ACF400 (400 MHz) spectrometer. Chemical shifts were reported in parts per million (ppm), and the residual solvent peak was used as an internal reference: proton (chloroform δ 7.26, DMSO-d_6 δ 2.50, Acetone-d_6 δ 2.05), carbon (chloroform δ 77.16, DMSO-d_6 δ 39.52, Acetone-d_6 δ 206.7, 29.9) or tetramethylsilane (TMS δ 0.00) was used as a reference. Multiplicity was indicated as follows: s (singlet), d (doublet), t (triplet), q (quartet), m (multiplet), dd (doublet of doublet), bs (broad singlet). Coupling constants were reported in Hertz (Hz). All high resolution mass spectra were obtained from the Tsinghua University Mass Spectrometry Facility. Flash chromatography separations were performed on Silica gel (300-400 mesh) supplied by SiliaFlash P60. The enantiomeric excesses of products were determined on a Shimadzu LC-20AT Chiral HPLC.

Reagents and materials for peptide synthesis:

Fmoc-Gly-Wang resin and H-Gly-2-ClTrt resin were purchased from Iris (Marktredwitz, Germany) and Merck (Darmstadt, Germany), respectively. Fmoc-amino acids including Fmoc-L-Cys(Trt)-OH, Fmoc-L-Ile-OH, Fmoc-L-Leu-OH, Fmoc-L-Asn(Trt)-OH, Fmoc-L-Pro-OH*H_2O, Fmoc-L-Gln(Trt)-OH were purchased from Iris (Marktredwitz, Germany), Fmoc-Tyr(tBu)-OH was purchased from Merck (Darmstadt, Germany). Piperidine, *N,N'*-Diisopropylcarbodiimide (DIC), 1,2-Ethanedithiol (EDT) and Triisopropylsilane (TIPS) were purchased from Sigma-Aldrich (Steinheim, Germany), 1-Hydroxybenzotriazole hydrate (HOBt) was purchased from J&K Scientific GmbH, (Pforzheim, Germany), *N,N,N',N'*-tetramethyl-O-(1H-benzotriazol-1-yl)-uronium hexafluorophosphate (HBTU) was purchased from Iris (Marktredwitz, Germany), *N,N*-Diisopropylethylamine (DIPEA) was purchased from Fisher Scientific (Geel, Belgium), Glutathione (GSH), Glutathione disulfide (GSSG), Tris, and Trifluoroacetic acid (TFA) were purchased from CarlRoth (Karlsruhe, Germany), Dulbecco's Phosphate Buffered Saline (PBS) was purchased from BioWest (Düsseldorf, Germany), Dimethylformamide (DMF, HPLC grade) and Dichloromethane (DCM, Analytical reagent grade) were purchased from Fisher Scientific (Geel, Belgium).

Analytical UPLC-UV/MS:

UPLC-UV/MS traces for peptides were recorded on an ACQUITY H-class instrument (Waters Corporation, Milford, Massachusetts, USA) equipped with a quaternary solvent manager, a Waters auto-sampler, a Waters TUV detector and a Waters Acquity QDa detector with an Acquity UPLC®-BEH C18 1.7μm, 2.1×50mm column RP column with a flow rate of 0.6ml/min (Waters Corp., USA) and using eluents A (99.9% H_2O, 0.1% TFA) and B (99.9% CAN, 0.1% TFA) in the corresponding linear gradient. UPLC-UV chromatograms were recorded at 220nm. The gradient used for all the characterization in this study was 5% to 95% B in 5 min.

Semi-preparative HPLC for purification:

All the peptides were purified by semi-preparative HPLC, performed on a Shimadzu Prominence 20A system (Shimadzu Corporation, Kyoto, Japan) equipped with columns as following details: semi-preparative column – Nucleodur C18 HTec, 5μm, 250×21mm; semi-preparative column – Nucleodur C18 HTec, 5μm, 250×10mm (both of the semi-preparative HPLC columns were purchased from Macherey-Nagel, GmbH & Co. KG, Düren, Germany). Eluents A (99.9% H_2O, 0.1% TFA) and B (9.9% ACN, 20% H_2O, 0.1% TFA) were applied in the corresponding linear gradient. Peak detection was conducted at 220 nm. The column and gradient used for each peptide purification in this study were used as followings: 1. Linear WT Oxytocin: column – Nucleodur C18 HTec, 5μm, 250×21mm; gradient – 30% to 95% B in 60min; 2. Linear difluoro-Oxytocin: column – Nucleodur C18 HTec, 5μm, 250×10mm; gradient – 30% to 95% B in 45min; 3. Folded WT Oxytocin: column – Nucleodur C18 HTec, 5μm, 250×10mm; gradient – 25% to 95% B in 60min; 4. Folded difluoro-Oxytocin: column – Nucleodur C18 HTec, 5μm, 250×10mm; gradient – 20% to 90% B in 60min.

Procedure for Synthesis of Catalyst G

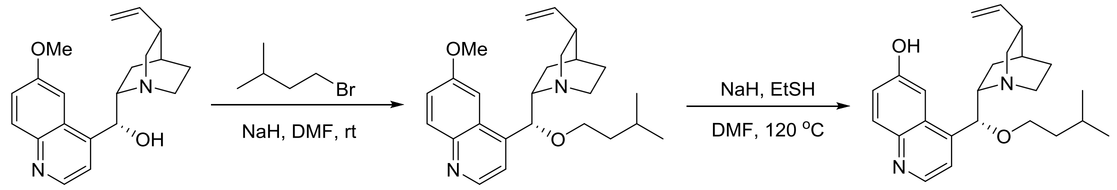

Quinine T1 G

To a solution of quinine (6.5 g, 20 mmol) in dry DMF (60 mL) was added NaH (60% suspension in mineral oil) (2.4 g, 60 mmol) portionwise at rt. After the reaction mixture was stirred at rt for 1 h, 1-bromo-3-methylbutane (6.0 g, 40 mmol) was added dropwise over 10 min. Upon stirring at rt overnight (TLC monitored), the reaction mixture was quenched with brine (10 mL), and the pH value was adjusted with 1N HCl to 1. The mixture was washed with Et_2O (3x40 mL), then the pH value of aqueous layer was adjusted to 9 with ammonia and extracted with EA (3x60 mL). The combined organic layer was washed with brine (3x30 mL), dried over MgSO4, filtered, concentrated. The obtained residue (crude **T1**) was used directly for the next step without further purification.

To a suspension of NaH (60% in mineral oil) (4.0 g, 100 mmol) in dry DMF (50 mL) was added EtSH (12.4 g, 200 mmol) dropwise at 0 ^{o}C under N_2 over 10 min. After the reaction mixture was stirred at rt for 10 min, a solution of compound **T1** (obtained from up step) in DMF (18 mL) was added at rt. Upon stirring at 120 ºC for 12 h, the reaction mixture was acidified with 1N HCl, washed with Et_2O (3x90 mL), brought to pH 9 with ammonia, extracted with EA (5x50 mL). The combined organic layer was washed with brine, dried over $MgSO_4$, filtered, concentrated, and purified by flash chromatography (silical gel, packed with EtOAc containing 10% Et_3N) (eluted with EtOAc/MeOH = 20/1 to 15/1) to give compound **G** as a light yellow foam which was washed with Et_2O to give a white foam (5.4 g, 71% yield, 2 steps). **^{1}H NMR** (400 MHz, Chloroform-*d*) δ 11.66 (brs, 1H), 8.69 (d, *J* = 4 Hz, 1H), 8.24 (s, 1H), 8.00 (d, *J* = 9.2 Hz, 1H), 7.40 (d, *J* = 3.2 Hz, 1H), 7.30 (d, J = 9.2 Hz, 1H), 5.65-5.57 (m, 1H), 5.34 (brs, 1H), 4.90 (d, *J* = 17.2 Hz, 1H), 4.85 (d, *J* = 10.4 Hz, 1H), 3.66-3.61 (m, 1H), 3.31 (t, *J* = 6.4 Hz, 2H), 3.20 (t, *J* = 12 Hz, 1H), 2.97-2.88 (m, 2H), 2.58-2.54 (m, 1H), 2.36-2.35 (m, 1H), 2.09-2.04 (m, 1H), 1.97-1.89 (m, 1H), 1.82 (brs, 1H), 1.78-1.67 (m, 1H), 1.62-1.58 (m, 1H), 1.50-1.45 (m, 3H), 0.88-0.83

(m, 6H). **^{13}C NMR** (100 MHz, Chloroform-*d*) δ 156.99, 146.76, 144.85, 143.80, 140.97, 131.16, 128.28, 123.51, 118.10, 114.99, 108.08, 67.92, 59.62, 56.51, 43.40, 39.67, 39.12, 27.98, 27.15, 25.25, 22.79, 22.76

General Procedure for Synthesis of Fluorinated imines

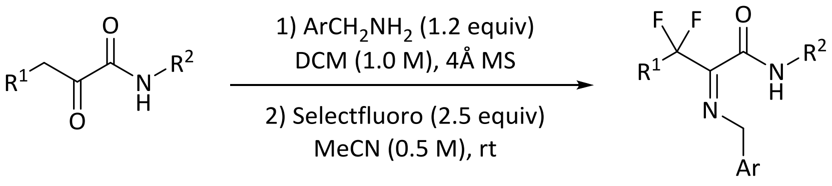

s-1 **1**

The α-ketoamides **s-1** were prepared according to literature procedures[1,2]. To a flame-dried Schlenk reaction tube equipped with a magnetic stir bar, was added the **s-1a** (843 mg, 3.0 mmol, 1.0 equiv), 2-$ClC_6H_4CH_2NH_2$ (508 mg, 3.6 mmol, 1.2 equiv) and 4Å MS (3.0 g). The Schlenk tube was closed with a septum, and DCM (3.0 mL) was added. The mixture was then stirred at 60 ℃ for about 12 h and monitored by GC-MS until **s-1a** was consumed. The mixture was concentrated under reduced pressure and the residue was dissolved in anhydrous MeCN (6.0 mL), selectfluoro (2.66g, 7.5 mmol, 2.5 equiv) was added to the mixture and stirred at r.t. for 0.5 h. Filtered and concentrated, purified by column chromatography (hexane/EtOAc = 30:1) to give compound **1a** (Z/E mixture) 331 mg, 25 % yield as a white solid. The compound could be stored for months under -25 ℃. **^{1}H NMR** (400 MHz, Chloroform-*d*) δ 7.43 – 7.26 (m, 9H), 7.25 – 7.16 (m, 5H), 6.20 (d, *J* = 6.9 Hz, 1H), 4.88 (t, *J* = 2.4 Hz, 2H), 4.61 (d, *J* = 5.8 Hz, 2H), 3.04 – 2.76 (m, 2H), 2.69 – 2.43 (m, 2H). **^{13}C NMR** (100 MHz, Chloroform-*d*) δ 161.5, 160.2 (t, *J* = 33.8 Hz), 140.3, 136.9, 135.6, 133.4, 129.4, 129.0, 128.6, 128.6, 128.5, 128.0, 127.8, 127.6, 127.1, 126.3, 120.3 (t, *J* = 241.7 Hz), 55.0, 43.4, 36.0 (t, *J* = 23.2 Hz), 28.1. **^{19}F NMR** (376 MHz, Chloroform-*d*) δ -98.68. **HRMS** (ESI) exact mass calcd. for $C_{25}H_{23}ClF_2N_2O$ ($[M + H]^+$) requires m/z 441.1540, found m/z 441.1557;

General Procedure for Catalytic Reaction of Fluorinated imines

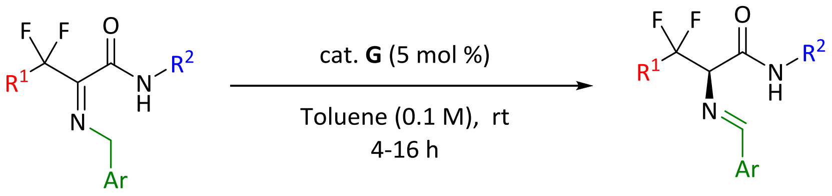

1 **2**

To a flame-dried Schlenk reaction tube equipped with a magnetic stir bar, was added the **1** (0.1 mmol) and cat. **G** (1.9 mg, 0.005 mmol). The Schlenk tube was closed with a septum, and Toluene (1.0 mL) was added. The mixture was then stirred at 25 ℃ and monitored by TLC until **1** was consumed. The mixture was concentrated under reduced pressure and purified by column chromatography on silica gel (hexane/EtOAc = 20:1 to 10:1) to afford the desired product **2**.

Sensitivity Assessment

To an oven-dried 10 mL Schlenk tube equipped with a stir bar was added starting material **1a** (44.1 mg, 0.1 mmol, 1.0 equiv.) and cat **G** (1.9 mg, 0.005 mmol, 5 mol%). The Schlenk tube was closed with a septum, and Toluene (1.0 mL) was added. The mixture was then stirred at 25 ℃ under Argon atmosphere and monitored by TLC until **1a** was consumed.[3]
Standard conditions: n = 0.1 mmol, c = 0.1 M, V = 1 mL, inert atmosphere, T = 25 °C,

Entry	Variations	Yield (%)	Deviation (yield)	ee (%)	Deviation (ee)
**1**	0.5 M	78	-9	93	-2
**2**	0.01 M	64	-26	96	1
**3**	10 uL H_2O	75	-13	94	-1
**4**	O_2	86	0	95	0
**5**	0 °C	78	-9	96	1
**6**	60 °C	81	-6	93	-2
**7**	1 mol% **G**	27	-69	96	1
**8**	10 mol% **G**	87	1	95	0
**9**	Standard condition	86	/	95	/

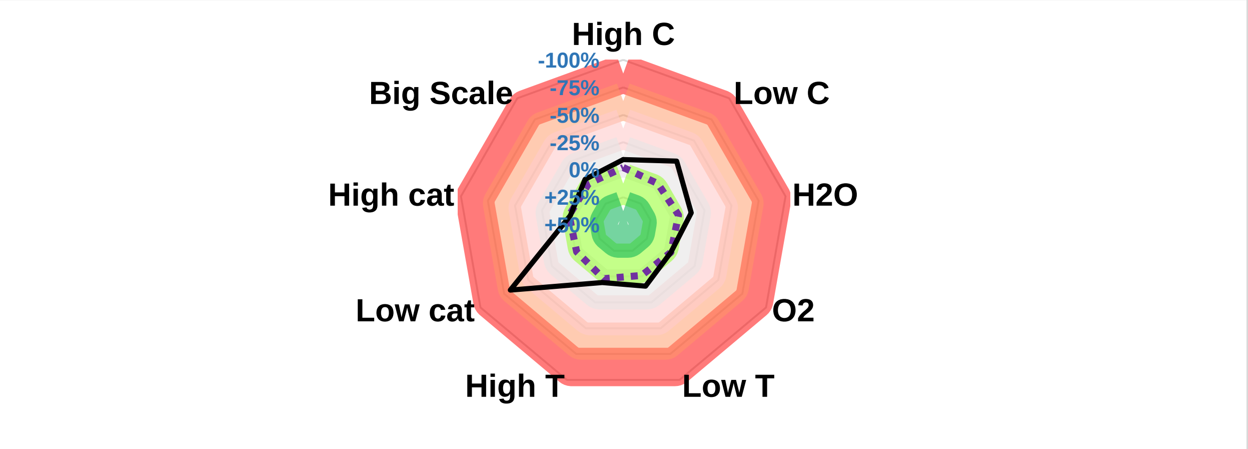

Supplementary Figure 119. Sensitivity Assessment

Solid line (—): deviation values of yield
Dashes line (··): deviation values of ee

Characterization Data

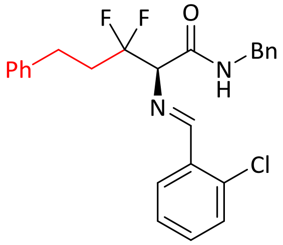

2a (*R*)-*N*-benzyl-2-((2-chlorobenzylidene)amino)-3,3-difluoro-5-phenylpentanamide
White solid, 37.8 mg, 86% yield, m.p. 79-81 ℃, 4 h; **^{1}H NMR** (400 MHz, Chloroform-*d*) δ 8.76 (s, 1H), 8.07 (d, *J* = 7.8 Hz, 1H), 7.45 (brs, 2H), 7.41 – 7.19 (m, 12H), 4.69 (dd, *J* = 15.3, 6.5 Hz, 1H), 4.10 – 5.57 (m, 2H), 3.14 – 2.79 (m, 2H), 2.65 – 2.23 (m, 2H). **^{13}C NMR** (100 MHz, Chloroform-*d*) δ 166.8, 163.7, 140.4, 137.8, 136.1, 132.9, 132.0, 130.2, 128.8, 128.7, 128.6, 128.5, 127.6, 127.5, 127.2, 126.3, 121.4 (t, *J* = 249.2 Hz), 77.5 (t, *J* = 23.9 Hz), 43.3, 36.3 (t, *J* = 23.5 Hz), 27.9. **^{19}F NMR** (376 MHz, Chloroform-*d*) δ -102.76 (d, *J* = 244.1 Hz), -109.04 (d, *J* = 243.9 Hz); **HRMS** (ESI) exact mass calcd. for $C_{25}H_{23}ClF_2N_2O$ ([M + H]$^{+}$) requires m/z 441.1540, found m/z 441.1540; **HPLC** (Chiralpak IC, *i*-propanol/hexane = 10/90, flow rate 1.0 mL/min, 40 ^{o}C, λ = 254 nm): t_R (major) = 15.3 min, t_R (minor) = 12.7 min, *ee* = 95%; $[\alpha]^{25}_{D}$ = -39.9 (*c* = 1.0, DCM).

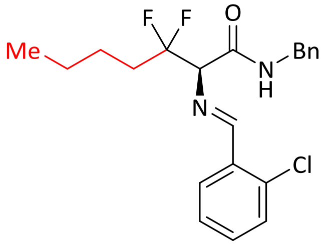

2b (*R*)-*N*-benzyl-2-((2-chlorobenzylidene)amino)-3,3-difluoroheptanamide
Pale yellow wax, 31.8 mg, 81% yield, 4 h; **^{1}H NMR** (400 MHz, Chloroform-*d*) δ 8.72 (s, 1H), 8.04 (d, *J* = 7.8 Hz, 1H), 7.41 (t, *J* = 3.0 Hz, 2H), 7.37 – 7.20 (m, 7H), 4.65 (dd, *J* = 15.3, 6.5 Hz, 1H), 4.43 (dd, *J* = 15.3, 5.5 Hz, 1H), 4.29 (dd, *J* = 16.0, 7.1 Hz, 1H), 2.14 – 1.93 (m, 2H), 1.65 – 1.45 (m, 2H), 1.44 – 1.31 (m, 2H), 0.92 (t, *J* = 7.2 Hz, 3H). **^{13}C NMR** (100 MHz, Chloroform-*d*) δ 166.9, 163.4, 137.9, 136.0, 132.8, 132.1, 130.1, 128.7, 128.7, 127.5, 127.5, 127.1, 121.9 (t, *J* = 249.9 Hz), 77.6 (t, *J* = 24.1 Hz), 43.3, 34.1 (t, *J* = 23.6 Hz), 23.7 (t, *J* = 4.2 Hz), 22.4, 13.9. **^{19}F NMR** (376 MHz, Chloroform-*d*) δ -102.81 (d, *J* = 244.1 Hz), -105.28 (d, *J* = 244.1 Hz); **HRMS** (ESI) exact mass calcd. for $C_{21}H_{24}ClF_2N_2O$ ([M + H]$^{+}$) requires m/z 393.1540, found m/z 393.1551; **HPLC** (Chiralpak IC, *i*-propanol/hexane = 10/90, flow rate 1.0 mL/min, 40 ^{o}C, λ = 254 nm): t_R (major) = 11.8 min, t_R (minor) = 10.0 min, *ee* = 96%; $[\alpha]^{25}_{D}$ = -25.9 (*c* = 1.0, DCM).

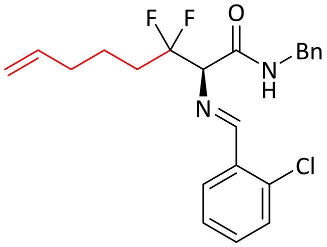

2c (*R*)-*N*-benzyl-2-((2-chlorobenzylidene)amino)-3,3-difluorooct-7-enamide

Pale yellow wax, 30.4 mg, 75% yield, 4 h; ^{1}**H NMR** (400 MHz, Chloroform-*d*) δ 8.72 (s, 1H), 8.03 (d, *J* = 7.7 Hz, 1H), 7.41 (d, *J* = 4.1 Hz, 2H), 7.32 (d, *J* = 6.8 Hz, 4H), 7.28 – 7.18 (m, 3H), 5.89 – 5.67 (m, 1H), 5.01 (dd, *J* = 19.4, 13.7 Hz, 2H), 4.65 (dd, *J* = 15.0, 6.5 Hz, 1H), 4.43 (dd, *J* = 15.1, 5.5 Hz, 1H), 4.29 (dd, *J* = 16.0, 7.9 Hz, 1H), 2.15 – 1.99 (m, 4H), 1.80 – 1.61 (m, 2H). ^{13}**C NMR** (100 MHz, Chloroform-*d*) δ 166.8, 163.4, 137.8, 137.8, 136.0, 132.8, 132.1, 130.1, 128.7, 128.7, 127.5, 127.5, 127.1, 121.8 (t, *J* = 249.0 Hz), 115.3, 77.5 (t, *J* = 29.7 Hz) 43.3, 33.7 (t, *J* = 23.5 Hz), 33.2, 20.8 (t, *J* = 4.2 Hz). ^{19}**F NMR** (376 MHz, Chloroform-*d*) δ-102.70 (d, *J* = 244.3 Hz), -105.32 (d, *J* = 244.3 Hz); **HRMS** (ESI) exact mass calcd. for $C_{22}H_{24}ClF_2N_2O$ ([M + H]$^{+}$) requires m/z 405.1540, found m/z 405.1532; **HPLC** (Chiralpak IC, *i*-propanol/hexane = 10/90, flow rate 1.0 mL/min, 40 °C, λ = 254 nm): t_R (major) = 11.7 min, t_R (minor) = 10.1 min, *ee* = 96%; $[\alpha]^{25}_{D}$ = -26.8 (*c* = 1.0, DCM).

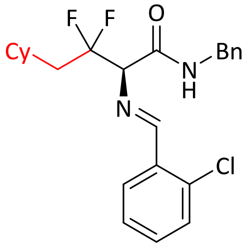

2d (*R*)-*N*-benzyl-2-((2-chlorobenzylidene)amino)-4-cyclohexyl-3,3-difluorobutanamide
Yellow wax, 37.6 mg, 87% yield, 4 h; ^{1}**H NMR** (400 MHz, Chloroform-*d*) δ 8.72 (s, 1H), 8.03 (d, *J* = 7.8 Hz, 1H), 7.44 – 7.36 (m, 2H), 7.37 – 7.24 (m, 6H), 7.20 (t, *J* = 6.3 Hz, 1H), 4.66 (dd, *J* = 14.9, 5.8 Hz, 1H), 4.42 (dd, *J* = 15.8, 5.4 Hz, 1H), 4.27 (dd, *J* = 15.4, 8.1 Hz, 1H), 2.04 – 1.63 (m, 7H), 1.31 – 0.85 (m, 6H). ^{13}**C NMR** (100 MHz, Chloroform-*d*) δ 166.9, 163.3, 137.9, 136.0, 132.8, 132.1, 130.1, 128.7, 128.7, 127.5, 127.5, 127.1, 122.2 (t, *J* = 249.6 Hz), 78.2 (t, *J* = 24.2 Hz), 43.3, 41.2 (t, *J* = 22.4 Hz), 34.0, 33.9, 31.9, 26.2. ^{19}**F NMR** (376 MHz, Chloroform-*d*) δ -100.19 (d, *J* = 244.4 Hz), -103.07 (d, *J* = 244.3 Hz); **HRMS** (ESI) exact mass calcd. for $C_{24}H_{28}ClF_2N_2O$ ([M + H]$^{+}$) requires m/z 433.1853, found m/z 433.1844; **HPLC** (Chiralpak IC, *i*-propanol/hexane = 10/90, flow rate 1.0 mL/min, 40 °C, λ = 254 nm): t_R (major) = 14.8 min, t_R (minor) = 11.8 min, *ee* = 96%; $[\alpha]^{25}_{D}$ = -20.7 (*c* = 1.0, DCM).

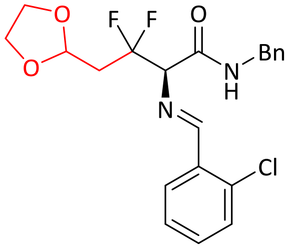

2e(*R*)-*N*-benzyl-2-((2-chlorobenzylidene)amino)-4-(1,3-dioxolan-2-yl)-3,3-difluorobutanamide
Yellow wax, 32.4 mg, 81% yield, 4 h; ^{1}**H NMR** (400 MHz, Chloroform-*d*) δ 8.78 (s, 1H), 8.04 (d, *J* = 7.8 Hz, 1H), 7.41 (d, *J* = 4.2 Hz, 2H), 7.38 – 7.26 (m, 5H), 7.18 (s, 1H), 5.26 (dt, *J* = 5.9, 3.0 Hz, 1H), 4.63 (dd, *J* = 15.1, 6.4 Hz, 1H), 4.53 – 4.40 (m, 2H), 4.13 – 3.95 (m, 2H), 3.95 – 3.84 (m, 2H), 2.71 – 2.36 (m, 2H). ^{13}**C NMR** (100 MHz, Chloroform-*d*) δ 166.6 (dd, *J* = 4.7, 1.9 Hz), 164.3, 137.8, 136.1, 132.9, 132.1, 130.1, 128.7, 128.7, 127.5, 127.4, 127.1, 120.3 (t, *J* = 249.8 Hz), 99.7, 76.8, 64.9, 64.7, 43.3, 39.1 (t, *J* = 22.5 Hz). ^{19}**F NMR** (376 MHz, Chloroform-*d*) δ -100.62 (d, *J* = 252.1 Hz), -102.40 (d, *J* = 252.1 Hz). **HRMS** (ESI) exact mass calcd. for $C_{21}H_{22}ClF_2N_2O_3$ ([M + H]$^{+}$) requires m/z 423.1282, found m/z 423.1274; **HPLC**

(Chiralpak IC, *i*-propanol/hexane = 20/80, flow rate 1.0 mL/min, 40 °C, λ = 254 nm): t_R (major) = 34.9 min, t_R (minor) = 20.5 min, *ee* = 94%; $[\alpha]^{25}_D$ = -39.0 (*c* = 1.0, DCM).

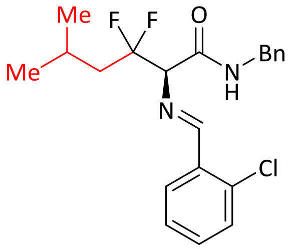

2f (*R*)-*N*-benzyl-2-((2-chlorobenzylidene)amino)-3,3-difluoro-5-methylhexanamide
Pale yellow wax, 25.5 mg, 65% yield, 4 h; **^{1}H NMR** (400 MHz, Chloroform-*d*) δ 8.72 (s, 1H), 8.03 (d, *J* = 7.8 Hz, 1H), 7.44 – 7.39 (m, 2H), 7.37 – 7.26 (m, 6H), 7.23 – 7.16 (m, 1H), 4.66 (dd, *J* = 15.1, 6.5 Hz, 1H), 4.43 (dd, *J* = 15.1, 5.6 Hz, 1H), 4.26 (dd, *J* = 15.7, 8.0 Hz, 1H), 2.27 – 1.82 (m, 3H), 1.00 (t, *J* = 7.1 Hz, 6H). **^{13}C NMR** (100 MHz, Chloroform-*d*) δ 166.9, 163.3, 137.9, 136.0, 132.8, 132.1, 130.1, 128.7, 128.7, 127.5, 127.5, 127.1, 122.0 (t, *J* = 250.4 Hz), 78.2 (t, *J* = 24.2 Hz), 43.3, 42.2 (t, *J* = 22.6 Hz), 23.7, 23.4, 22.8 (t, *J* = 2.9 Hz). **^{19}F NMR** (376 MHz, Chloroform-*d*) δ -101.06 (d, *J* = 244.1 Hz), -104.00 (d, *J* = 244.6 Hz). **HRMS** (ESI) exact mass calcd. for $C_{21}H_{24}ClF_2N_2O$ ($[M + H]^+$) requires m/z 393.1540, found m/z 393.1530; **HPLC** (Chiralpak IC, *i*-propanol/hexane = 10/90, flow rate 1.0 mL/min, 40 °C, λ = 254 nm): t_R (major) = 11.3 min, t_R (minor) = 9.3 min, *ee* = 94%; $[\alpha]^{25}_D$ = -40.2 (*c* = 1.0, DCM).

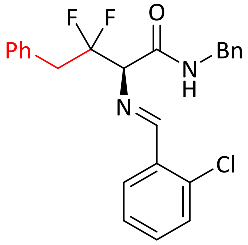

2g (*R*)-*N*-benzyl-2-((2-chlorobenzylidene)amino)-3,3-difluoro-4-phenylbutanamide
Pale yellow wax, 40.1 mg, 94% yield, 4 h; **^{1}H NMR** (400 MHz, Chloroform-*d*) δ 8.58 (s, 1H), 8.02 (d, *J* = 7.8 Hz, 1H), 7.48 – 7.11 (m, 14H), 4.67 (dd, *J* = 15.2, 6.5 Hz, 1H), 4.45 (dd, *J* = 15.1, 5.6 Hz, 1H), 4.28 (dd, *J* = 16.7, 7.6 Hz, 1H), 3.72 – 3.21 (m, 2H). **^{13}C NMR** (100 MHz, Chloroform-*d*) δ 166.9, 163.9, 137.8, 136.0, 132.8, 132.3, 132.0, 131.0, 130.1, 128.8, 128.7, 128.4, 127.5, 127.4, 127.1, 120.7 (t, *J* = 250.5 Hz), 76.3 (t, *J* = 23.7 Hz), 43.3, 40.7 (t, *J* = 24.0 Hz). **^{19}F NMR** (376 MHz, Chloroform-*d*) δ -101.27 (d, *J* = 246.1 Hz), -103.01 (d, *J* = 246.1 Hz). **HRMS** (ESI) exact mass calcd. for $C_{21}H_{24}ClF_2N_2O$ ($[M + H]^+$) requires m/z 427.1383, found m/z 427.1395; **HPLC** (Chiralpak IC, *i*-propanol/hexane = 10/90, flow rate 1.0 mL/min, 40 °C, λ = 254 nm): t_R (major) = 6.3 min, t_R (minor) = 5.8 min, *ee* = 94%; $[\alpha]^{25}_D$ = -18.5 (*c* = 1.0, DCM).

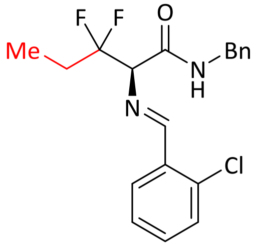

2h (*R*)-*N*-benzyl-2-((2-chlorobenzylidene)amino)-3,3-difluoropentanamide
Pale yellow wax, 32.8 mg, 90% yield, 4 h; ^{1}**H NMR** (400 MHz, Chloroform-*d*) δ 8.73 (s, 1H), 8.04 (d, *J* = 7.8 Hz, 1H), 7.45 – 7.38 (m, 2H), 7.38 – 7.25 (m, 5H), 7.28 – 7.19 (m, 1H), 4.65 (dd, *J* = 15.1, 6.4 Hz, 1H), 4.44 (dd, *J* = 15.1, 5.6 Hz, 1H), 4.30 (dd, *J* = 15.9, 8.1 Hz, 1H), 2.20 – 1.98 (m, 2H), 1.10 (t, *J* = 7.5 Hz, 3H). ^{13}**C NMR** (100 MHz, Chloroform-*d*) δ 166.9, 163.3, 137.8, 136.0, 132.8, 132.1, 130.1, 128.7, 128.7, 127.5, 127.4, 127.1, 120.8 (t, *J* = 249.2 Hz), 77.5, 43.3, 27.8 (t, *J* = 24.3 Hz), 6.0. ^{19}**F NMR** (376 MHz, Chloroform-*d*) δ -105.13 (d, *J* = 243.6 Hz), -107.34 (d, *J* = 243.6 Hz). **HRMS** (ESI) exact mass calcd. for $C_{19}H_{20}ClF_2N_2O$ ([M + H]$^+$) requires m/z 365.1227, found m/z 365.1239; **HPLC** (Chiralpak IC, *i*-propanol/hexane = 10/90, flow rate 1.0 mL/min, 40 °C, λ = 254 nm): t_R (major) = 21.7 min, t_R (minor) = 11.6 min, *ee* = 95%; $[\alpha]^{25}_D$ = -12.9 (*c* = 1.0, DCM).

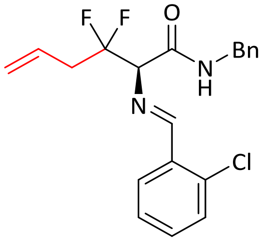

2i (*R*)-*N*-benzyl-2-((2-chlorobenzylidene)amino)-3,3-difluorohex-5-enamide
Pale yellow wax, 30.9 mg, 82% yield, 4 h; ^{1}**H NMR** (400 MHz, Chloroform-*d*) δ 8.70 (s, 1H), 8.03 (d, *J* = 7.7 Hz, 1H), 7.38 (d, *J* = 3.7 Hz, 2H), 7.33 – 7.22 (m, 7H), 6.03 – 5.71 (m, 1H), 5.36 – 5.16 (m, 2H), 4.62 (dd, *J* = 15.1, 6.4 Hz, 1H), 4.43 (dd, *J* = 15.1, 5.6 Hz, 1H), 4.32 (dd, *J* = 15.8, 8.5 Hz, 1H), 2.99 – 2.69 (m, 2H). ^{13}**C NMR** (100 MHz, Chloroform-*d*) δ 166.7 (dd, *J* = 4.8, 2.2 Hz), 163.7, 137.9, 136.0, 132.9, 132.1, 130.1, 128.7, 128.7, 128.6 (t, *J* = 5.3 Hz), 127.5, 127.4, 127.2, 121.2, 120.8 (t, *J* = 249.7 Hz), 76.8 (t, *J* = 23.8 Hz), 43.3, 39.1 (t, *J* = 24.1 Hz). ^{19}**F NMR** (376 MHz, Chloroform-*d*) δ -102.04 (d, *J* = 245.8 Hz), -104.04 (d, *J* = 245.7 Hz). **HRMS** (ESI) exact mass calcd. for $C_{20}H_{20}ClF_2N_2O$ ([M + H]$^+$) requires m/z 377.1227, found m/z 377.1236; **HPLC** (Chiralpak IC, *i*-propanol/hexane = 10/90, flow rate 1.0 mL/min, 40 °C, λ = 254 nm): t_R (major) = 12.1 min, t_R (minor) = 10.0 min, *ee* = 94%; $[\alpha]^{25}_D$ = -20.6 (*c* = 1.0, DCM).

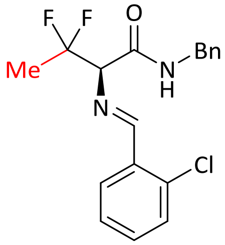

2j (*R*)-*N*-benzyl-2-((2-chlorobenzylidene)amino)-3,3-difluorobutanamide
White solid, 30.9 mg, 88% yield, 4 h; ^{1}**H NMR** (400 MHz, Chloroform-*d*) δ 8.73 (s, 1H), 8.04 (d, *J* = 7.8 Hz, 1H), 7.44 – 7.37 (m, 2H), 7.36 – 7.20 (m, 7H), 4.65 (dd, *J* = 15.3, 6.4 Hz, 1H), 4.43 (dd, *J* = 15.0, 5.5 Hz, 1H), 4.26 (dd, *J* = 15.9, 7.3 Hz, 1H), 1.79 (t, *J* = 19.0 Hz, 3H). ^{13}**C NMR** (100 MHz, Chloroform-*d*) δ 166.8, 163.6, 137.8, 136.1, 132.8, 132.0, 130.1, 128.8, 128.7, 127.5, 127.5, 127.1, 121.1 (t, *J* = 246.3 Hz), 78.3 (t, *J* = 24.0 Hz), 43.3, 21.6 (t, *J* = 25.8 Hz). ^{19}**F NMR** (376 MHz, Chloroform-*d*) δ -93.80 (d, *J* = 243.9 Hz), -97.80 (d, *J* = 243.8 Hz). **HRMS** (ESI) exact mass calcd. for $C_{18}H_{18}ClF_2N_2O$ ([M + H]$^+$) requires m/z 351.1570, found m/z 351.1557;

HPLC (Chiralpak IC, *i*-propanol/hexane = 10/90, flow rate 1.0 mL/min, 40 °C, λ = 254 nm): t_R (major) = 14.8 min, t_R (minor) = 11.8 min, *ee* = 96%; $[\alpha]^{25}_D$ = -51.0 (*c* = 1.0, DCM).

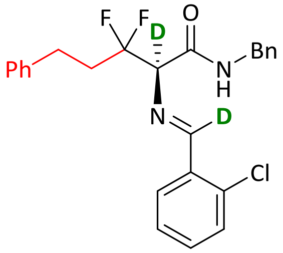

2k (*R*)-*N*-benzyl-2-(((2-chlorophenyl)methylene-d)amino)-3,3-difluoro-5-phenylpentanamide
Pale yellow wax, 27.0 mg, 61% yield, 16 h; **^{1}H NMR** (400 MHz, Chloroform-*d*) δ 8.03 (d, J = 7.7 Hz, 1H), 7.44 – 7.25 (m, 9H), 7.23 – 7.12 (m, 4H), 4.71 – 4.53 (m, 1H), 4.43 (dd, J = 15.1, 5.6 Hz, 1H), 3.03 – 2.74 (m, 2H), 2.49 – 2.21 (m, 2H). **^{13}C NMR** (100 MHz, Chloroform-*d*) δ 166.7, 163.3, 140.4, 137.8, 136.1, 132.9, 132.0, 130.1, 128.8, 128.7, 128.5, 128.4, 127.5, 127.5, 127.1, 126.2, 121.4 (t, J = 249.5 Hz), 77.2, 43.3, 36.3 (t, J = 23.5 Hz), 27.9 (t, J = 4.8 Hz). **^{19}F NMR** (376 MHz, Chloroform-*d*) δ -103.08 (d, J = 244.8 Hz), -105.45 (d, J = 244.5 Hz). **HRMS** (ESI) exact mass calcd. for $C_{25}H_{22}D_2ClF_2N_2O$ ([M + H]$^+$) requires m/z 443.1665, found m/z 443.1659; **HPLC** (Chiralpak IC, *i*-propanol/hexane = 10/90, flow rate 1.0 mL/min, 40 °C, λ = 254 nm): t_R (major) = 15.7 min, t_R (minor) = 12.9 min, *ee* = 92%; $[\alpha]^{25}_D$ = -35.6 (*c* = 1.0, DCM).

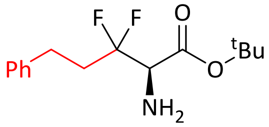

2l *tert*-butyl (*R*)-2-amino-3,3-difluoro-5-phenylpentanoate
Pale yellow wax, 17.7 mg, 62% yield, 8 h; **^{1}H NMR** (400 MHz, Chloroform-*d*) 7.33 – 7.26 (m, 2H), 7.23 – 7.16 (m, 3H), 3.68 (t, J = 12.0 Hz, 1H), 2.95 – 2.68 (m, 2H), 2.53 – 2.11 (m, 2H), 1.98 (s, 2H), 1.49 (s, 9H). **^{13}C NMR** (100 MHz, Chloroform-*d*) δ 168.9 (t, J = 3.8 Hz), 140.5, 128.6, 128.3, 126.2, 122.5 (t, J = 247.2 Hz), 82.6, 59.7 (t, J = 27.1 Hz), 35.6 (t, J = 24.2 Hz), 29.7, 27.9. **^{19}F NMR** (376 MHz, Chloroform-*d*) δ -106.93 (d, J = 246.0 Hz), -107.69 (d, J = 245.3 Hz). **HRMS** (ESI) exact mass calcd. for $C_{15}H_{22}F_2NO_2$ ([M + H]$^+$) requires m/z 286.1613, found m/z 286.1609; **HPLC** (Chiralpak IC, *i*-propanol/hexane = 5/95, flow rate 1.0 mL/min, 40 °C, λ = 190 nm): t_R (major) = 13.0 min, t_R (minor) = 8.2 min, *ee* = 72%; $[\alpha]^{25}_D$ = -30.6 (*c* = 1.0, DCM).

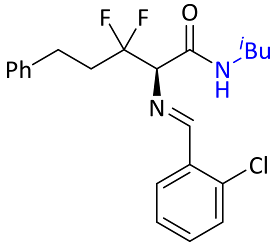

2m (*R*)-2-((2-chlorobenzylidene)amino)-3,3-difluoro-*N*-isobutyl-5-phenylpentanamid
Yellow wax, 34.6 mg, 85% yield, 4 h; **^{1}H NMR** (400 MHz, Chloroform-*d*) δ 8.72 (s, 1H), 8.08 (d, *J* = 7.7 Hz, 1H), 7.43 (d, *J* = 3.6 Hz, 2H), 7.39 – 7.33 (m, 1H), 7.31 – 7.26 (m, 2H), 7.24 –

7.18 (m, 3H), 6.94 (t, *J* = 6.2 Hz, 1H), 4.27 (dd, *J* = 16.3, 7.9 Hz, 1H), 3.24 (dt, *J* = 13.4, 6.7 Hz, 1H), 3.09 (dt, *J* = 13.1, 6.3 Hz, 1H), 3.02 – 2.91 (m, 1H), 2.91 – 2.79 (m, 1H), 2.51 – 2.27 (m, 2H), 1.82 (dt, *J* = 13.4, 6.7 Hz, 1H), 0.91 (d, *J* = 6.7 Hz, 6H). **^{13}C NMR** (100 MHz, Chloroform-*d*) δ 166.7, 163.3, 140.5, 136.1, 132.8, 132.1, 130.2, 128.6, 128.5, 128.5, 127.2, 126.2, 121.4 (t, *J* = 249.4 Hz), 77.6 (t, *J* = 25.5 Hz), 46.6, 36.4 (t, *J* = 23.5 Hz), 28.5, 28.0, 20.0, 20.0. **^{19}F NMR** (376 MHz, Chloroform-*d*) δ -103.46 (d, *J* = 244.5 Hz), -105.92 (d, *J* = 244.5 Hz). **HRMS** (ESI) exact mass calcd. for $C_{22}H_{26}ClF_2N_2O$ ([M + H]$^+$) requires m/z 407.1696, found m/z 407.1688; **HPLC** (Chiralpak IC, *i*-propanol/hexane = 10/90, flow rate 1.0 mL/min, 40 °C, λ = 254 nm): t_R (major) = 9.9 min, t_R (minor) = 7.8 min, *ee* = 97%; $[\alpha]^{25}_D$ = -25.8 (*c* = 1.0, DCM).

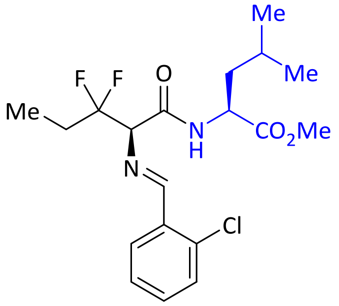

2n methyl ((*R*)-2-(((*E*)-2-chlorobenzylidene)amino)-3,3-difluoropentanoyl)-L-leucinate
Clorless wax, 28.2 mg, 70% yield, dr = 56/1, 4 h; **^{1}H NMR** (400 MHz, Chloroform-*d*) δ 8.73 (s, 1H), 8.09 (d, *J* = 7.8 Hz, 1H), 7.43 (d, *J* = 4.1 Hz, 2H), 7.40 – 7.32 (m, 1H), 7.22 (d, *J* = 8.2 Hz, 1H), 4.66 (q, *J* = 7.6 Hz, 1H), 4.27 (dd, *J* = 14.7, 8.3 Hz, 1H), 3.77 (s, 3H), 2.20 – 1.99 (m, 2H), 1.74 – 1.56 (m, 3H), 1.14 – 1.04 (m, 3H), 0.90 (d, *J* = 6.0 Hz, 6H). **^{13}C NMR** (100 MHz, Chloroform-*d*) δ 172.8, 166.7 (t, *J* = 3.9 Hz), 163.3, 136.0, 132.8, 132.1, 130.1, 128.6, 127.2, 121.9 (t, *J* = 249.2 Hz), 77.2, 52.4, 50.8, 41.2, 27.2 (t, *J* = 24.3 Hz), 25.0, 22.7, 22.0, 5.8. **^{19}F NMR** (376 MHz, Chloroform-*d*) -104.75 (d, *J* = 243.7 Hz), -106.70 (d, *J* = 243.8 Hz). **HRMS** (ESI) exact mass calcd. for $C_{19}H_{26}ClF_2N_2O_3$ ([M + H]$^+$) requires m/z 403.1595, found m/z 403.1578.

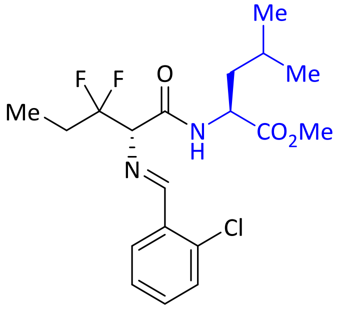

2o methyl ((*S*)-2-(((*E*)-2-chlorobenzylidene)amino)-3,3-difluoropentanoyl)-L-leucinate
Clorless wax, 30.2 mg, 75% yield, dr = 41/1, 4 h; **^{1}H NMR** (400 MHz, Chloroform-*d*) δ 8.72 (s, 1H), 8.14 (d, *J* = 7.7 Hz, 1H), 7.45 – 7.40 (m, 2H), 7.41 – 7.31 (m, 2H), 4.71 (q, *J* = 6.7, 4.5 Hz, 1H), 4.24 (dd, *J* = 15.7, 8.2 Hz, 1H), 3.70 (s, 3H), 2.17 – 1.96 (m, 2H), 1.69 (ddt, *J* = 19.8, 10.5, 4.2 Hz, 3H), 1.10 (t, *J* = 7.5 Hz, 3H), 1.02 – 0.94 (m, 6H). **^{13}C NMR** (100 MHz, Chloroform-*d*) δ 173.0, 166.6, 163.5, 136.0, 132.8, 132.2, 130.1, 128.7, 127.2, 121.9 (t, *J* = 249.1 Hz), 77.0, 52.3, 50.6, 41.6, 27.7 (t, *J* = 24.4 Hz), 24.8, 22.9, 21.9, 6.0 (t, *J* = 5.5 Hz). **^{19}F NMR** (376 MHz, Chloroform-*d*) -105.34 (d, *J* = 243.8 Hz), -107.43 (d, *J* = 243.8 Hz). **HRMS** (ESI) exact

mass calcd. for $C_{19}H_{26}ClF_2N_2O_3$ ($[M + H]^+$) requires m/z 403.1595, found m/z 403.1584.

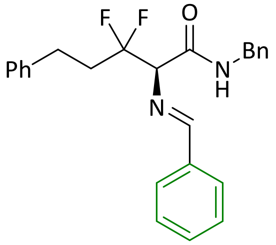

2p (*R*)-*N*-benzyl-2-(benzylideneamino)-3,3-difluoro-5-phenylpentanamide
Clorless wax, 32.1 mg, 79% yield, 8 h; **^{1}H NMR** (400 MHz, Chloroform-*d*) δ 8.28 (s, 1H), 7.78 (d, *J* = 7.3 Hz, 2H), 7.54 – 7.40 (m, 3H), 7.28 (dd, *J* = 11.5, 6.7 Hz, 7H), 7.19 (d, *J* = 7.7 Hz, 3H), 4.65 (dd, *J* = 16.2, 7.0 Hz, 1H), 4.43 (dd, *J* = 14.9, 5.0 Hz, 1H), 4.27 (dd, *J* = 14.7, 8.0 Hz, 1H), 3.01 – 2.89 (m, 1H), 2.90 – 2.78 (m, 1H), 2.47 – 2.23 (m, 2H). **^{13}C NMR** (100 MHz, Chloroform-*d*) δ 167.0, 166.7, 140.5, 137.9, 134.9, 132.1, 128.9, 128.8, 128.7, 128.5, 128.5, 127.5, 127.5, 126.2, 121.5 (t, *J* = 249.8 Hz), 77.6, 43.2, 36.2 (t, *J* = 23.6 Hz), 27.8. **^{19}F NMR** (376 MHz, Chloroform-*d*) δ -103.12 (d, *J* = 244.6 Hz), -105.36 (d, *J* = 244.6 Hz). **HRMS** (ESI) exact mass calcd. for $C_{25}H_{25}F_2N_2O$ ($[M + H]^+$) requires m/z 407.1929, found m/z 407.1943; **HPLC** (Chiralpak IC, *i*-propanol/hexane = 10/90, flow rate 1.0 mL/min, 40 ^oC, λ = 254 nm): t_R (major) = 18.7 min, t_R (minor) = 13.7 min, *ee* = 99%; $[\alpha]^{25}_D$ = -32.6 (*c* = 1.0, DCM).

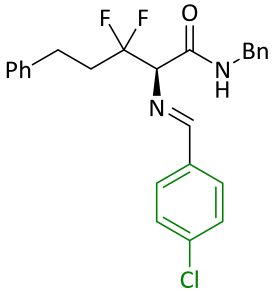

2q (*R*)-*N*-benzyl-2-((4-chlorobenzylidene)amino)-3,3-difluoro-5-phenylpentanamide
Yellow wax, 34.8 mg, 79% yield, 4 h; **^{1}H NMR** (400 MHz, Chloroform-*d*) δ 8.23 (s, 1H), 7.72 (d, *J* = 8.5 Hz, 2H), 7.41 (d, *J* = 8.4 Hz, 2H), 7.34 – 7.26 (m, 6H), 7.24 – 7.16 (m, 4H), 4.64 (dd, *J* = 15.1, 6.5 Hz, 1H), 4.43 (dd, *J* = 15.1, 5.6 Hz, 1H), 4.26 (dd, *J* = 15.4, 8.3 Hz, 1H), 3.01 – 2.89 (m, 1H), 2.88 – 2.77 (m, 1H), 2.47 – 2.21 (m, 2H). **^{13}C NMR** (100 MHz, Chloroform-*d*) δ 166.8, 165.4, 140.4, 138.2, 137.8, 133.3, 130.0, 129.2, 128.8, 128.6, 128.5, 127.6, 127.5, 126.3, 121.4 (t, *J* = 249.6 Hz), 77.6, 43.3, 36.3 (t, *J* = 23.5 Hz), 27.8 (t, *J* = 4.6 Hz). **^{19}F NMR** (376 MHz, Chloroform-*d*) δ -103.05 (d, *J* = 244.7 Hz), -105.41 (d, *J* = 244.9 Hz). **HRMS** (ESI) exact mass calcd. for $C_{25}H_{24}ClF_2N_2O$ ($[M + H]^+$) requires m/z 441.1540, found m/z 441.1556; **HPLC** (Chiralpak IC, *i*-propanol/hexane = 10/90, flow rate 1.0 mL/min, 40 ^oC, λ = 254 nm): t_R (major) = 17.3 min, t_R (minor) = 12.3 min, *ee* = 98%; $[\alpha]^{25}_D$ = -62.9 (*c* = 1.0, DCM).

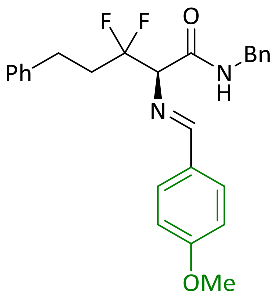

2r (*R*)-*N*-benzyl-3,3-difluoro-2-((4-methoxybenzylidene)amino)-5-phenylpentanamide
Yellow wax, 30.1 mg, 69% yield, 16 h; ^{1}**H NMR** (400 MHz, Chloroform-*d*) δ 8.18 (s, 1H), 7.72 (d, *J* = 8.3 Hz, 2H), 7.38 – 7.27 (m, 7H), 7.22 – 7.16 (m, 3H), 6.94 (d, *J* = 8.3 Hz, 2H), 4.64 (dd, *J* = 15.1, 6.5 Hz, 1H), 4.42 (dd, *J* = 15.1, 5.9 Hz, 1H), 4.22 (dd, *J* = 14.9, 8.8 Hz, 1H), 3.85 (s, 3H), 3.04 – 2.90 (m, 1H), 2.88 – 2.78 (m, 1H), 2.46 – 2.25 (m, 1H). ^{13}**C NMR** (100 MHz, Chloroform-*d*) δ 167.3, 165.9, 162.7, 140.6, 137.9, 130.6, 129.0, 128.7, 128.5, 128.5, 127.9, 127.9, 127.5, 126.2, 124.0 (t, *J* = 249.1 Hz), 114.2, 55.5, 43.2, 36.2 (t, *J* = 23.7 Hz), 27.8. ^{19}**F NMR** (376 MHz, Chloroform-*d*) δ -103.18 (d, *J* = 243.9 Hz), -105.36 (d, *J* = 243.9 Hz). **HRMS** (ESI) exact mass calcd. for $C_{26}H_{27}F_{2}N_{2}O_{2}$ ([M + H]$^{+}$) requires m/z 437.2035, found m/z 437.2027; **HPLC** (Chiralpak IC, *i*-propanol/hexane = 10/90, flow rate 1.0 mL/min, 40 ^{o}C, λ = 254 nm): t_R (major) = 14.4 min, t_R (minor) = 11.0 min, *ee* = 97%; $[\alpha]^{25}_{D}$ = -28.6 (*c* = 1.0, DCM).

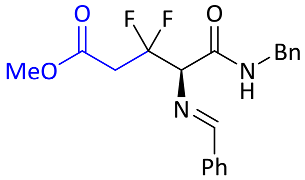

2s Methyl (*R*)-5-(benzylamino)-4-(benzylideneamino)-3,3-difluoro-5-oxopentanoate
Yellow wax, 28.1 mg, 75% yield, 4 h; ^{1}**H NMR** (400 MHz, Chloroform-*d*) δ 8.38 (s, 1H), 7.96 – 7.71 (m, 2H), 7.65 – 7.38 (m, 4H), 7.36 – 7.27 (m, 4H), 4.74 (dd, *J* = 19.9, 6.9 Hz, 1H), 4.63 (dd, *J* = 15.1, 6.4 Hz, 1H), 4.45 (dd, *J* = 15.2, 5.6 Hz, 1H), 3.75 (s, 3H), 3.61 – 3.40 (m, 1H), 3.28 – 3.03 (m, 1H). ^{13}**C NMR** (100 MHz, Chloroform-*d*) δ 168.1, 167.6, 167.0, 137.7, 134.9, 132.1, 128.9, 128.8, 128.8, 127.5, 127.4, 120.5 (t, *J* = 248.1 Hz), 75.0 (t, *J* = 22.5 Hz), 52.2, 43.2, 39.8 (t, *J* = 25.5 Hz). ^{19}**F NMR** (376 MHz, Chloroform-*d*) δ -99.15 (d, *J* = 253.6 Hz), -102.01 (d, *J* = 253.5 Hz). **HRMS** (ESI) exact mass calcd. for $C_{20}H_{21}F_{2}N_{2}O_{3}$ ([M + H]$^{+}$) requires m/z 375.1515, found m/z 375.1505; **HPLC** (Chiralpak IC, *i*-propanol/hexane = 10/90, flow rate 1.0 mL/min, 40 ^{o}C, λ = 254 nm): t_R (major) = 17.7 min, t_R (minor) = 16.2 min, *ee* = 96%; $[\alpha]^{25}_{D}$ = -54.7 (*c* = 1.0, DCM).

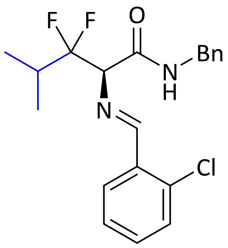

2t (*R*)-*N*-benzyl-2-((2-chlorobenzylidene)amino)-3,3-difluoro-4-methylpentanamide
Colorless wax, 35.6 mg, 94% yield, 4 h; ^{1}**H NMR** (400 MHz, Chloroform-*d*) δ 8.73 (s, 1H), 8.05

(d, J = 7.8 Hz, 1H), 7.41 (d, J = 4.0 Hz, 2H), 7.36 – 7.25 (m, 6H), 7.24 – 7.15 (m, 1H), 4.64 (dd, J = 15.2, 6.4 Hz, 1H), 4.48 – 4.37 (m, 2H), 2.54 – 2.27 (m, 1H), 1.18 (d, J = 6.9 Hz, 3H), 1.06 (d, J = 6.9 Hz, 3H). **^{13}C NMR** (100 MHz, Chloroform-*d*) δ 167.0, 163.1, 137.9, 136.0, 132.7, 132.2, 130.1, 128.7, 128.7, 127.5, 127.4, 127.1, 123.1 (t, J = 251.6 Hz), 75.8 (t, J = 24.1 Hz), 43.3, 32.4 (t, J = 23.1 Hz), 16.3 (dd, J = 6.1, 3.4 Hz), 14.8 (t, J = 5.2 Hz). **^{19}F NMR** (376 MHz, Chloroform-*d*) δ -112.59 (d, J = 246.0 Hz), -114.72 (d, J = 245.5 Hz). **HRMS** (ESI) exact mass calcd. for $C_{20}H_{22}ClF_2N_2O$ ([M + H]$^+$) requires m/z 379.1383, found m/z 379.1399; **HPLC** (Chiralpak IC, *i*-propanol/hexane = 10/90, flow rate 1.0 mL/min, 40 °C, λ = 254 nm): t_R (major) = 9.7 min, t_R (minor) = 8.9 min, *ee* = 95%; $[\alpha]^{25}_D$ = -34.4 (*c* = 1.0, DCM).

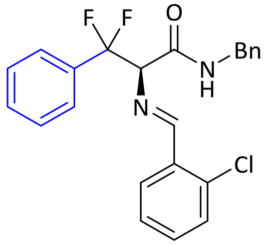

2u (*R*)-*N*-benzyl-2-((2-chlorobenzylidene)amino)-3,3-difluoro-3-phenylpropanamide
Colorless wax, 25.6 mg, 62% yield, 4 h; **^{1}H NMR** (400 MHz, Chloroform-*d*) δ 8.65 (s, 1H), 8.11 – 7.85 (m, 1H), 7.49 – 7.35 (m, 8H), 7.34 – 7.26 (m, 4H), 7.15 – 7.06 (m, 2H), 6.91 (s, 1H), 4.78 – 4.45 (m, 2H), 4.28 (dd, J = 15.1, 5.3 Hz, 1H). **^{13}C NMR** (100 MHz, Chloroform-*d*) δ 166.1 (d, J = 4.2 Hz), 163.7, 137.7, 136.1, 134.0 (t, J = 25.8 Hz), 132.9, 132.0, 130.3, 130.2, 128.6, 128.5, 128.2, 127.6, 127.4, 127.2, 126.1 (t, J = 6.4 Hz), 119.5 (t, J = 250.6 Hz), 79.4 (t, J = 27.6 Hz), 43.2. **^{19}F NMR** (376 MHz, Chloroform-*d*) δ -98.81 (d, J = 247.0 Hz), -100.71 (d, J = 246.6 Hz). **HRMS** (ESI) exact mass calcd. for $C_{20}H_{22}ClF_2N_2O$ ([M + H]$^+$) requires m/z 379.1383, found m/z 379.1396; **HPLC** (Chiralpak IC, *i*-propanol/hexane = 10/90, flow rate 1.0 mL/min, 40 °C, λ = 254 nm): t_R (major) = 24.7 min, t_R (minor) = 20.5 min, *ee* = 92%; $[\alpha]^{25}_D$ = -39.7 (*c* = 1.0, DCM).

Gram Scale Synthesis Procedure

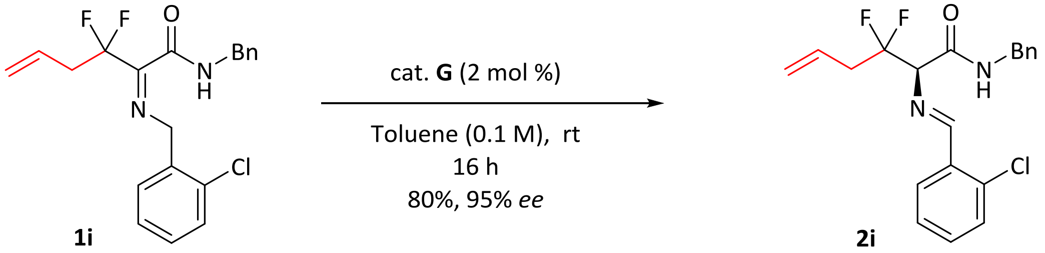

To a flame-dried Schlenk reaction tube equipped with a magnetic stir bar, was added the **1i** (1.13g, 3.0 mmol) and cat. **G** (22.9 mg, 0.06 mmol). The Schlenk tube was closed with a septum, and Toluene (30 mL) was added. The mixture was then stirred at 25 ℃ and monitored by TLC until **1i** was consumed. The mixture was concentrated under reduced pressure and purified by column chromatography on silica gel (hexane/EtOAc = 30:1) to afford the desired product **2i** as a pale yellow wax, 0.91 g, 80% yield, 95% ee.

Synthetic Transformation

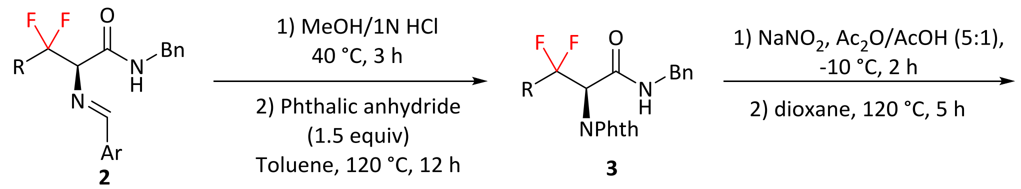

2 **3** **4**

To a solution of compound **2** (0.2 mmol) in MeOH (2.0 mL) was added 1N HCl (1.0 mL), the mixture was stirred at 40 ℃ for 3 h[4]. Then the pH of the reaction mixture was adjusted to about 9 with $NaHCO_3$, extracted with EA, and concentrated to give intermediate, which was used further purification through a short silica column. To a solution of intermediate in toluene (2.0 mL) was added phthalic anhydride (0.24 mmol, 1.2 euqiv), the mixture was stirred at 120 ℃ for 12 h. The reaction mixture was poured into water and extracted with EA, purified by column chromatography to give compound **3**. To a solution of **3** (0.1 mmol) in a 5:1 mixture of Ac_2O and AcOH (1.0 mL) was added granular $NaNO_2$ (2.0 mmol) in portions over 20 min at -10 ℃. After 2 h, the mixture was warmed to room temperature within 20 min, added into ice-water (25.0 mL), and extracted with Et_2O or CH_2Cl_2 (three times). The combined organic layer was washed (carefully!) with 5% Na_2CO_3 (three times) and then H_2O and dried (Na_2SO_4). Evaporation of solvents gave a yellowish liquid. To this residue was added anhydrous 1,4-dioxane (1.0 mL), and the solution was refluxed for 5 h. The mixture was concentrated under reduced pressure and purified by column chromatography on silica gel (hexane/EtOAc = 30:1) to afford the desired product **4**.

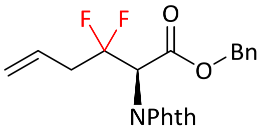

4a

4a Benzyl (*R*)-2-(1,3-dioxoisoindolin-2-yl)-3,3-difluorohex-5-enoate
Colorless wax, 23.9 mg, 62% yield; **^{1}H NMR** (400 MHz, Chloroform-*d*) δ 7.89 (d, J = 7.5, 3.7 Hz, 2H), 7.77 (dd, J = 5.5, 3.1 Hz, 2H), 7.35 – 7.27 (m, 5H), 5.96 – 5.64 (m, 1H), 5.38 – 5.07 (m, 5H), 3.31 – 3.07 (m, 1H), 3.01 – 2.80 (m, 1H).**^{13}C NMR** (100 MHz, Chloroform-*d*) δ 166.6, 164.1 (t, J = 4.4 Hz), 134.6, 134.5, 131.6, 128.6, 128.6, 128.3, 128.2 (dd, J = 7.4, 3.8 Hz), 123.9, 121.3, 120.5 (dd, J = 251.5, 247.5 Hz), 68.1, 54.4 (dd, J = 30.2, 28.1 Hz), 39.4 (t, J = 23.8 Hz). **^{19}F NMR** (376 MHz, Chloroform-*d*) δ -97.59 (d, J = 253.6 Hz), -100.10 (d, J = 253.7 Hz). **HRMS** (ESI) exact mass calcd. for $C_{21}H_{18}F_2NO_4$ ($[M + H]^+$) requires m/z 386.1198, found m/z 386.1191. **HPLC** (Chiralpak IC, *i*-propanol/hexane = 10/90, flow rate 1.0 mL/min, 40 °C, λ = 254 nm): t_R (major) = 7.3 min, t_R (minor) = 9.7 min, *ee* = 84%; $[\alpha]^{25}_D$ = -48.5 (*c* = 1.0, DCM).

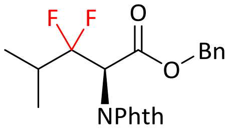

4b

4b Benzyl (*R*)-2-(1,3-dioxoisoindolin-2-yl)-3,3-difluoro-4-methylpentanoate
Pale yellow wax, 26.3 mg, 68% yield; **^{1}H NMR** (400 MHz, Chloroform-*d*) δ 7.90 (dd, J = 5.4, 3.1 Hz, 2H), 7.77 (dd, J = 5.5, 3.0 Hz, 2H), 7.34 – 7.26 (m, 5H), 5.35 (dd, J = 20.5, 7.9 Hz, 1H), 5.30 – 5.17 (m, 2H), 2.91 – 2.59 (m, 1H), 1.12 (d, J = 6.9 Hz, 3H), 1.08 (d, J = 6.8 Hz, 3H). **^{13}C**

NMR (100 MHz, Chloroform-*d*) δ 166.8, 164.4 (d, J = 7.9 Hz), 134.7, 134.5, 131.6, 128.6, 128.5, 128.3, 123.9, 123.1 (t, J = 254.6 Hz), 68.1, 53.5 (dd, J = 29.9, 27.6 Hz), 32.9 (t, J = 22.7 Hz), 16.1 (dd, J = 6.3, 3.4 Hz), 14.8 (t, J = 5.3 Hz). **^{19}F NMR** (376 MHz, Chloroform-*d*) -108.07 (d, J = 250.6 Hz), -111.20 (d, J = 250.6 Hz). **HRMS** (ESI) exact mass calcd. for $C_{21}H_{20}F_2NO_4$ ($[M + H]^+$) requires m/z 388.1355, found m/z 388.1367. **HPLC** (Chiralpak IC, *i*-propanol/hexane = 10/90, flow rate 1.0 mL/min, 40 °C, λ = 254 nm): t_R (major) = 21.5 min, t_R (minor) = 24.8 min, *ee* = 90%; $[\alpha]^{25}_D$ = -36.4 (*c* = 1.0, DCM).

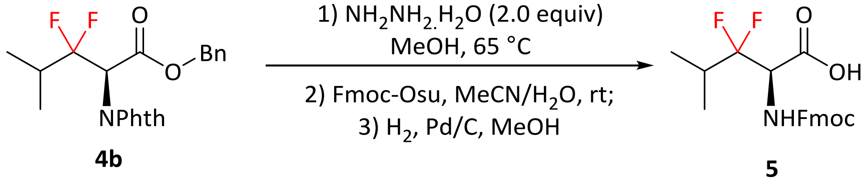

To an oven-dried pressure tube, **4b** (0.3 mmol, 1.0 equiv) and hydrazine hydrate (30.0 mg, 0.6 mmol, 2.0 equiv) were dissolved in 2.0 mL of MeOH[5]. The tube was then sealed and stirred at 65 ℃ for 10 h. After the reaction, the solvent was removed. The crude product was dissolved in 20.0 mL DCM, and undissolved solid was filtered. The filtrate was concentrated in vacuo and further purification through a short silica column. Then the residue was dissolved in MeCN/H_2O (5/1, 3.0 mL), Fmoc-OSu (0.45 mmol, 1.5 equiv) was added to the mixture and stirred at r.t. for 48 h. After that time, the reaction mixture was dilute with water and extracted with EA, purified by column chromatography to give intermediate. To a solution of the intermediate in THF/MeOH (3.0 mL, 4/1) was added Pd/C (5%, 10.0 mg), the reaction was stirred at r.t. under H_2 atmosphere for 2 h, filtered and concentrated to give compound **5** (*R*)-*N*-Fmoc-*β*,*β*-difluoro-Leu, 50.2 mg, 43% yield in three steps, white solid, 90% *ee*, (recrystallization from hexane/ ethyl acetate (5/1), 99% ee) **^{1}H NMR** (400 MHz, Acetone-*d*$_6$) δ 7.85 (d, *J* = 7.6 Hz, 2H), 7.74 (dd, *J* = 7.8, 4.0 Hz, 2H), 7.41 (t, *J* = 7.5 Hz, 2H), 7.35 – 7.29 (m, 2H), 7.22 (d, *J* = 9.9 Hz, 1H), 5.04 – 4.79 (m, 1H), 4.51 – 4.31 (m, 2H), 4.25 (t, *J* = 7.3 Hz, 1H), 2.53 – 2.31 (m, 1H), 1.08 (dd, *J* = 12.5, 6.9 Hz, 6H). **^{13}C NMR** (100 MHz, Acetone-*d*$_6$) δ 168.7, 157.0, 144.8, 144.7, 142.0, 128.5, 127.8, 126.1, 120.7 (d, *J* = 2.0 Hz), 67.7, 56.9 (t, *J* = 27.1 Hz), 47.8, 32.8 (t, *J* = 23.1 Hz), 15.7 (t, *J* = 5.1 Hz), 15.3 (t, *J* = 5.0 Hz). **^{19}F NMR** (376 MHz, acetone-*d*$_6$) δ –113.4 - –115.8 (m). **HRMS** (ESI) exact mass calcd. for $C_{21}H_{22}F_2NO_4$ ($[M + H]^+$) requires m/z 390.1511, found m/z 390.1522. **HPLC** (Chiralpak ADH, *i*-propanol/hexane = 40/60, flow rate 1.0 mL/min, 40 °C, λ = 254 nm): t_R (major) = 6.7 min; $[\alpha]^{25}_D$ = -36.4 (*c* = 1.0, DCM).

Crystal Structure of Fmoc-2l

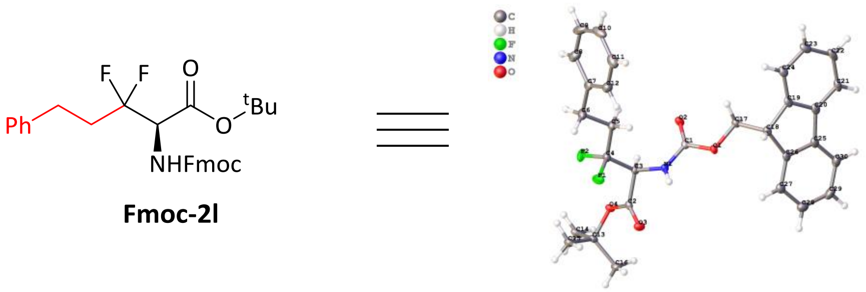

Identification code	exp_4769
Empirical formula	C30H31F2NO4
Formula weight	507.56
Temperature / K	105.7
Crystal system	monoclinic
Space group	P21
a / Å, b / Å, c / Å	13.6459(9), 5.1254(3), 18.1098(12)
α/°, β/°, γ/°	90, 94.055(6), 90
Volume / Å3	1263.44(14)
Z	2
ρcalc / mg mm-3	1.334
μ / mm□1	0.808
F(000)	536
Crystal size / mm3	0.400 × 0.350 × 0.300
2Θ range for data collection	7.852 to 142.206°
Index ranges	-16 ≤ h ≤ 16, -6 ≤ k ≤ 6, -22 ≤ l ≤ 14
Reflections collected	8233
Independent reflections	4766[R(int) = 0.0199 (inf-0.9Å)]
Data/restraints/parameters	4766/1/338
Goodness-of-fit on F2	1.047
Final R indexes [I>2σ (I) i.e. Fo>4σ (Fo)]	R1 = 0.0299, wR2 = 0.0770
Final R indexes [all data]	R1 = 0.0303, wR2 = 0.0772
Largest diff. peak/hole / e Å-3	0.224/-0.212
Flack Parameters	0.01(6)
Completeness	0.998
Identification code	exp_4769
Empirical formula	C30H31F2NO4
Formula weight	507.56
Temperature / K	105.7

General methods for manual peptides synthesis

All used peptides in this study were achieved by the 9-fluorenyl-methoxycarbonyl (Fmoc) based on the solid phase peptide synthesis (SPPS) by manually.

Linear WT Oxytocin synthesis:

The corresponding amount of resin for a 0.1mmol scale was swollen in DMF/DCM (1:1, v/v) for 25 min. After 25 min of swelling, the resin was washed with DMF (3 times), DCM (3 times), and DMF (3 times). The first Fmoc amino acid (4.0 equiv. to resin loading) and HCTU (3.9 equiv.) were added to DMF (6ml), then followed by adding DIPEA (8 quiv.) The mixture was pre-activated for 0.5 min. Afterwards, the resulting reaction mixture was added to the resin and shaken for 1h at r.t. Then, the resin was filtered and wash with DMF (3 times), DCM (3 times), and DMF (3 times). In order to remove Fmoc protecting group, piperidine (20% in DMF) was

added to the resin and reacted for 20 min (double: 10 min and 10 min). After finishing the assembly of peptide, the resin was treated with a TFA cleavage cocktail (TIPS/H_2O/EDT/TFA = 1/2.5/2.5/94, v/v/v/v) for 3h at r.t., to cleave the peptide off from the resin. The resin was washed with TFA. The combined solutions were concentrated by evaporation under a stream of nitrogen. The resulting crude peptide was obtained by precipitating with cold diethyl ether, and isolated by centrifugation. The crude peptide was dissolved in H_2O/ACN (1:1, v/v), purified by semi-preparative HPLC and analyzed by UPLC-UV/MS (yield: 28.3% after semi-preparative HPLC and lyophilization).

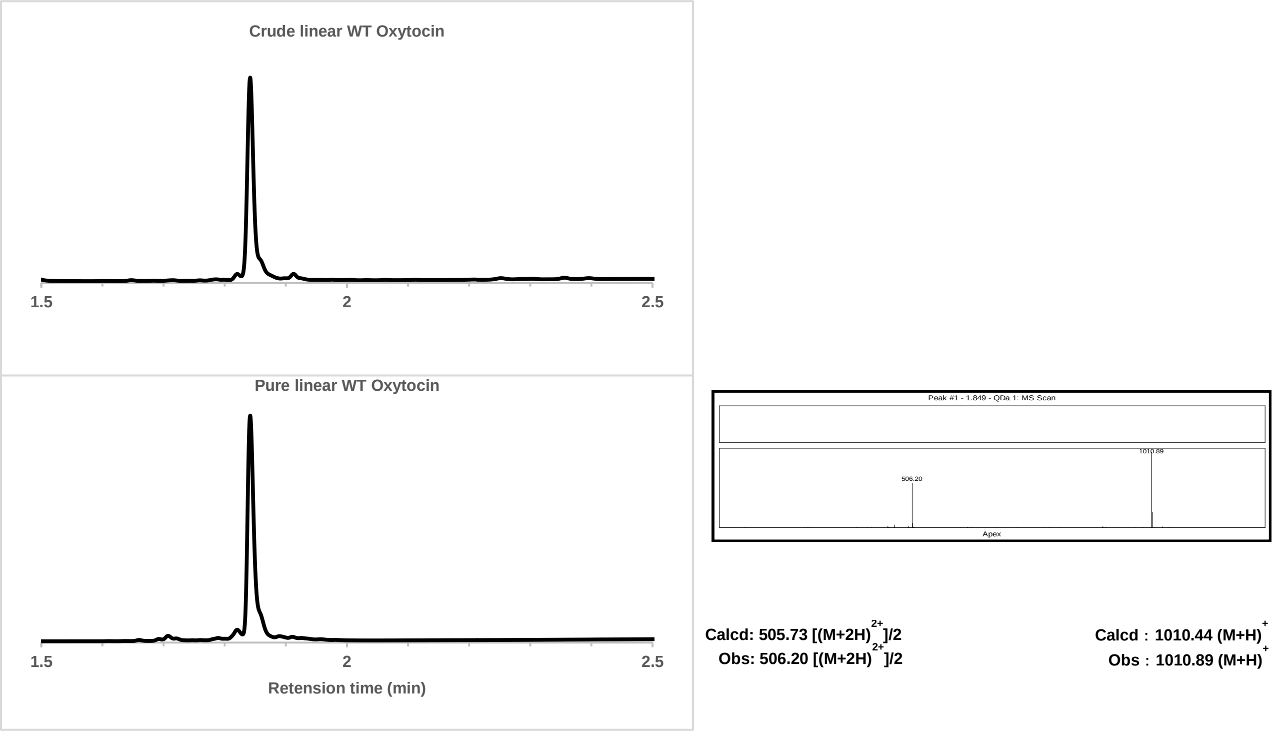

Supplementary Figure 120. Analytical UPLC-UV/MS of linear WT Oxytocin

Linear difluoro-Oxytocin synthesis:

The corresponding amount of resin for a 0.1mmol scale was swollen in DMF/DCM (1:1, v/v) for 25 min. After 25 min of swelling, the resin was washed with DMF (3 times), DCM (3 times), and DMF (3 times). Fmoc protecting group was removed by piperidine (20% in DMF) and was reacted for 20 min (double: 10 min and 10 min). For coupling the fluorinated amino acid, a solution was prepared of the suitable amount of Fmoc-protected amino acid (1.5 equiv. relative to resin loading), HOBt (1.5 equiv.), and DIC (1.5 equiv.) in minimal DMF. The resulting mixture was agitated for 20 min at r.t., then added to the resin and shaken for 16h at r.t. Afterwards, the resin was drained and washed with DMF (3 times), DCM (3 times), and DMF (3 times). Then, Fmoc protecting group was removed by piperidine (10% in DMF) and was reacted for 6 min (double: 3 min and 3 min). For coupling the commercial amino acids, a solution was prepared of the appropriate amount of Fmoc-protected amino acid (3 equiv. relative to resin loading) and HBTU (2.9 equiv.) in minimal DMF, then followed by adding DIPEA (6 equiv.). The mixture was immediately added to the resin and shaken for 1.5h at r.t. Then, the resin was filtered and wash with DMF (3 times), DCM (3 times), and DMF (3 times). Again, the Fmoc

protecting group was removed by piperidine (10% in DMF) and was reacted for 6 min (double: 3 min and 3 min). After finishing the assembly of peptide, the resin was treated with a TFA cleavage cocktail (TIPS/H_2O/EDT/TFA = 1/2.5/2.5/94, v/v/v/v) for 3h at r.t., to cleave the peptide off from the resin. The resin was washed with TFA. The combined solutions were concentrated by evaporation under a stream of nitrogen. The resulting crude peptide was achieved by precipitating with cold diethyl ether, and isolated by centrifugation. The crude peptide was dissolved in H_2O/ACN (1:1, v/v), and purified by semi-preparative HPLC and analyzed by UPLC-UV/MS (yield: 29.6% after semi-preparative HPLC and lyophilization).

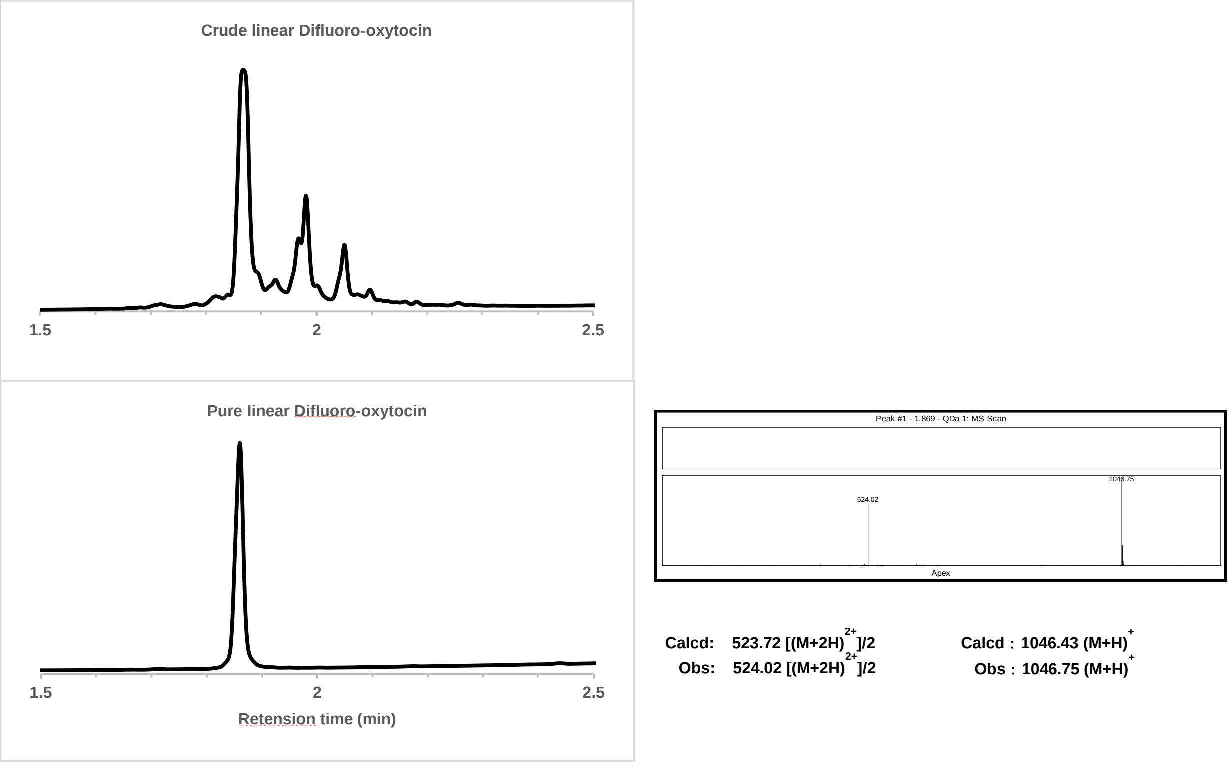

Supplementary Figure 121. Analytical UPLC-UV/MS of linear difluoro-Oxytocin

Folding of disulfide-containing peptides:

Synthetic peptides were dissolved in Tris buffer (0.1 M Tris, 10 equiv. GSSG, pH 7.5) for the oxidative folding. The final peptide concentration was 1mg/ml. The folding reaction was gently stirred and performed overnight at r.t. The main peak was purified by semi-preparative HPLC and analyzed by UPLC-UV/MS. (Yield of folded WT-Oxytocin: 56.4% after semi-preparative HPLC and lyophilization; yield of folded difluoro-Oxytocin: 63.1% after semi-preparative HPLC and lyophilization).

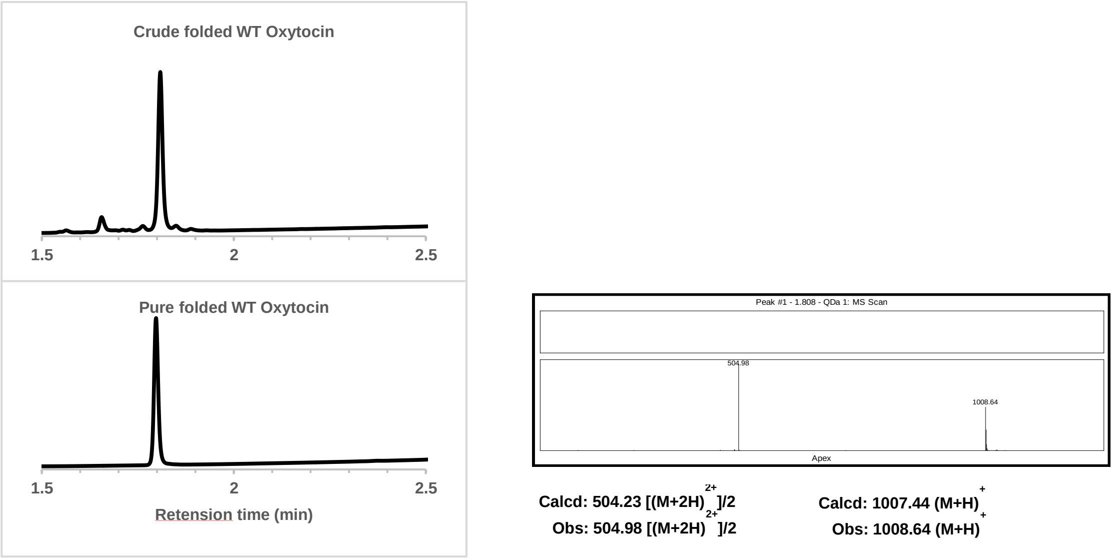

Supplementary Figure 122 Analytical UPLC-UV/MS of folded WT Oxytocin

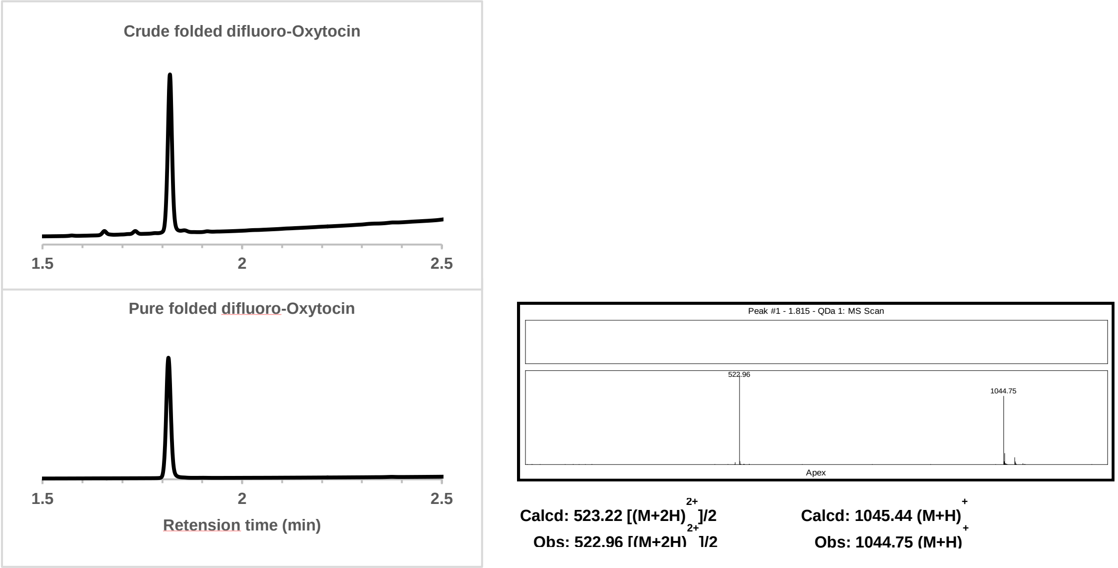

Supplementary Figure 123 Analytical UPLC-UV/MS of folded difluoro-Oxytocin

The stability tests of fold Difluoro-oxytocin and its corresponding WT Oxytocin in GSH

In order to investigate the stability issue of fold Difluoro-oxytocin (S-Figure 2) and its corresponding WT Oxytocin (S-Figure 1) (0.25mM), we treated them with 3mM (12 equiv.) of glutathione (GSH) in neutral buffer (100mM PBS, pH 7.0), respectively. GSH contains three amino acids, glutamate, cysteine, and glycine, which can be regarded as a reductant, and exists in the cytoplasm of health cells with the corresponding concentrations between 0.2 and 10 mM. Meanwhile, this reductive environment will affect any peptides, which occupying poor stability

of disulfide bonds, and generate uncertainties in the pharmacological studies.

As shown in S-Figure 5, in WT Oxytocin, peak s1 represented two molecules of GSH conjugated onto the reduced disulfide bond as observed in ESI-MS. Both peaks of s2 and s3, which gave the same value, demonstrated one molecule of GSH conjugated onto either the thiol group of the reduced disulfide bond. Peak s5 represented linear WT Oxytocin. Regarding peaks from s6 to s9, we observed the undesired diastereomers because all the four peaks gave the identical value, which demonstrated the re-folding process of linear WT Oxytocin. On the other side, the stability test of Difluoro-oxytocin gave the similar pattern of peaks distribution as observed in Analytical UPLC. (S-Figure 5). All the peaks gave additional apostrophe in order to be distinguished with the ones as observed in WT Oxytocin.

Although the disulfide bonds in both fold Difluoro-oxytocin and fold WT Oxytocin are opened gradually under 3mM GSH condition within 40 min, the growth rate of peak s5’ in Difluoro-oxytocin, which represent of generated linear Difluoro-oxytocin, is slower compared with its corresponding peak s5 under the same condition. For example, the disulfide-bond scrambling, peak s5’, in F-Oxytocin can reach to around 50% after 20 min. However, this scrambling value, peak s5, will be arising to nearly 2/3 in WT Oxytocin at the same time. Additionally, we can see a significantly different intensity between peak s4’ (fold difluoro-Oxytocin) and s5’ (regenerated linear Difluoro-oxytocin) in Difluoro-oxytocin after 30min instead of nearly equal intensity of peaks s4 (fold WT-Oxytocin) and s5 (regenerated linear WT Oxytocin) in WT Oxytocin at the same time.

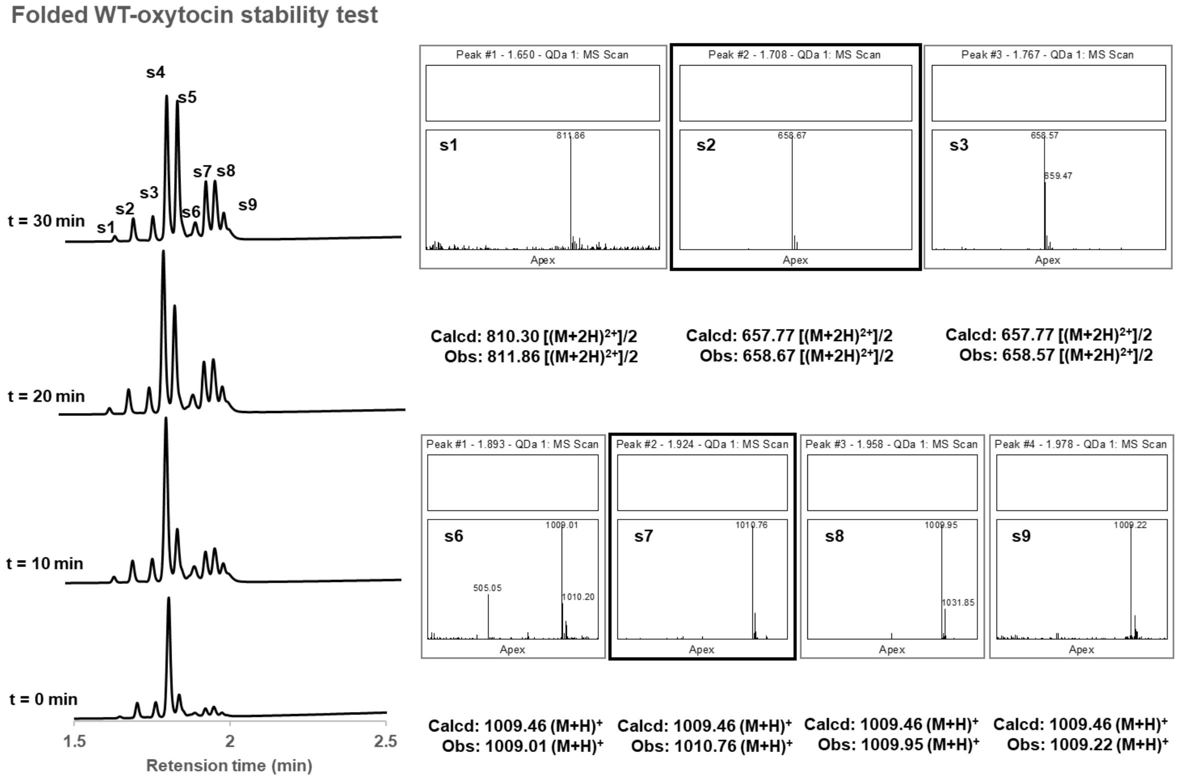

Supplementary Figure 124 Folded WT-oxytocin stability test

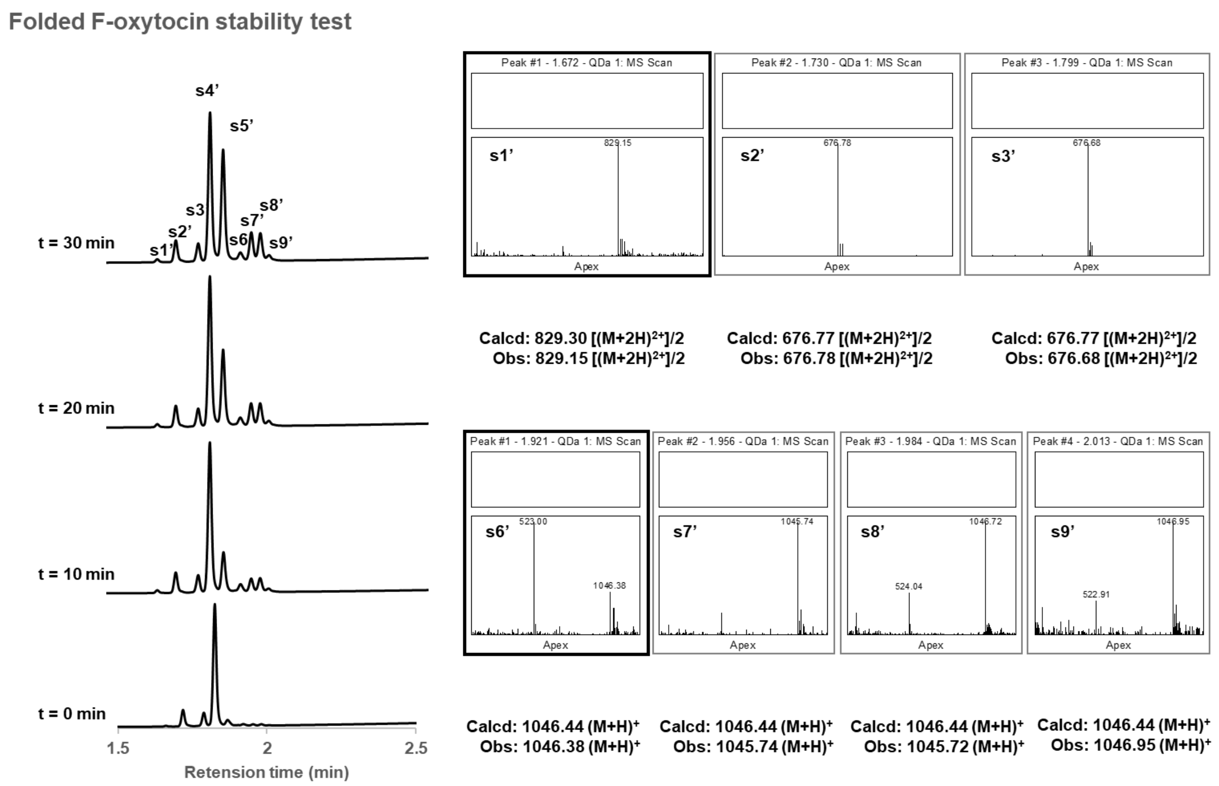

Supplementary Figure 125 Folded WT-oxytocin stability test

Supplementary References

1 Wehn, P. M. & Du Bois, J. A Stereoselective Synthesis of the Bromopyrrole Natural Product (−)-Agelastatin A. *Angew. Chem. Int. Ed.* **48**, 3802-3805 (2009).

2 Zhou, Y. *et al.* Cobalt-Bisoxazoline-Catalyzed Enantioselective Cross-Coupling of α-Bromo Esters with Alkenyl Grignard Reagents. *Org. Lett.* **22**, 4532-4536 (2020).

3 Pitzer, L., Schäfers, F. & Glorius, F. Rapid Assessment of the Reaction-Condition-Based Sensitivity of Chemical Transformations. *Angew. Chem. Int. Ed.* **58**, 8572-8576 (2019).

4 Shendage, D. M., Fröhlich, R. & Haufe, G. Highly Efficient Stereoconservative Amidation and Deamidation of α-Amino Acids. *Org. Lett.* **6**, 3675-3678 (2004).

5 Miao, J., Yang, K., Kurek, M. & Ge, H. Palladium-Catalyzed Site-Selective Fluorination of Unactivated C (sp3)–H Bonds. *Org. Lett.* **17**, 3738-3741 (2015).
