# Supplementary material for: Biomimetic enantioselective synthesis of β,β-difluoro-α-amino acid derivatives
Source: Commun Chem. 2021 Oct 22;4:148. doi: 10.1038/s42004-021-00586-z (PMC9814941; doi:10.1038/s42004-021-00586-z)
Supplement: Supplementary file 3 — Description of Additional Supplementary Files [file 42004_2021_586_MOESM3_ESM.pdf]

Description of Additional Supplementary Files

File Name: Supplementary Data 1
Description: crystallographic cif data of **Fmoc-2I**
